# *“You're really walking along the razor's edge”*: A meta-synthesis on the existential cost of breast cancer related to financial toxicity

**DOI:** 10.1016/j.breast.2026.104705

**Published:** 2026-01-20

**Authors:** Sara Paltrinieri, Margherita Schiavi, Barbara Bressi, Angela Contri, Martina Torreggiani, Laura Bernardi, Elisa Mazzini, Maria Chiara Bassi, Silvia di Leo, Luca Ghirotto, Stefania Costi

**Affiliations:** a Research and EBP Unit, Health Professions Department, Azienda USL-IRCCS di Reggio Emilia, Via Giovanni Amendola, 2, 42122 Reggio Emilia, Italy; b Clinical and Experimental Medicine PhD Program, University of Modena and Reggio Emilia, Via del Pozzo n.74, 41100 Modena, Italy; c IRCCS Istituto delle Scienze Neurologiche di Bologna, Via Altura n.3, 40139 Bologna, Italy; d Physical Medicine and Rehabilitation Unit, Azienda USL-IRCCS di Reggio Emilia, Via Giovanni Amendola, 2, 42122 Reggio Emilia, Italy; e Scientific Directorate, Azienda USL-IRCCS di Reggio Emilia, Via Giovanni Amendola, 2, 42122 Reggio Emilia, Italy; f Medical Library, Azienda USL-IRCCS di Reggio Emilia, Via Giovanni Amendola, 2, 42122 Reggio Emilia, Italy; g Psycho-Oncology Unit, Azienda USL-IRCCS di Reggio Emilia, Via Giovanni Amendola, 2, 42122 Reggio Emilia, Italy; h Qualitative Research Unit, Azienda USL-IRCCS di Reggio Emilia, Via Giovanni Amendola, 2, 42122 Reggio Emilia, Italy; i Department of Surgery, Medicine, Dentistry and Morphological Sciences, University of Modena and Reggio Emilia, via del Pozzo, 71, 41124 Modena, Italy

**Keywords:** Breast neoplasms, Cancer-related financial toxicity, Qualitative research, Systematic review, Meta-synthesis

## Abstract

Cancer-related financial toxicity (FT) is a challenge of living with and beyond breast cancer (BC). A systematic review and meta-synthesis was conducted to report the experience of individuals with BC regarding cancer-related FT. Data were searched in MEDLINE, Embase, Cinahl, Scopus, PsycINFO, and Web of Science from inception. Eligibility was restricted to original qualitative studies. We performed a meta-synthesis by generating interpretative themes and a model of cancer-related FT. Thirty-two studies were included, encompassing 1080 individuals with BC. Of these studies, 17 were conducted in North America (719 individuals), nine in South, East, and West Asia (238 individuals), two in Oceania (53 individuals), and four in Northwest Africa (70 individuals). Five themes were identified: (i) the existential cost of cancer, (ii) the impact of insurance complexity, (iii) the need for timely and accessible information, (iv) seeking possible help, (v) negotiating daily life. The review highlights the lack of original qualitative studies conducted in Europe. Socioeconomic status, insurance, and employment amplify inequalities and shape the experience of FT. The interpretative model could support individuals with BC and providers’ communication.

## Highlights

• Individuals with breast cancer experience cancer-related financial toxicity.
• We provided an interpretative model to describe their experiences.
• This model improves communication between patients and professionals.
• Tailored information and a navigator of economic aspects are patients' needs.
• Primary qualitative studies across European countries are needed.

## Graphical abstract

**Figure:**
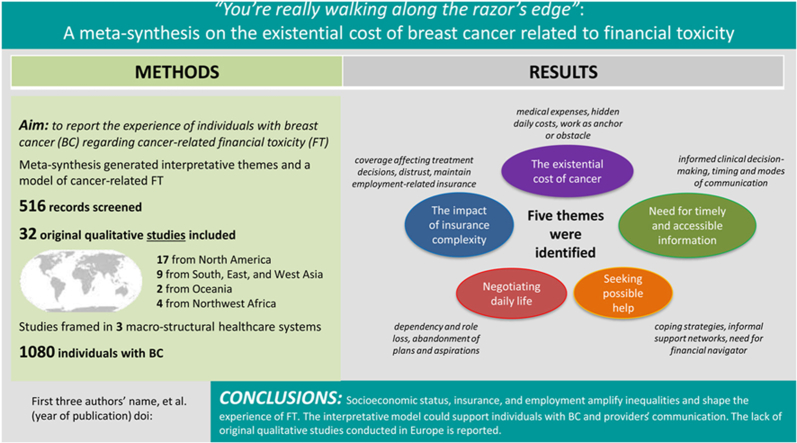

## 1 Introduction

Breast cancer (BC) is the second most frequent tumour worldwide, with 2.3 million new cases and eight million prevalent cases in 2022 [1]. BC is the third tumour type with the highest economic costs and the first one in females [2]. Early diagnostic methods, effective treatments, and changes in population growth will contribute to the progressively increasing trend [1].

Cancer-related financial toxicity (FT) is a major challenge for individuals living with and beyond BC [3]. Defined as both objective financial burden and subjective distress from cancer treatment [4,5], FT can affect access to care [6], financial stability, and overall health [7]. It involves out-of-pocket medical expenses, non-medical costs like transportation, and income loss due to sick leave and/or job loss [8]. FT varies by country, depending on the healthcare system characteristics, such as coverage, out-of pocket costs, and social protection mechanisms [9]. For example, the lack of universal coverage shifts costs to patients, often resulting in financial hardship or increased reliance on family and social support networks. Moreover, while FT is more common in low- and middle-income countries, over 30 % of individuals with BC in high-income countries also report FT [9]. Psychological effects, such as anxiety, fear of bankruptcy, and guilt over burdening family, can worsen FT [10], especially when caregivers lose income [11]. FT often leads to coping strategies like cutting essential spending [12] or skipping care [11].

Although cancer type is not a clear FT risk factor [9], individuals with BC may face greater financial needs due to prolonged multimodal treatments and heterogeneous side effects [10], such as lymphedema [11] or the need for breast reconstruction [12]. Furthermore, individuals with BC who are younger in age, have Hispanic ethnicity in the United States context, lower income [13,14], and are migrants [15] seem to have a higher risk of FT. Moreover, as compared to their male counterparts, females have a lower employment rate, particularly in low-income countries, work fewer hours per week on average, which affects their incomes [16], and are at higher risk of unemployment [17]. These social disparities are further exacerbated by the unequal distribution of non-paid work (i.e., household management, children) [18].

Over the past decades quantitative and qualitative studies have addressed cancer-related FT in individuals with BC [19,20]. Although qualitative research explores complex experiences shaped by social and cultural factors, no systematic review has focused on the broad lived experience of cancer-related FT and its daily impact on individuals with BC.

Gathering patients’ perspectives from diverse contexts can reveal the deeper meaning of cancer-related FT. Understanding their experiences is imperative to plan tailored interventions to reduce FT.

This systematic review and meta-synthesis addressed the following research questions: i) what is the experience of individuals with BC concerning their cancer-related FT? and ii) what are the reported consequences on daily life?

We aimed to synthetize the experiences of living with the cancer-related socioeconomic consequences from the perspectives of individuals with BC.

## 2 Methods

### 2.1 Search strategy and selection criteria

This systematic review and meta-synthesis is reported according to the ENTREQ guidelines (Appendix A, p 1) [21]. It was prospectively registered in PROSPERO (CRD42024534127). Bibliographic search was pre-planned and conducted by an information specialist (M.C.B.), a qualitative methodologist (L.G.), and two healthcare professionals (S.*P. and* S.C.). The search strategy (Appendix B, p 3) was customized for the electronic databases MEDLINE, Embase, Cinahl, Scopus, PsycINFO, and Web of Science, and they were searched from inception to May 2025. Filters were not applied. Records were also identified through citation searching. We included studies involving adult individuals (≥18 years) diagnosed with BC, regardless of disease stage or survivorship phase, that reported findings on cancer-related FT and its impact on daily life. We included any original study using either a primary or secondary data analysis in which qualitative methodology was used to collect data. We excluded studies with mixed cancer populations and mixed method studies when it was not possible to clearly identify the voice of individuals with BC or to extract the qualitative data, respectively. We excluded studies that were not published in English.

Three researchers (S.P., B.B., and M.T.) conducted title and abstract screening using Rayyan to manage the process [22], and any disagreement was resolved with S.C.

### 2.2 Quality assessment

The methodological quality of the included reports was assessed independently by two reviewers (S.*P. and* L.B.), and any disagreement was resolved with S.C. Appraisal was performed by using the Critical Appraisal Skills Program (CASP) checklist for qualitative research [23,24]. This checklist assesses the methodological quality of qualitative studies based on eleven criteria, including the validity of findings, appropriateness of the qualitative methodology and study design, recruitment strategy, theoretical framework, data collection methods, researcher-participants’ relationship, ethical considerations, data analysis, clarity of results, and the overall value of the research. The CASP checklist provides an overall quality level with no cutoff score.

### 2.3 Healthcare system contextualization

We framed the included reports within macro-structural contexts according to the widely accepted categorization proposed by Bohm et al. (2013) grounded in the theoretical assumptions developed by Wendt et al. (2009) to define healthcare system models [25,26]. The framework is primarily based on financing and healthcare coverage, two pillars of the regulatory dimension, one of the key criteria used to classify healthcare systems. Coverage refers to the inclusion of the population, in whole or in part, within public and/or private healthcare systems, while financing refers to the funding of healthcare through public and/or private sources. Included reports were framed in the following macro-structural contexts:

• private health system is predominantly coordinated by private market actors, does not guarantee universal coverage, and relies on fragmented private insurance schemes or out-of pocket payments;
• national health system is predominantly based on universal health coverage, with residual out-of-pocket costs. Although formal coverage exists, indirect payments and services not covered by the system persist,
• under-resourced or poorly institutionalized health system is characterized by limited social protection and a high reliance on informal networks.

This categorization was intended as a contextual framework to support the interpretation of the findings, acknowledging that participants' experiences of FT are shaped by, though not explicitly articulated in terms of, these macro-structural factors.

### 2.4 Data extraction and meta-synthesis

Data were extracted independently by three authors (S.P., S.C, and L.B.), who agreed in advance on the information to be selected. The following data were extracted from each of the included reports: country of origin and healthcare system, journal title, study's aim(s) and design, recruitment setting, sampling strategy, data collection period, strategy and setting, data analysis, summary of findings, and sample characteristics.

In the context of this systematic review, we adopted a meta-synthesis approach to synthesize data, consistent to produce an interpretative integration and re-elaboration of qualitative findings from original studies. Meta-synthesis is a well-established methodology that transcends the mere aggregation of findings, instead offering a higher-order conceptualization through which novel interpretative insights can emerge [[27], [28], [29]]. Given the complexity and multifaceted nature of cancer-related FT, which intersects with structural, psychological, and relational domains, an interpretative approach was necessary to capture the depth and nuance of participants’ experiences. The phenomenon of FT in individuals with BC is not reducible to financial burdens alone, but involves identity negotiations, existential distress, and socio-economic inequalities. Thus, a meta-synthesis allowed us to move beyond listing barriers or coping mechanisms, and instead to identify deeper, latent constructs that shape how individuals experience, make sense of, and respond to FT.

To perform the meta-synthesis, both the quotes of individuals with BC (first order constructs) and the interpretation of the authors (second order constructs) of each original study were considered for extraction. The quotes reported under the headings “results” of each study were manually extracted and reported in an Excel table. During a meeting, S.P., B.B., M.S., A.C., and S.C. collaboratively coded the quotes of two original studies using line-by-line coding of the first and second order constructs to identify labels. Then, a third study was coded independently, and labels compared. The remaining studies were coded independently into existing or new labels. Subthemes and themes were inductively established by grouping labels and interpreting their meanings into a interpretative model. Disagreements were resolved with L.G. and S.D.L. The dataset is available in the repository.

## 3 Results

**Fig. 1:**
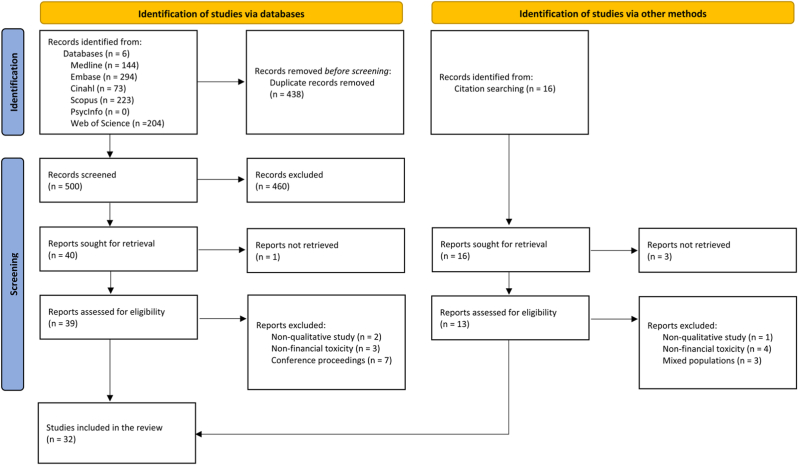
PRISMA flowchart.

The PRISMA flowchart (Fig. 1) reports retrieval and screening process. We retrieved 938 records from the selected databases. After removal of duplicates, 500 titles and abstracts were screened, of which 40 reports were sought in full text. Sixteen additional reports were identified through citation searching. Full-text reports were assessed for eligibility, of which 24 were excluded. The latter are referenced in appendix C (p 5) together with the reasons for their exclusion.

Table 1 summarizes data collected from the 32 studies included, which were conducted in Australia [30,31], Canada [32], China [33], Egypt [34], Ghana [35], India [[36], [37], [38]], Indonesia [39], Iran [40], Malaysia [41], Nigeria [42,43], Pakistan [44], the USA [[45], [46], [47], [48], [49], [50], [51], [52], [53], [54], [55], [56], [57], [58], [59], [60]], and Vietnam [61]. Twenty-four studies were qualitative [[30], [31], [32], [33],^35^,^36^,[39], [40], [41], [42], [43], [44], [45], [46],[49], [50], [51], [52], [53],^55^,[57], [58], [59], [60]], while seven had a mixed methods design [34,37,38,47,48,54,56], and one was an ethnography [61]. Five studies restricted eligibility to individuals with metastatic BC [31,39,53,58,60]. Sixteen studies used purposive sampling [32,36,37,39,40,42,43,[47], [48], [49],[51], [52], [53],^55^,^57^,^59^,^60^], six used convenience sampling [34,38,41,45,50,56], and five used mixed methods sampling designs [30,31,33,35,44,61]. Most studies used the individual semi-structured interview to collect data [30,31,[33], [34], [35], [36], [37],^39^,^40^,[42], [43], [44],[47], [48], [49], [50], [51],^53^,^54^,[56], [57], [58], [59], [60]]. A few other studies used focus group [32,38,41,45,46] or a mixed methods data collection modality [52,55,61].

**Table 1:** Study characteristics.

First author	Country and Healthcare system	Journal title	Aim(s) of the study	Study design	Recruitment setting	Eligibility criteria		Sampling strategy	Data collection period	Data collection strategy	Data collection setting	Data analysis	Summary of findings
						*Inclusion*	*Exclusion*						
Ko (2025)	USA Private health system	BMC Women's Health	to explore financial toxicity, relating to what worsens the financial situation and its impacts, from the perspectives of Latina BC patients, family caregivers, and healthcare professionals	qualitative	local community organization using the patient database and referrals from local oncology clinics	diagnosed with BC within the past five years, self-identified Latina, ≥18 years	··	purposive	Nov 2022–Jun 2023	in-depth interview	mixed (remote or in presence)	thematic analysis	employment disruption, medical-related and non-medical related financial costs, struggle to meet basic needs and experienced psychological distress, coordinating patient support care to accommodate their financial needs negatively impacted their families' work schedules and routines, affecting family relationships
Marshall (2025)	USA Private health system	Nursing Research	to examine the lived experiences of women diagnosed with metastatic BC in the realm of financial concerns and unmet support needs specific to their cancer treatment	qualitative	designated cancer center located in southern United States	metastatic BC, ≥18 years, English speaking	··	purposive	··	semi-structured interview	remote (telephone or video call)	thematic analysis	Financial toxicities (costs of cancer treatments and medications, limitations of health insurance, effect on employment); unmet support needs (managing usual responsibilities, emotional, community and organizational support)
Do (2024)	Vietnam Poorly institutionalized system	Social Science & Medicine	to examine how BC patients manage the financial burden of cancer care in Vietnam	ethnographic	Oncology Centre of Hue Central Hospital	··	··	mixed (snowball and convenience)	Apr–Dec 2019	mixed (observation, interviews, focus groups)	presence	grounded theory approach	in a context where health-related risk protection is poorly organized and out-of-pocket expenses are burdensome, despite the presence of universal health coverage, patients must rely heavily on informal arrangements to finance their treatment, proactively researched available information and undertook extensive and ramified work to prove their deservingness for some types of assistance (strategically disclosing cancer)
Jones (2024)	USA Private health system	Future Science	to refine the conceptual model of financial hardship after cancer	qualitative	Survivorship Program at anNCI-designated Comprehensive Cancer Center	diagnosed with BC, ≥18 years, being at least 3 months post-diagnosis, able to read and speak English and to provide informed consent, located in the USA	··	convenience (participants were recruited among 162 women who consented to be contacted about future studies)	··	semi-structured interview	remote (telephone or video call)	deductive framework approach	financial hardship: protective factors included good health insurance, work accommodations and social support. Participants worried about cancer care costs and employment. Programs for alleviating financial hardship had high administrative burdens
Khajoei (2024)	Iran Poorly institutionalized system	BMC Cancer	to investigate the needs and experiences of breast cancer survivors	qualitative	University Hospital in Tehran	women diagnosed with BC, 18–60 years, who had completed their treatment courses between four and 18 months prior, non-metastatic cancer, and stable clinical conditions	cancer recurrence or metastasis, secondary cancers, and cognitive impairment	purposive	Apr–Jul 2023	semi-structured interview	mixed (remote or in presence)	Inductive content analysis	financial toxicity, family support, informational needs, and psychological and physical issues
Patra (2024)	India Poorly institutionalized system	Cureus	to examine the burden of financial hardship the causes and repercussions among BC survivors.	mixed methods	Indian Breast Cancer Survivorship Conference	··	··	convenience (participants were recruited among 150 BC survivors who attended the conference)	Oct 2023	focus group	presence	thematic framework	Financial challenges, unemployment, borrowed money, treatment-related financial issues, financial insufficiency, discontinued treatment, insurance disparities and coverage, information and awareness, financial literacy
Ruan (2024)	China National health system	Supportive care in cancer	to delve into the coping process amidst financial toxicity among young women with breast cancer	qualitative	Fudan University Shanghai Cancer Center	diagnosis of BC, 18–40 years, completion of primary treatment and health condition was stable	severe systemic conditions, ECOG score of 3–5, psychiatric disorders, hearing or expression barriers, identified by medical documents or observed by researchers during initial communication	mixed (purposive and theoretical)	Sept 2021–Jan 2022	semi-structured interview	in presence	Strauss & Corbin's constant comparative analysis	financial toxicity is related to risk factors, coping resources, and unmet needs. To overcome financial toxicity, participants adjusted by reshaping consumption concept, re-dividing of family functions, re-planning of occupation career, and rebuilding life confidence
Waters (2024)	USA Private health system	Cancer Causes & Control	to understand patients' lived experiences of financial burden, accessing financial assistance, and cost-coping mechanisms	qualitative	fliers in the breast cancer clinic, restrooms, and other areas within an academic medical center in the Southeast and clinical staff	patients = diagnosed with metastatic BC, ≥18 years, spoke English, reported having experienced financial burden caregiver = study enrollment was expanded to include caregivers acting as proxies on behalf of patients	··	··	Jan–Jul 2019	semi-structured interview	mixed (remote or in presence)	descriptive phenomenological approach to thematic analysis	causes of financial burden, financial assistance mechanisms, health insurance and financial burden, and cost-coping behaviors
Khazi (2023)	India Poorly institutionalized system	Clinical Epidemiology and Global Health	to explore the psychosocial impact of breast cancer diagnosis on women in North Karnataka and the coping strategies adopted by them	qualitative	Belgaum Cancer Hospital	women diagnosed with BC and undergoing treatment	··	purposive	··	semi-structured interview	in presence	interpretative phenomenological analysis	psychological effects at diagnosis: stress and uncertainty, financial distress, family distress, and social withdrawal. Family support and religious belief were the main coping strategies
Kolawole (2023)	Nigeria Poorly institutionalized system	African Journal of Reproductive Health	to explore the lived experience of breast cancer patients at the General Hospital, Ilorin, Kwara State, Nigeria	qualitative	General Hospital Ilorin, Kwara State	patients with BC, ≥30 years, have completed the initial treatment or fully completed treatment	··	purposive	Dec 2021–Feb 2022	semi-structured interview	in presence	Tesch's content analysis approach using open coding	disease's socioeconomic impact includes poor human relations, negative perceptions of breast cancer diagnosis, poor sociocultural roles, and negative effects on patients' livelihood
Lee (2024)	USA Private health system	Supportive care in cancer	to reduce the gap of limited research by characterizing and describing treatment-related FT through the lived experiences of younger, rural, Black, and low-income women diagnosed with breast cancer	qualitative	National Cancer Institute Designated Comprehensive Cancer Center	stage I–IV breast cancer, ≥18 years, received at least one primary cancer treatment modality (e.g., surgery, chemotherapy, radiation therapy, and/or endocrine therapy)	··	purposive	Sept 2021–Mar 2022	mixed (group and individual interview)	remote (telephone or video call)	descriptive coding and thematic analysis	short-term and long-term impacts of financial toxicity
Neilson (2023)	USA Private health system	Journal of Surgical Research	to characterize contributors to financial distress in breast cancer patients, to assess resources used to combat financial toxicity, and to determine the preferred timing of education regarding potential financial effects of treatment	mixed methods	academic institution	invasive BC or ductal carcinoma in situ	··	··	Oct–Dec 2020	semi-structured interview	remote (telephone or video call)	grounded theory methodology	contributors to financial distress: unexpected medical and nonmedical expenses, lost revenue from missed work, and altered budgeting
Walton (2023)	India Poorly institutionalized system	Indian Journal of Palliative Care	to explore the experiences of adult female cancer survivors, to discover and describe common protective resilience factors that helped them to cope with the cancer experience and to identify the barriers to resilience	mixed methods	Cancer Survivors and Healthcare Personnel	BC survivors, 18–70 years, had completed treatment, symptom free for the past 3 months and not exceeding 3 years	metastasis, terminally ill and with comorbidities	purposive	··	semi-structured interview	in presence	Colaizzi's data analysis framework	protective resilience factors and barriers to resilience
Aitken (2022)	Australia National health system	Healthcare	to understand the journey undergone by women, diagnosed with breast cancer, from diagnosis to survivorship, and to explore the challenges experienced by the BC survivors due to physical change, financial hardship, emotional distress and social isolation	qualitative	organization Pink Hope, flyers, advertising on social media, word of mouth	BC survivors diagnosed for at least a year, ≥35 years	diagnosed less than one year ago, diagnosed greater than 11 years prior	mixed (snowball and convenience, social media and community organization advertising)	Aug–Sept 2021	semi-structured interview	remote (telephone or video call)	thematic analysis	psychological distress: process of diagnosis, undergoing treatment and isolation post-treatment
Marshall (2022)	USA Private health system	Supportive care in cancer	to explore the cancer treatment–specific medication beliefs among women undergoing active cancer treatment for metastatic breast cancer	qualitative	designated cancer center located in southern United States	metastatic BC, ≥18 years, English speaking, receiving active cancer treatment, able to complete interviews via telephone or Zoom	··	purposive	··	semi-structured interview	remote (telephone or video call)	thematic analysis	positive cancer treatment–specific medication beliefs highlighting the benefit of treatment, negative cancer treatment–specific medication beliefs that caused concern for cancer treatment, dialectical cancer treatment–specific medication beliefs indicating the benefits of cancer treatment outweigh the risks
Prabandari (2022)	Indonesia Poorly institutionalized system	The Breast	to explore the narratives of patients with BC to understand their experiences of accessing and receiving care from diagnosis and undergoing cancer treatments	qualitative	Dr. Sardjito Hospital, Yogyakarta, Indonesia	metastatic breast cancer, not deemed too ill, had been recruited to the parent study within 12 months prior to data collection	··	purposive (from a parent study)	Jun–Nov 2019	semi-structured interview	In presence	thematic analysis	early experiences, prior to accessing health care, navigating the system to access treatment, enduring chemotherapy and advancing disease, with crucial family support, seeking normalcy and belief in treatment
Agha (2021)	Pakistan Poorly institutionalized system	Journal of International Women's Studies	to extend knowledge about the hurdles that bar BC patients from accessing the treatment and how patients and their families negotiate with the barriers led by BC	qualitative	··	women suffered from BC during past five years, belonged to rural or less privileged areas of Sindh province	··	mixed (purposive and snowball)	··	semi-structured interview	··	thematic analysis	knowledge, geographical, and financial barriers
Gharzai (2021)	USA Private health system	JCO Oncology Practice	to characterize patients' experiences of financial toxicity in the context of an established framework to identify knowledge gaps and strategies for mitigation	qualitative	participants who received aid from The Pink Fund, Michigan	actively undergoing BC treatment, who worked at the time of diagnosis, experienced loss of income, had a household income ≤500 % of federal poverty level	··	purposive	Feb–May 2020	semi-structured interview	remote (telephone or video call)	thematic analysis and framework method	objective burden: direct and indirect costs; subjective distress: material, psychosocial, and behavioral; novel addition to financial toxicity framework; gaps identified
Lewis (2021)	Australia National health system	Social Science & Medicine	to explore how women with incurable breast cancer experience and give meaning to choice in relation to their health (and beyond) in their daily lives	qualitative	··	women living with metastatic BC, Australian, ≥18 years	··	mixed (purposive and a community recruitment strategy)	Aug 2017–Jan 2020	semi-structured interview	mixed (remote or in presence)	constructionist approach to thematic analysis	‘choice’ as relational: the interpersonal dynamics of individuals' choices; the temporal tussle surrounding decisions of daily (and future) living; circumscribed choice/delimited agency
Oshima (2021)	USA Private health system	JCO Oncology Practice	to further characterize patient-reported financial experiences related to breast cancer treatment through analyzing rich qualitative data and using quantitative data to sort, stratify, and further contextualize participants' open-ended responses	mixed methods	Foundation Love Research Army and the Sisters Network of North Carolina	women with a prior diagnosis of BC, ≥18 years, living in the United States	male patients and those living outside the USA	convenience	Jan–Jun 2017	semi-structured interview	web-based survey	content analysis	financial impact due to direct costs for medical care; the financial arc and long-term impacts; discordance in stated financial burden; financial impact because of indirect costs; cost transparency and communication; navigating insurance
Chebli (2020)	USA Private health system	Support Care Cancer	to identify individual-, interpersonal-, community-, and organizational/healthcare policy-level factors that could contribute to financial toxicity among Latina breast cancer survivors	qualitative	Community of Chicago	diagnosis of invasive BC, receipt of a lumpectomy or mastectomy within the past five years, self-identification as being Latina, Hispanic, or Chicana, ≥18 years	··	convenience	Feb–Sept 2018	focus group	in presence	content analysis with deductive (theory-derived themes) and inductive approaches (themes emerging from iterative analysis)	individual-level: lack of knowledge and prioritization regarding financial aspects of care; interpersonal-level: social networks as important platforms for disseminating information; community-level: community norms and dynamics as important barriers; organizational-level: financial assistance programs' restrictive eligibility criteria, lack of coverage post-treatment, limited availability, and instability
Iddrisu (2020)	Ghana Poorly institutionalized system	Nursing Open	to explore the socioeconomic impact of breast cancer treatment and care on young women in Ghana	qualitative	three different hospitals	diagnosed with BC, undergone some form of treatment, 15–49 years, living in the Accra Metropolis, speak English and Twi	newly diagnosed patients and acutely sick patients on admission	mixed (purposive and snowball sampling)	··	semi-structured interview	in presence	content analysis	*perceptions and beliefs, economic concerns, and secrecy*
Kong (2020)	Malaysia National health system	The Oncologist	to gain an in-depth understanding of the financial needs following diagnosis of breast cancer in a middle-income setting with universal health coverage, with a focus on health costs, non-health costs, employment and earnings, and financial assistance	qualitative	five hospitals in Malaysia, of which two public and three private hospitals	Malaysian BC patients who were diagnosed at least 1 year prior to the study	Patients with carcinoma in situ and recurrent cancer	convenience	··	focus group	in presence	thematic analysis	health costs, non-health costs, employment and earnings, financial assistance
Dean (2019) SCC	USA Private health system	Support Care Cancer	to compares out-of-pocket costs for breast cancer survivors with and without lymphedema, integrating qualitative data to offer insight into what makes costs different comparing those with or without lymphedema	mixed methods	participants were recruited among participants in the quantitative study	women with stages I–III invasive breast cancer, completion of active breast cancer treatment, >1 lymph node removed, and current residents of Pennsylvania or New Jersey	active cancer or who were pregnant or planning to become pregnant in the next 6 months	purposive	··	semi-structured interview	in presence	Thematic coding	economic burden is cumulative and cascades over time; lymphedema care needs are unlikely to be covered by insurance; productivity losses have long-term impact
Dean (2019) Cancer	USA Private health system	Cancer	to provide a broader assessment of patient-driven recommendations by including diverse perspectives across age, insurance status, and race of BC survivors	mixed methods	participants were recruited among participants in the quantitative study	women with stages I–III invasive breast cancer, completion of active breast cancer treatment, >1 lymph node removed, and current residents of Pennsylvania or New Jersey.	active cancer, or who were pregnant or planning to become pregnant in the next 6 months	purposive	··	semi-structured interview	in presence	Thematic coding	expanding affordable insurance and insurance-covered items, especially for lymphedema treatment (among the 60 % who reported lymphedema); supportive domestic help; financial assistance from diagnosis through treatment; and employment-preserving policies
Japhet (2019)	Nigeria Poorly institutionalized system	Journal of Solid Tumors	to study the physical, financial, psychological and social impacts of BC on women in Northern Nigeria in order to add more knowledge to the existing literature which could be used as a reference tool for body of academia	qualitative	Federal Teaching Hospital Gombe and State specialist hospital Gombe	women diagnosed with BC, 18–75 years, any stage of the disease, at all the stages of treatment, from all the socio-economic background	··	purposive	··	semi-structured interview	··	thematic analysis	body problems, cost of treatment, emotional disturbances, relationship
Nolan (2019)	USA Private health system	Ethnicity & Health	to describe the lived experience of survivorship among young African American women who completed active treatment for breast cancer	qualitative	metropolitan and surrounding areas of Birmingham (Alabama), New Orleans (Louisiana), Jackson (Mississippi)	premenopausal African American women who completed active treatment for BC	previous diagnosis of cancer	purposive	··	mixed (semi-structured interviews, field and reflective notes, and personal effects)	mixed (remote or in presence)	transcendental phenomenology	actively managing spiritual self, actively managing physical self, actively managing psychological self, actively managing social self, and seeking survivorship knowledge
Pisu (2019)	USA Private health system	Annals of Internal Medicine	to explore the perspectives of patients and medical and nonmedical cancer center staff on costs of care conversations	qualitative	University of Alabama at Birmingham (UAB) Comprehensive Cancer Center	BC survivors, ≥60 years, cancer treatment at the UAB Comprehensive Cancer Center within the previous 5 years, English-speaking, physically and mentally able to participate, and residence in the Birmingham area	··	purposive	··	semi-structured interview	in presence	constant comparative method	content (reassurance, action, do not want to hear), timing (after diagnosis, when patient is ready), person (not the physician), characteristic (have time, compassionate, god communicator, knowledgeable)
McEwan (2014)	Egypt Poorly institutionalized system	Cancer Nursing	to deepen our understandings of women's experiences with and interpretations of, diagnosis and treatment delays and highlight nuances not identifiable in the quantitative study	mixed methods	participants were recruited among participants in the quantitative study	··	··	convenience (participants were recruited among participants in the quantitative study)	··	semi-structured interview	··	thematic and content analysis	interpersonal relationships, institutional factors, public policy factors
Klimmek (2010)	USA Private health system	Clinical Journal of Oncology Nursing	to use a secondary analysis of data to explore the insurance-related and financial challenges reported by women enrolled in a Managed Care Organization during and after treatment for breast cancer	qualitative (longitudinal)	teaching hospitals and community cancer centers in the southern United States	≥18 years, capable of comprehending written and spoken English, and enrolled in a managed care organization for at least one year	··	purposive	··	semi-structured interview (multiple)	in presence	Phenomenological hermeneutic analysis	interacting with managed care organizations, understanding written information, obtaining authorizations, paying bills and planning for the costs of care, difficulty obtaining assistance with insurance-related tasks
Darby (2009)	USA Private health system	Journal of Health Care for the Poor and Underserved	to explore the financial burden of breast cancer on African American medically underserved women, with the ultimate aim of developing a culturally sensitive understanding about the social and economic stressors minority women face when diagnosed with breast cancer	qualitative	Cancer support and treatment programs across Tennessee and flyers at various treatment centers and community organizations	African American women who were BC survivors	··	··	··	focus group	··	grounded theory	lack of insurance or inadequate insurance resulted in missed, delayed, or fewer treatment opportunities; financial burden was not limited to the acute treatment phase; economic hardship resulting from this disease into long-term survivorship
Lauzier (2005)	Canada National health system	Psycho-Oncology	to describe and better understand how the woman and her caregiver conceptualized their experience of costs from breast cancer	qualitative	through health professionals in the three participating treatment centers of Montreal, Quebec City and Baie-Comeau	newly diagnosed with non-metastatic BC in the 18 months prior to the focus group study	··	purposive	··	focus group	in presence	thematic content analysis	patients' and caregivers' conceptualization of costs; sources of costs

Table 2 reports the data on 1080 participants included in this systematic review. Fifteen studies described the ethnicity of 766 respondents (75.8 %): between 52.5 % [47,48] and 94.0 % of participants [60] were non-Hispanic White/Caucasian patients, while between 4.3 % [56] and 43.0 % of participants [57,59] were non-Hispanic Black patients. Other ethnicities represented were Latina/Hispanic [45,49,54,59], Chinese [41], Malay [41], Indian [41], Asian [49,53], and Pacific Islander [54].

**Table 2:** Participants’ characteristics.

*First author*	*Participants*	*Age mean (SD), median (range), (%), (n)*	*Ethnicity*	*Education Level*	*Children*	*Marital status*	*Income*	*Stage of disease*	*Years since diagnosis*	*Treatments*	*Employment status*	*Insurance type*
Ko (2025)	21	50·7	Hispanic/Latina = 100·0 %	less than high school = 52·4 %	··	unmarried = 40·9 % married/committed relationship = 57·1 %	$25,000 or less = 57·1 % $25,000 to $40,000 = 23·8 % $40,000 or more = 19·1 %	1 = 23·8 % 2 = 33·3 % 3 = 23·8 % 4 = 9·5 % don't know = 9·5 %	within 1 = 23·8 % 1-3 = 47·6 % 3-5 = 28·6 %	S = 95·0 % RT = 47·6 % CT = 61·9 %	··	Medi-Cal = 76·2 % Medicare = 19·0 % Private = 19·0 % Other = 9·5 %
Marshall (2025)	16	55·6 (mean)	White = 88·0 % non-Hispanic = 94·0 %	bachelor's degree or higher = 82·0 %	··	never married = 6·0 % married = 75·0 % divorced = 13·0 % widowed = 6·0 %	<50,000 = 19·0 %	··	··	··	··	··
Do (2024)	33	46 (mean) 26–62 (range)	··	primary school = 36·4 % lower secondary = 21·2 % upper secondary = 9·1 % vocational training = 3·0 % college/university = 30·3 %	··	unmarried = 15·2 % married = 72·7 % divorced = 12·1 %	··	I = 21·2 % II = 45·5 % III = 9·1 % IV = 3·0 % unknown = 24·2 %	··	MT = 90·9 % CT = 69·7 % RT = 42·4 %	··	··
Jones (2024)	18	57·0 (mean)	non-Hispanic White patients = 83·0 %	high school = 100·0 % master's degree/doctorate = 72·2 %	··	married/long-term relationship = 67·0 %	··	I-III = 100·0 % IV = 0·0 %	from 3 to 24	S = 100·0 % CT = 72·0 % RT = 67·0 %	··	··
Khajoei (2024)	16	30–50 years = 50·0 %	··	higher = 60·0 %	··	married = 64·0 %	··	··	··	··	··	··
Patra (2024)	8 *(participants characteristic referred to*80 BC *survivors who attended the quantitative phase)*	48 (40·5–56·5)	··	primary = 3·7 % secondary = 18.7 % college = 77·5 %	··	Married = 83·7 % Unmarried = 5·0 % Divorced/separated/widowed = 11·3 %	Median Rupees = 40,000	metastatic = 23·7 % Non-metastatic = 76·2 %	>5 = 21·2 % ≤5 = 78·7 %	··	employed = 48·7 % housewife = 51·2 %	yes = 33·7 % no = 66·2 %
Ruan (2024)	29	35·1 (29–40)	··	middle or vocational school = 10·3 % high school or junior college = 13·8 % bachelor's degree = 51·7 % master degree = 24·1 %	0 children = 37·9 % 1 child = 48·3 % ≥2 children = 13·8 %	married = 79·3 % unmarried = 20·7 %	··	I = 24·1 % II = 41·4 % III = 31·1 % IV = 3·4 %	<1 = 38·0 % 1-3 = 31·0 % >3 = 31·0 %	S = 100·0 % CT = 89·7 % RT = 79·3 % HT = 51·7 % TT = 27·6 % traditional Chinese therapy = 31·0 %	··	··
Waters (2024)	11 9 = patients 2 = caregivers	mean 50·5 (range = 28–65)	non-Hispanic Black patients = 27·3 % non-Hispanic White patients = 72·7 %	··	··	··	··	··	mean of 3·1 years prior to the interview (range = 1–9)	S = 63·6 % CT = 100·0 % RT = 81·8 % HT = 63·6 % other cancer therapies = 45·5 %	··	private insurance = 72·7 % public insurance = 36·4 %
Khazi (2023)	12	47·7 (6·5)	··	illiterate = 8·3 % primary = 16·7 % high school = 50·0 % graduate = 25·0 %	··	separated = 16·7 % married = 75·0 % widow = 8·3 %	··	I = 8·3 % II = 41·7 % III = 50·0 %	··	··	housewife = 83·3 % accountant = 8·3 % bank employee = 8·3 %	··
Kolawole (2023)	21	31-40 = 28·6 % 41-50 = 28·6 % 51-60 = 4·8 % 61-70 = 38·1 %	··	non-formal = 9·5 % primary = 19·0 % secondary = 28·6 % tertiary = 42·9 %	··	single = 4·8 % married = 81 % widow = 14·2 %	··	··	··	··	full housewife = 4·8 % civil servant = 42·0 % artisan = 4·8 % unemployed = 0·0 % private organization = 4·8 % others = 42·9 %	··
Lee (2024)	50	49·6 (11, range 31–77) ≤40 years = 40 %	Black = 34·0 % White = 66·0 %	··	··	··	··	··	··	··	··	private = 66·0 % Medicaid = 14·0 % Medicare = 14·0 % other/self-pay = 6·0 %
Neilson (2023)	68	<45 years = 27·9 % 45-62 = 57·4 % >62 = 14·7 %	White or Caucasian = 89·7 % Hispanic = 1·5 % black or African American = 4·4 % pacific islander = 2·9 % other = 1·5 %	never received high school diploma = 1·5 % high school diploma = 4·4 % trade/technical/vocational training = 2·9 % some college level credits or associates degree = 22·1 % bachelor's degree = 36·8 % master's degree = 25·0 % PhD or other advanced degree = 7·4 %	none = 29·4 % ≤2 = 58·8 % >2 = 11·8 %	single = 17·7 % married = 60·3 % divorced = 8·8 % in relationship = 4·4 % separated = 2·9 % widowed = 5·9 %	≤ $30,000 = 10·3 % $30,001-$60,000 = 22·1 % $60,001-$90,000 = 20·6 % >$90,000 = 41·1 % no response = 5·9 %	··	··	··	employed = 66·2 % disabled = 4·4 % retired = 14·7 % not employed/seeking work = 10·3 % not employed/not seeking work = 4·4 %	private = 70·6 % Medicare = 8·8 % Medicaid = 8·8 % self-Pay = 5·9 % none = 1·5 % other = 4·4 %
Walton (2023)	14	57·1 (mean)	··	··	··	··	··	··	··	S = 100·0 % S, CT and RT = 78·6 %	homemakers = 78·6 %	··
Aitken (2022)	15	Mean 45 35-39 = 26·6 % 40-45 = 20,0 % 46-49 = 6·6 % 50-55 = 13·3 % 56-60 = 13·3 %	··	high school = 26·6 % diploma = 26·6 % graduate certificate = 6·7 % undergraduate = 13·3 % postgraduate = 26·6 %	0 child = 6·7 % 1-2 = 53·3 % 3-4 = 33·3 %	married = 80·0 % divorced = 6·7 % single = 6·7 % de-facto = 6·7 %	20,000–50,000 = 13·3 % 50,000–100,000 = 26·6 % 100,000–150,000 = 6·7 % 150,000–200,000 = 26·6 % >200,000 = 20 %	··	··	··	employed = 66·0 % unemployed = 33·3 % full-time = 20·0 % part-time/casual = 46·0 %	··
Marshall (2022)	16	55·6 (mean)	Caucasian = 88·0 % Black/African American = 6·0 % Asian = 6·0 %	high school diploma = 6·0 % associate degree = 12·0 % bachelor's degree = 38·0 % graduate or professional degree = 44·0 %	··	never married = 6·0 % married = 75·0 % divorced = 13·0 % widowed = 6·0 %	< $24,999 = 12·5 % $25,000 to 49,999 = 6 % $50,000 to 99,999 = 38·0 % $100,000 to 149,999 = 25·0 % ≥ $150,000 = 12·5 % refused = 6·0 %	··	1–3 years = 37·5 % 4–9 = 31·0 % 10–15 = 19 % 15 = 12·5 %	Talazoparib = 12·5 % Fulvestrant = 19·0 % Denosumab = 19·0 % Paclitaxel = 31·0 % Carboplatin = 12·5 % Abemaciclib = 6·0 % Letrozole = 12·5 % Palbociclib = 19·0 % Pertuzumab = 12·5 % Trastuzumab = 12·5 % Exemestane = 6·0 % Sacituzumab = 6·0 % Gemcitabine = 6·0 % Vinorelbine = 6·0 % Capecitabine = 6·0 %	currently employed = 38·0 % not employed = 62·0 %	··
Prabandari (2022)	20	40-49 = 50·0 % 50-59 = 30·0 % 60-69 = 20·0 %	··	primary/junior high school = 70·0 % senior high school/university = 30·0 %	0 = 5·0 % 1 = 15·0 % 2 = 60·0 % ≥3 = 20·0 %	single = 5·0 % widowed = 5·0 % married = 90·0 %	··	III = 15·0 % IV = 85·0 %	0 year = 30·0 % 1 = 60·0 % 3 = 10·0 %	··	housewife = 50·0 % employed/self-employed = 45·0 % unemployed = 5·0 %	··
Agha (2021)	42	18–37 years = 31·0 % 38-52 = 52·4 % 53-62 = 16·7 %	··	none = 23·8 % primary = 26·2 % secondary = 11·9 % higher secondary = 14·3 % graduate = 23·8 %	··	··	··	··	··	··	··	··
Gharzai (2021)	32	45·9 (11·8)	White = 58·1 % Black = 25·8 % Hispanic = 9·7 % Asian = 6·5 % not reported = 3·1 %	less than bachelor's degree = 34·3 % bachelor's degree = 56·3 % more than bachelor's degree = 9·4 %	·· 53·1 % had persons who required financial support	·· 53·1 % were sole breadwinner	< $25,000 = 25·0 % $25,000-$50,000 = 31·3 % $50,000-$75,000 = 21·9 % > $75,000 = 21·9 %	··	··	··	full-time = 3·1 % part-time = 18·8 % self-employed = 3·1 % disability = 25·0 % FMLA or sick leave = 31·3 % unemployment = 18·8 %	employer = 34·4 % Medicaid or Medicare = 12·5 % private = 3·1 % not reported = 50·0 %
Lewis (2021)	38	36–74 (mean 57·3, median 57·5)	most participants were Australian born and of European decent	participants across a variety of educational attainment	··	··	··	IV = 100 %	participants across a variety of length of time since diagnosis (<1–23 years)	··	··	private health insurance = 47·0 % public health system insurance = 37·0 % mixed public and private health insurances = 13·0 %
Oshima (2021)	511 answered to the survey· (313 respondents wrote about financial burden)	50·0 (26·1–76·4)	463 White and/or Caucasian (90·6 %) 22 Black and/or African American (4·3 %) 23 Other or more than one race (4·5 %) 3 Prefer not to answer (0·6 %)	111 = Up to an Associate's degree (21·7 %) 159 = College graduate (4 y) (31·1 %) 241 = Graduate education (47·2 %)	··	376 = Married or living with partner (73·6 %) 133 = Widowed, divorced, or single (26·3 %) 2 = Prefer not to answer (0·4 %)	157 = Up to $74,000 (30·7 %) 151 = between $74,001-$125,000 (29·6 %) 140 = over $125,000 (27·4 %) 63 = Prefer not to answer (12·3 %)	0 = 83 (16·2 %) 1 = 189 (37·0 %) 2 = 153 (29·9 %) 3 = 55 (10·8 %) 4 = 9 (1·8 %) 22 = Unknown (4·3 %)	346 = <10 y (67·7 %) 146 = ≥10 y (30·5 %) 9 = Missing (1·8 %)	S = 95·0 % CT = 60·7 % RT = 62·0 %	Current 280 = Employed full- or part-time (54·8 %) 230 = Not employed for pay (includes students and retirees) (45·0 %) 1 = Prefer not to answer (0·2 %)	343 = Private (67·1 %) 142 = Medicare (27·8 %) 26 = Other insurance (5·1 %)
Chebli (2020)	19	18–50 years = 22·0 % ≥51 = 78·0 %	19 = Latina (100 %·0)	high school = 47·0 % high school or more = 47·0 % missing = 5·0 %	··	married = 63·0 % not married = 37·0 %	< $30,000 = 53·0 % $30,000 or more = 32·0 % missing = 15·0 %	··	0–2 years = 32·0 % 3–5 = 53·0 % 6–11 = 10·0 % missing = 5·0 %	S = 100·0 % also CT only = 5·0 % also RT only = 17·0 % also HT only = 28·0 % combination of therapies besides S = 39·0 %	··	insured = 67·0 % not insured = 33·0 %
Iddrisu (2020)	12	35·9 (5·6, range 32–45)	··	junior high school = 16·7 % senior high school = 25·0 % tertiary = 58·3 %	0 children = 16·6 % 1 child = 41·6 % ≥2 children = 41·6 %	married = 50·0 % single = 41·6 % divorced = 8·3 %	··	··	7–48 months	MT = 50·0 % CT = 66·7 % HT = 25·0 % RT = 8·3 %	employed = 100·0 %	··
Kong (2020)	64	49·0 (median) <40 years = 17·2 % 40–59 = 75·0 % ≥60 = 7·8 %	Malay = 40·6 % Chinese = 45·3 % Indian = 14·1 %	primary = 7·8 % secondary = 51·6 % tertiary = 40·6 %	··	single = 6·3 % married = 79·7 % other = 14·1 %	< $1045 per month = 51·6 % $1045–$2306 per month = 26·6 % > $2306 per month = 21·9 %	··	at least 1 year (eligibility criteria)	··	··	self-pay/private = 58·7 % employer = 67·4 % none = 28·1 %
Dean (2019) SCC	40	64 (8)	17 = Black (42·5 %) 2 = Other (5·0 %) 21 = White (52·5 %)	19 = high school (47·5 %) 12 = college (30·0 %) 9 = graduate school (22·5 %)	··	··	4 = ≤$30,000 (10·5 %) 22 = $30,001-$70,000 (57·8 %) 12= >$70,000 (31·6 %)	10 = 0 (32·3 %) 9 = 1 (29·0 %) 7 = 2 (22·6 %) 5 = 3 (16·1 %) 9 = missing (22·5 %)	Mean 12 years (SD ± 5)	RT = 82·5 % CT = 76·9 % HT = 25·0 % lymphedema, +BCRL = 60·0 %	··	12 = public (30·0 %) 33 = private (82·5 %) 0 = none (0·0 %)
Dean (2019) Cancer	40	64 (8)	17 = Black (42·5 %) 2 = Other (5·0 %) 21 = White (52·5 %)	19 = high school (47·5 %) 12 = college (30·0 %) 9 = graduate school (22·5 %)	··	··	4 = ≤$30,000 (10·5 %) 22 = $30,001-$70,000 (57·8 %) 12= >$70,000 (31·6 %)	10 = 0 (32·3 %) 9 = 1 (29·0 %) 7 = 2 (22·6 %) 5 = 3 (16·1 %) 9 = missing (22·5 %)	Mean 12 years (SD ± 5)	RT = 82·5 % CT = 76·9 % HT = 25·0 % lymphedema, +BCRL = 60·0 %	··	12 = public (30·0 %) 33 = private (82·5 %) 0 = none (0·0 %)
Japhet (2019)	22	50·5 (15·8)	··	none = 54·5 % primary = 18·2 % secondary = 18·2 % tertiary = 9·1 %	··	··	··	II = 9·1 % III = 9·1 IV = 81·8 %	1 year = 13·6 % 2 = 36·4 % 3 = 9·1 % 4 = 18·2 % 5 = 22·7 %	··	··	··
Nolan (2019)	15	28-42 (mean 35·0)	··	some college = 16·0 % bachelor's degree = 60·0 % graduate degree = 27·0 %	at least one = 73·0 %	single/divorced = 60·0 % married/partnered = 40·0 %	<30 K = 33·0 % 30,001–50 K = 33·0 % 50,001–70 K = 27·0 % >70 K = 7·0 %	··	·· On average, participants completed active treatment 3·2 years prior to the time of study	S = 100·0 % CT = 73·0 % RT = 80·0 % HT = 27·0 %	disabled/unemployed = 33·0 % employed part-time = 7·0 % employed full-time = 60·0 %	··
Pisu (2019)	42	60–64 years = 71·0 % 65-69 = 14·0 % ≥70 = 14·0 %	African American = 43·0 % White = 57·0 %	high school = 17·0 % 1–3 years' college = 40·0 % ≥4 years' college = 43·0 %	··	··	<$25 000 = 33·0 % $25 000 to <$50 000 = 12·0 % $50 000 to <$75 000 = 29·0 % ≥$75 000 = 29·0 % missing = 5·0 %	··	··	CT = 45·0 % RT = 59·0 % HT = 21·0 %	··	Medicare = 17·0 % Commercial/private insurance = 67·0 % other = 9·0 % no insurance = 7·0 %
McEwan (2014)	15	range 29–60	··	university degree = 26·6 % high school = 33·3 %	at least one = 100·0 %	married = 80·0 %	··	··	··	··	employed = 33·3 % (5 were on sick leave at the time of interview) unemployed = 66·6 %	··
Klimmek (2010)	14	52·9 (mean)	predominantly White	high school education = 50·0 % college education = 50·0 %	··	predominantly not married	predominantly middle/high socioeconomic status	··	··	3 interviews were conducted at diagnosis or pretreatment 6 were conducted during treatment 13 were conducted in the follow-up period	predominantly employed either full- or part-time	··
Darby (2009)	36	50·0 (11·4) 31–45 years = 22·0 % 46-60 = 29·0 % >60 = 50·0 %	··	··	··	··	··	··	··	··	··	··
Lauzier (2005)	26	53·0 (mean, 28–78)	··	··	··	··	annual gross family income $10 000–19 999 = 11·5 % $20 000–29 999 = 15·4 % $30 000–39 999 = 11·5 % $40 000–49 999 = 19·2 % >$50 000 = 42·3 %	I-III = 100·0 % IV = 0·0 %	10 months (4–16)	MT = 80·8 % conservative S = 19·2 % AD = 96·1 % CT = 61·5 % RT = 76·9 % HT = 80·8 %	no = 26·9 % employed = 61·5 % self-employed = 11·5 %	··

S = surgery; MT = mastectomy; AD = axillary dissection; CT = chemotherapy; RT = radiotherapy; HT = hormone therapy; TT = targeted therapy.

Fifteen studies described the employments status of 601 respondents (55.6 %): employed participants were between 33.3 % [34] and 100.0 % [35,43]; homemakers were between 4.8 % [43] and 83.3 % [36]. Thirteen studies described the insurance type of 704 respondents (65.2 %): between 3.1 % [49] and 82.5 % of participants [47,48] had a private insurance, while between 30.0 % [47,48] and 37.0 % of participants [31] had a public insurance. Another type of insurance was Medicaid/Medicare (USA), reported by between 8.8 % [54] and 76.2 % of participants [59].

Fifteen studies described the treatment modality of 621 respondents (57.5 %): between 63.6 % [58] and 100 % of participants [33,37,45,50,55] underwent surgery, from 45.0 % [57] to 100.0 % of participants [58] underwent chemotherapy, from 21.0 % [57] to 80.8 % [32] did hormone therapy, and from 8.3 % [35] to 82.5 % [47,48] underwent radiotherapy.

### 3.1 Appraisal consideration

Appendix D reports the results of the quality assessment (p 7); all the reports clearly stated their research aims. In almost all studies, the appropriateness of the study design, recruitment strategy, and data collection methods were positively evaluated by both researchers. However, the assessment of the relationship between researchers and participants was generally lacking, as most studies did not provide sufficient detail. Ethical considerations were generally well addressed, although some studies lacked details on informed consent. Data analyses were often rated as “Can't tell” due to insufficient descriptions of the analytical process. Nevertheless, most studies explicitly and clearly presented their findings, and over half reported adequate level of generalizability.

### 3.2 Meta-synthesis findings

**Fig. 2:**
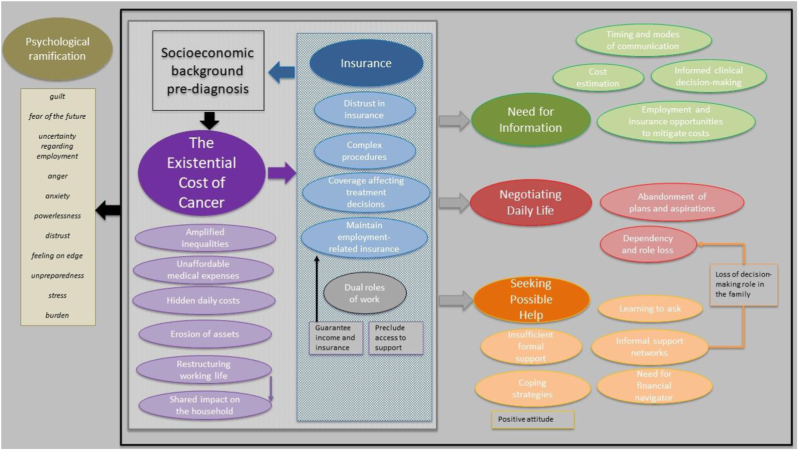
Interpretative model of cancer-related FT.

The experience of individuals with BC concerning their cancer-related FT has been described through five main themes and related subthemes: (i) the existential cost of cancer (amplified inequalities, unaffordable medical expenses, hidden daily costs, erosion of assets, restructuring working life, shared impact on the household, work as anchor or obstacle), (ii) impact of insurance complexity (complex procedures, coverage affecting treatment decisions, distrust in insurance, maintain employment-related insurance), (iii) need for timely and accessible information (informed clinical decision-making, timing and modes of communication, cost estimation, employment and insurance opportunities to mitigate costs), (iv) seeking possible help (coping strategies, informal support networks, need for financial navigator, insufficient formal support, learning to ask), (v) negotiating daily life (dependency and role loss, abandonment of plans and aspirations). We interpreted the five themes in relation to each other by building an interpretative model, which is reported in Fig. 2.

### 3.3 The existential cost of cancer

BC diagnosis interfered in the lives of individuals across different socioeconomic backgrounds, amplifying pre-existing inequalities through sustained, unaffordable, and often perceived as unfair medical costs [54]. One of the main consequences was the erosion of personal assets and life savings [41,47] as the only possible source to cope with these expenses (and therefore to survive cancer), particularly when insurances did not guarantee the coverage of treatments. An individual with BC from the USA (private health system) said [56], *“We had to use savings to cover all of the costs, ask our families for* support*, and constantly deal with various office billing departments”* and a patient from Malaysia (national health system) reported*,* [41] *“I am facing financial difficulties, I have to withdraw my savings to* support *my daily living.”*

Nevertheless, inequalities stemmed not only from medical costs but also from the unexpected daily expenses, like transport and temporary housing for rural patients needing hospital access [52]. In this scenario, employment played a dual role. For some patients, it was the main anchor, the only source of income available to cover expenses, maintain insurance, or compensate for the lack of coverage [54]: *“If you don't work during your treatment, you don't have resources …”* (USA – private health system).

However, the amount of earnings from work may have dwindled due to employment changes after diagnosis, such as extended sickness absences or job loss [52]. Conversely, individuals' pre-diagnosis employment status was sometimes perceived as a barrier to accessing support after diagnosis, especially when individuals were at risk of job loss and had to manage work and cancer treatment [48]: *“I think there are things in place for people who are very financially needy, but not really for someone who is like … I don't know what kind of classification I am, but who can still work …*” (USA – private health system).

One thing was certain: patients restructured their lives (as workers but not only), with repercussions on others, especially on the closest family members. Caregivers adapted their work to take care of assistance and household chores [39]: *“My husband used to work at the airport, but because of taking care of me, he quit his job and is now working nonpermanent jobs.”* (Indonesia – poorly institutionalized system).

The existential costs of cancer trigger emotional turmoil, fear of financial ruin and job loss, guilt toward loved ones, and anger at the disease and society.

### 3.4 Impact of insurance complexity

Patients' experiences revealed a feeling of distrust in insurance, mainly due to the lack of transparency and dishonesty [50] that leads these organizations to have only one goal: to put pressure on sick patients [46] without caring about their care or privacy [51]. An individual with BC from the USA (private health system) said [50]: *“But when I was going through this I had to go on disability. My disability insurance tried to mess with how much they were giving me. Yes, it was thousands of dollars.”*

Disbelief was exacerbated by delays between prompt treatment and slow insurance processes [51]. Gaps in coverage, co-payments, and reimbursement left patients unprepared, as they lacked both information and financial resources [33,41,46], not to mention those who did not have insurance coverage, like a Malaysian patient (national health system) who stated [41], *“Mastectomy and prosthesis comes to US$ 241, which I am quite mindful. If the prosthesis is cheaper, then I will pay for it. But since it is over US$ 241, I am willing to forego it because of costs.”*

Continuing to work was often seen as the only way to retain insurance coverage, though it was not guaranteed, as it largely depended on the type of employment contract [32].

Challenging relationships and widespread distrust towards insurers led patients to make treatment choices based on coverage and financial constraints [46], reinforcing feelings of powerlessness and injustice.

### 3.5 Need for timely and accessible information

Patients felt overwhelmed and unprepared to face cancer's financial impact, reporting poor access to clear, timely information on direct and indirect costs [54]. An individual with BC from the USA (private health system) reported [49]: *“I think that's the hardest thing for me was the stress and the burden of the financial hardship that you go through. You feel like it's never going to end.”* Another patient from India (poorly institutionalized system) said [38]: *“There is no information on the entire treatment; it's like you go for surgery, then they give you the estimate, then your next step is chemotherapy, then they give an estimate for chemotherapy, then similar for radiation.”*

Unmet information needs caused psychological distress, including the lack of guidance regarding the consequences of work changes and the role of income in managing care and maintaining insurance [50].

Individuals with BC from the USA (private health system) acknowledge the need for timely information provision, although the optimal timing remains unclear, whether it should be in conjunction with the diagnosis (*“Right from the very beginning, absolutely, as soon as you're diagnosed and you go in there”),* [49] *or not (“I think cancer patients get bombarded with information in the beginning”),* [54] or after diagnosis but before starting treatment [57].

Patients appropriately informed on the cost-related aspects of disease were competent to make informed clinical decisions and to deal with the disease with higher self-efficacy, as reported by a Chinese patient (national health system) [33]: *“After illness, I read the literature by myself, then discussed it with the doctor for the optimal treatment method, and adhered to it. I knew how to treat it and understood my own condition.”* To improve their situation, individuals with BC who lacked information sought available support.

### 3.6 Seeking possible help

Despite the challenges, BC survivors engaged in various coping strategies to manage FT. One of the first resources they activated was maintaining a positive attitude, which is perceived as essential to navigating the financial uncertainty caused by the illness, as reported by individuals with BC from different countries: *“On some days I worry … God, what am I supposed to be doing? But at the end of the day I'm optimistic”* [55] (USA - private health system) and *“There is not much economic pressure. I am quite confident. My illness will definitely get better in the future.”* [33] (China - national health system)

Coping strategies also included a careful rebalancing of family needs according to financial priorities and prognosis [33]: *“If he (son) wants something that is not related to learning, I will consider whether to buy it, unlike before when he could buy whatever he wanted … As for myself, now I basically don't buy luxury goods.”* (China – national health system).

Not least, survivors must sometimes face the scenario of an unfavourable prognosis and the potential financial impact that a premature death could impose on vulnerable family members. In this context, a reorganization of family dynamics and a redefinition of roles often emerge, shaped by the need to face both financial and clinical uncertainties because, *“It's like, okay, so if I have these two years left, I need to pay whatever I can to live. But on the flip side, I don't want to go on any vacations, I don't want to buy anything extra. I want to pay down as much debt as we possibly can. Because if things go sideways, I've got to leave my family in a good situation. So, it's a real razor wire that you're walking, for cancer treatment, I think.”* [52] (USA – private health system).

A key aspect of coping involved learning to seek help. Survivors may turn to family, friends, and colleagues, overcoming the discomfort that admitting vulnerability can bring and building informal networks of resilience that offer both emotional and financial support [37,50], as reported by individuals with BC from different countries: “O*ne of my friends sent a message to the classmates group, one friend said I will pay your bill, I managed with the money sent by friends.”* [37] (India - poorly institutionalized system) and *“We had to ask for help a lot. They did a fundraiser for me. That helped us get through a bit. Yeah, people brought us groceries and food. Having that community around to help is what enabled us to take care of those other basic needs.”* [49] (USA - private health system)

Requesting assistance is also directed toward formal institutions, which requires learning to interact with administrative systems and unfamiliar procedures, often at a time when energy and attention should be focused on healing [58]: *“I need help with my electricity bill. It was kind of like, well, we need proof. They said, we're sure you're telling the truth, but we need proof from your doctor that you have cancer. So, I had to leave there, go all the way down to the hospital … wait for it … and then come all the way back and get assistance. And, if you don't get it by a certain day, you miss it that month.”* (USA – private health system).

Many survivors advocate for earlier and more structured communication about care costs, and describe support from dedicated professionals, such as case managers or financial navigators [48,58], as valuable in navigating resources, completing forms, and managing bureaucratic processes [45]: *“I brought up the cost with every doctor I met and every time I had an appointment. My oncologist finally told me not to mention it again as she did not want my financial situation to affect her medical decisions!”* (USA – private health system).

Resilience is further strengthened through broader support networks, which may include not only personal relationships but also community services and the work environment [58]: *“I received things that I didn't even ask about … [social worker] signed me up for things that I wasn't even aware of. You know, so it was great to open the mail, and get a gift card for [groceries].”* (USA – private health system).

The workplace, in particular, can serve as a crucial source of support in mitigating FT, offering flexibility, understanding, and protections that may significantly help BC survivors manage the economic challenges associated with their illness [33]: “*No change in position, no change in salary. The workload is relatively small now; I only need to manage my subordinates, and then I can get off work when the time comes. In the past, I had to work overtime for a long time.”* (China – national health system). Even though the stress may be unbearable in certain situation [35], *“I get stressed up and stress is not good so I had to slow down for a while. I finally withdrew from work totally.”* (Ghana – poorly institutionalized system).

### 3.7 Negotiating daily life

Rising costs and limited resources forced lifestyle changes and gradual loss of access to essential and non-essential goods because *“I am trying to make sure that I have the money that I need to go to the doctor.”* [58] (USA – private health system) Negotiation meant suffering as *“I had to miss life, things with my kids and events*.*”* [54] (USA – private health system) Deprivation did not only concern the present but also the abandonment of plans for the personal and family future. Individuals with BC from the USA and China reported: *“I owe so much in medical bills that I have decided not to purchase a home”* [52] (USA – private health system) and *“I worry about my daughter's future education and the future expenses of our family.”* [33] (China – national health system) Debts and difficulties in recovering lost money create a strong economic dependence on family members and the consequent loss of role. Accessing informal financial support may be experienced with discomfort, including a perceived loss of economic independence and decision-making role within the family.

## 4 Discussion

This study interprets the lived experience of cancer-related FT among individuals with BC. Most of the included studies were conducted in North America, with additional research originating in South, East, and West Asia, Oceania, and Northwest Africa. Currently, there are no qualitative studies from European countries addressing cancer-related FT in individuals with BC, despite evidence of FT in high-income nations, including nearly all European countries [19]. The findings of this review underscore the transversal nature of key dimensions of cancer-related FT in individuals with BC across healthcare systems, showing that, despite structural differences, socioeconomic and cultural conditions consistently shape patients’ experiences of financial burden.

Corroborated by a recent scoping review [62], BC survivors who lived in rural areas and with lower socioeconomic status faced greater challenges in affording medical costs, including surgery or chemotherapy. An individual with BC in the USA (private health system) was forced to sell her car [54], while a Nigerian patient (poorly institutionalized system) had to sell her goat to cover medical expenses [42].

Additionally, hidden non-medical costs, such as those incurred when traveling to the most economically accessible hospital, posed a real burden on patients and families. Due to prolonged wait times within the Canadian healthcare system, a patient was compelled to seek radiation therapy treatment abroad [32]. Therefore, a patient's socioeconomic status at the time of diagnosis, as well as any changes resulting from the cancer, such as job loss, can significantly shape their experience of FT.

As medical and non-medical expenses increased, employment was often perceived as a bulwark against FT. While employment is typically assumed to ensure adequate income for daily living, savings, and insurance coverage, cancer-related challenges, such as job loss or reduced work capacity, can severely undermine an individual's financial stability. In these circumstances, individuals with BC frequently relied on personal savings to cover treatment costs or turned to alternative sources of support. This reliance on personal financial resources is particularly common in healthcare systems where patients face substantial out-of-pocket expenses due to limited welfare support [49,56].

This scenario was also reported by several Egyptian BC survivors [63] and by caregivers of Indian cancer survivors [64]. Findings from this meta-synthesis reveal that using savings to cover treatment costs was necessary but heartbreaking, as it disrupted future plans [54]. Conversely, being employed at the time of diagnosis may be disadvantageous in accessing formal support after diagnosis. In certain circumstances, individuals with BC who were already unemployed or retired at the time of diagnosis appeared to experience lower levels of FT compared to those who became unemployed because of their illness [62].

Insurance coverage played a pivotal role in shaping the lived experience of cancer-related FT. Findings from this meta-synthesis support the notion that insurance policies often influence patients' treatment decisions, leading them to consider their financial situation and coverage limitations when selecting care options. While full coverage from insurance companies appeared to protect against FT [7], several individuals only had partial coverage and thus needed co-financing, and others had no insurance coverage at all. Insurance coverage reflects the patient's socioeconomic status, exacerbating inequality in cancer care costs. Half of the included studies were mainly conducted in the USA, a country where two-thirds of the health system is based on voluntary insurance, while the government-funded support programs are for the most vulnerable subjects (ie, the elderly) [65]. Therefore, insurance coverage depends on a patient's pre-diagnosis socioeconomic status. Overall, having insurance and clear information helped patients make informed clinical decisions. However, even those who had insurance coverage reported distrust, as they felt cheated, or because the procedures were complex at a time when promptness is required [60].

To address the unexpected costs of cancer, individuals with BC requested clear cost estimates and guidance on mitigating expenses through employment and insurance. Furthermore, patient preference regarding the timing of receiving cost-related information varied, emphasizing that a standardized approach is inadequate [66]. To face the cost of cancer, individuals with BC requested and received support from family members, the workplace, social network, and the community, sometimes even through fund raising [33]. Nonetheless, they felt uncomfortable asking for help, showing their vulnerability, and interfering with other people's lives. As a matter of fact, family members of cancer survivors suffered the most the economic repercussions of cancer treatment [67]. This meta-synthesis highlighted that sometimes the informal caregiver was forced to change his/her work condition in order to assist with domestic responsibilities [39]. This life rebuilding had an impact on household incomes. Therefore, an emerging need was to receive specific support beyond the family, specifically from a manager of the economic aspects of cancer care.

FT affected both patients and their families, forcing lifestyle changes to align costs with limited income and resources. Abandoning future plans and reducing spending on essential and non-essential goods were common changes reported [52], consistent with findings from a systematic review of Indian cancer survivors [68]. Individuals with BC faced the painful choice of treatment based on affordability, leading to emotional distress, a diminished sense of autonomy in decision-making, distrust in institutions, and feelings of guilt and of being a burden.

To summarize, this meta-synthesis is the first systematic review of qualitative studies examining the broad experience of cancer-related FT from the perspective of individuals with BC across all phases of the treatment pathway. Unlike previous reviews, which focused solely on coping strategies [20], this study included global research involving participants at varying disease stages, survivorship phases, and with different ethnic background, with the aim of capturing diverse and comprehensive experiences of FT. By not restricting inclusion based on healthcare systems, the review also considered different social contexts. Although the extensive number of studies may have limited the ability to capture certain nuanced meanings, the complete database remains open for consultation.

The review reveals a key gap: a lack of original qualitative research in Europe. This is crucial, as the experience of cancer-related FT is shaped not only by treatment costs but also by country-dependent healthcare system and cultural nuances. Europe's diverse healthcare systems may shape different FT perceptions, warranting further study.

An additional insight from this meta-synthesis is the multifaceted role of employment. Work can either help or hinder individuals with BC in managing FT, affecting both pre-diagnosis socioeconomic status and insurance coverage. Insurance, in turn, influences the existential cost of illness and is tightly linked to each country's healthcare systems.

Although this meta-synthesis focused on shared experiential dimensions of cancer-related FT, studies were conducted within diverse healthcare systems. As participants may not explicitly articulate macro-structural influences on their care trajectories, contextual effects were inferred. Therefore, healthcare systems were grouped according to dominant financing and coverage mechanisms. In predominantly private health systems, participants’ narratives emphasized that FT was shaped by insurance complexity, coverage gaps, and employment-linked benefits [46,50,51,54,56]. In national health systems, it was more commonly associated with indirect and non-covered costs, income loss, and administrative burdens [[30], [31], [32],^41^]. In under-resourced or poorly institutionalized contexts, FT was embedded in broader social insecurity and often experienced as an existential threat affecting both individuals and their families [35,[37], [38], [39]]. Although not intended as a systematic cross-country comparison, this framing illustrates how structural conditions influence the lived experience of FT from a patient-centred perspective. Therefore, the subthemes identified should be adapted based on each country. This context dependency limits the generalizability of the findings, and results should be interpreted with appropriate caution.

To conclude, this meta-synthesis enhances our understanding of the experience of cancer-related FT among individuals with BC, particularly its impact on daily life. The interpretative model can guide healthcare and social professionals in the screening of FT by mapping the problems encountered to promote communication that includes the economic implications of cancer diagnosis. These steps may positively affect clinical decision-making and patient's adherence to treatment as well as mitigate the psychological consequences associated with FT [69].

## CRediT authorship contribution statement

**Sara Paltrinieri:** Writing – review & editing, Writing – original draft, Visualization, Project administration, Methodology, Investigation, Formal analysis, Data curation, Conceptualization. **Margherita Schiavi:** Writing – review & editing, Writing – original draft, Visualization, Project administration, Methodology, Investigation, Formal analysis, Data curation, Conceptualization. **Barbara Bressi:** Writing – review & editing, Investigation, Formal analysis, Data curation. **Angela Contri:** Writing – review & editing, Investigation, Formal analysis. **Martina Torreggiani:** Writing – review & editing, Investigation, Data curation. **Laura Bernardi:** Writing – review & editing, Investigation, Data curation. **Elisa Mazzini:** Writing – review & editing, Investigation, Conceptualization. **Maria Chiara Bassi:** Writing – review & editing, Methodology, Investigation, Conceptualization. **Silvia di Leo:** Writing – review & editing, Methodology, Investigation, Conceptualization. **Luca Ghirotto:** Writing – review & editing, Methodology, Investigation, Conceptualization. **Stefania Costi:** Writing – review & editing, Writing – original draft, Supervision, Project administration, Methodology, Investigation, Formal analysis, Data curation, Conceptualization.

## Data sharing statement

Dataset reporting first order and second order constructs extracted from each original qualitative study included in this meta-synthesis are available for consultation in an online repository (https://doi.org/10.5281/zenodo.16561567).

## Funding

This study was partially supported by the Italian Ministry of Health-Ricerca Corrente Annual Program 2027.

## Declaration of competing interest

The authors declare that they have no known competing financial interests or personal relationships that could have appeared to influence the work reported in this paper.
