# Supplementary material for: “You're really walking along the razor's edge”: A meta-synthesis on the existential cost of breast cancer related to financial toxicity
Source: Breast. 2026 Jan 20;86:104705. doi: 10.1016/j.breast.2026.104705 (PMC12860959; doi:10.1016/j.breast.2026.104705)
Supplement: Multimedia component 1 [file mmc1.docx]

**Appendix A. ENTREQ guidelines for reporting**

| **No** | **Item** | **Guide and description** | **Page** |
| --- | --- | --- | --- |
| 1 | Aim | State the research question the synthesis addresses | 2-3 |
| 2 | Synthesis methodology | Identify the synthesis methodology or theoretical framework which underpins the synthesis, and describe the rationale for choice of methodology (e.g. meta-ethnography, thematic synthesis, critical interpretive synthesis, grounded theory synthesis, realist synthesis, meta-aggregation, meta-study, framework synthesis). | 4 |
| 3 | Approach to searching | Indicate whether the search was pre-planned (comprehensive search strategies to seek all available studies) or iterative (to seek all available concepts until they theoretical saturation is achieved). | 3 |
| 4 | Inclusion criteria | Specify the inclusion/exclusion criteria (e.g. in terms of population, language, year limits, type of publication, study type). | 3 |
| 5 | Data sources | Describe the information sources used (e.g. electronic databases (MEDLINE, EMBASE, CINAHL, psycINFO, Econlit), grey literature databases (digital thesis, policy reports), relevant organisational websites, experts, information specialists, generic web searches (Google Scholar) hand searching, reference lists) and when the searches conducted; provide the rationale for using the data sources. | 3 |
| 6 | Electronic Search strategy | Describe the literature search (e.g. provide electronic search strategies with population terms, clinical or health topic terms, experiential or social phenomena related terms, filters for qualitative research, and search limits). | Appendix B |
| 7 | Study screening methods | Describe the process of study screening and sifting (e.g. title, abstract and full text review, number of independent reviewers who screened studies). | 3,4 |
| 8 | Study characteristics | Present the characteristics of the included studies (e.g. year of publication, country, population, number of participants, data collection, methodology, analysis, research questions). | 4-29, Tables 1 and 2 |
| 9 | Study selection results | Identify the number of studies screened and provide reasons for study exclusion (e,g, for comprehensive searching, provide numbers of studies screened and reasons for exclusion indicated in a figure/flowchart; for iterative searching describe reasons for study exclusion and inclusion based on modifications t the research question and/or contribution to theory development). | Figure 1, Appendix C |
| 10 | Rationale for appraisal | Describe the rationale and approach used to appraise the included studies or selected findings (e.g. assessment of conduct (validity and robustness), assessment of reporting (transparency), assessment of content and utility of the findings). | 3,4 |
| 11 | Appraisal items | State the tools, frameworks and criteria used to appraise the studies or selected findings (e.g. Existing tools: CASP, QARI, COREQ, Mays and Pope [25]; reviewer developed tools; describe the domains assessed: research team, study design, data analysis and interpretations, reporting). | 3,4 |
| 12 | Appraisal process | Indicate whether the appraisal was conducted independently by more than one reviewer and if consensus was required. | 3,4 |
| 13 | Appraisal results | Present results of the quality assessment and indicate which articles, if any, were weighted/excluded based on the assessment and give the rationale. | 29, Appendix D |
| 14 | Data extraction | Indicate which sections of the primary studies were analysed and how were the data extracted from the primary studies? (e.g. all text under the headings “results /conclusions” were extracted electronically and entered into a computer software). | 4 |
| 15 | Software | State the computer software used, if any. | 4 |
| 16 | Number of reviewers | Identify who was involved in coding and analysis. | 4 |
| 17 | Coding | Describe the process for coding of data (e.g. line by line coding to search for concepts). | 4 |
| 18 | Study comparison | Describe how were comparisons made within and across studies (e.g. subsequent studies were coded into pre-existing concepts, and new concepts were created when deemed necessary). | 4 |
| 19 | Derivation of themes | Explain whether the process of deriving the themes or constructs was inductive or deductive. | 4 |
| 20 | Quotations | Provide quotations from the primary studies to illustrate themes/constructs, and identify whether the quotations were participant quotations of the author’s interpretation. | 29-35, database in the repository |
| 21 | Synthesis output | Present rich, compelling and useful results that go beyond a summary of the primary studies (e.g. new interpretation, models of evidence, conceptual models, analytical framework, development of a new theory or construct). | 29-35 |
|  |  |  |  |

**Appendix B. Search strategy**

**MEDLINE**
(((breast AND (cancer OR tumor OR tumour OR neopalsm* OR carcinoma)) OR ("Breast Neoplasms"[Mesh])) AND ("Financial Stress"[MeSH] OR financ*[Title] OR money[Title] OR econom*[Title])) AND ("Qualitative Research"[Mesh] OR "Focus Groups"[Mesh] OR "Grounded Theory"[Mesh] OR "Interviews as Topic"[Mesh] OR "Empirical Research"[Mesh] OR "Behavior Observation Techniques"[Mesh] OR qualitat*[Title/Abstract] OR "qualitative research"[Title/Abstract] OR "qualitative stud*"[Title/Abstract] OR "qualitative design"[Title/Abstract] OR "grounded theory"[Title/Abstract] OR narrat*[Title/Abstract] OR ethno*[Title/Abstract] OR phenomenol*[Title/Abstract] OR "action research"[Title/Abstract] OR "action-research"[Title/Abstract] OR "participatory action-research"[Title/Abstract] OR "participatory action research"[Title/Abstract] OR "participatory research"[Title/Abstract] OR "mixed-method*"[Title/Abstract] OR "mixed method*"[Title/Abstract] OR interview*[Title/Abstract] OR observation[Title/Abstract] OR "field note*"[Title/Abstract] OR fieldwork[Title/Abstract] OR "field-work"[Title/Abstract] OR "field work"[Title/Abstract] OR "qualitative survey"[Title/Abstract] OR "qualitative questionnaire"[Title/Abstract] OR "focus group*"[Title/Abstract] OR "diaries"[Title/Abstract] OR "journal entr*"[Title/Abstract] OR "visual data"[Title/Abstract] OR "open-ended"[Title/Abstract] OR "open ended"[Title/Abstract] OR "thematic analysis"[Title/Abstract] OR "framework method"[Title/Abstract] OR "framework analysis "[Title/Abstract] OR "conceptual analysis"[Title/Abstract] OR "descriptive analysis"[Title/Abstract] OR theme*[Title/Abstract] OR categor*[Title/Abstract])

**EMBASE**

'breast cancer'/exp OR (breast:ab,ti AND (cancer:ab,ti OR tumor:ab,ti OR tumour:ab,ti OR carcinoma:ab,ti OR neoplasm:ab,ti))

AND

financ*:ti OR money:ti OR econom*:ti OR 'financial distress'/exp

AND

('phenomenology'/exp/mj OR 'ethnographic research'/exp/mj OR 'behavioral observation'/exp/mj OR empirical research'/exp/mj OR 'interview'/exp/mj OR 'grounded theory'/exp/mj OR 'qualitative research'/exp/mj OR qualitat*:ab,ti OR 'qualitative research':ab,ti OR 'qualitative stud*':ab,ti OR 'qualitative design':ab,ti OR 'grounded theory':ab,ti OR narrat*:ab,ti OR ethno*:ab,ti OR phenomenol*:ab,ti OR 'action research':ab,ti OR 'action-research':ab,ti OR 'participatory action-research':ab,ti OR 'participatory action research':ab,ti OR 'participatory research':ab,ti OR 'mixed-method*':ab,ti OR 'mixed method*':ab,ti OR interview*:ab,ti OR observation:ab,ti OR 'field note*':ab,ti OR fieldwork:ab,ti OR 'field-work':ab,ti OR 'field work':ab,ti OR 'qualitative survey':ab,ti OR 'qualitative questionnaire':ab,ti OR 'focus group*':ab,ti OR 'diaries':ab,ti OR 'journal entr*':ab,ti OR 'visual data':ab,ti OR 'open-ended':ab,ti OR 'open ended':ab,ti OR 'thematic analysis':ab,ti OR 'framework method':ab,ti OR 'framework analysis':ab,ti OR 'conceptual analysis':ab,ti OR 'descriptive analysis':ab,ti OR theme*:ab,ti OR categor*:ab,ti)

**CINAHL**

(MH "Financial Stress") OR TI ( financ* OR money OR econom* )

AND
(MH "Breast Neoplasms") OR TI ( (breast AND (cancer OR tumor OR tumour OR carcinoma OR neoplasm) ) OR AB ( (breast AND (cancer OR tumor OR tumour OR carcinoma OR neoplasm) ) OR AB ( (breast AND (cancer OR tumor OR tumour OR carcinoma OR neoplasm) ) OR AB ( (breast AND (cancer OR tumor OR tumour OR carcinoma OR neoplasm) )

AND
(MH "Qualitative Studies") OR (MH "Empirical Research") OR (MH "Focus Groups") OR (MH "Phenomenological Research") OR (MH "Ethnographic Research") OR (MH "Grounded Theory") OR (MH "Interviews") OR TI ( qualitat* OR "qualitative research" OR "qualitative stud*" OR "qualitative design" OR "grounded theory" OR narrat* OR ethno* OR phenomenol* OR "action research" OR "action-research" OR "participatory action-research" OR "participatory action research" OR "participatory research" OR "mixed-method*" OR "mixed method*" OR interview* OR observation OR "field note*" OR fieldwork OR "field-work" OR "field work" OR "qualitative survey" OR "qualitative questionnaire" OR "focus group*" OR "diaries" OR "journal entr*" OR "visual data" OR "open-ended" OR "open ended" OR "thematic analysis" OR "framework method" OR "framework analysis " OR "conceptual analysis" OR "descriptive analysis" OR theme* OR categor* ) OR AB ( qualitat* OR "qualitative research" OR "qualitative stud*" OR "qualitative design" OR "grounded theory" OR narrat* OR ethno* OR phenomenol* OR "action research" OR "action-research" OR "participatory action-research" OR "participatory action research" OR "participatory research" OR "mixed-method*" OR "mixed method*" OR interview* OR observation OR "field note*" OR fieldwork OR "field-work" OR "field work" OR "qualitative survey" OR "qualitative questionnaire" OR "focus group*" OR "diaries" OR "journal entr*" OR "visual data" OR "open-ended" OR "open ended" OR "thematic analysis" OR "framework method" OR "framework analysis " OR "conceptual analysis" OR "descriptive analysis" OR theme* OR categor* )

**PSYCINFO**

(financ* or money or econom*).ot.

AND

(exp Breast Neoplasms/ OR (breast and (cancer or tumor or tumour or carcinoma or neoplasm)).mp.)

AND

(qualitat* or "qualitative research" or "qualitative stud*" or "qualitative design" or "grounded theory" or narrat* or ethno* or phenomenol* or "action research" or "action-research" or "participatory action-research" or "participatory action research" or "participatory research" or "mixed-method*" or "mixed method*" or interview* or observation or "field note*" or fieldwork or "field-work" or "field work" or "qualitative survey" or "qualitative questionnaire" or "focus group*" or "diaries" or "journal entr*" or "visual data" or "open-ended" or "open ended" or "thematic analysis" or "framework method" or "framework analysis " or "conceptual analysis" or "descriptive analysis" or theme* or categor*).ab,ot.

**SCOPUS**

( TITLE-ABS-KEY ( qualitat* OR "qualitative research" OR "qualitative stud*" OR "qualitative design" OR "grounded theory" OR narrat* OR ethno* OR phenomenol* OR "action research" OR "action-research" OR "participatory action-research" OR "participatory action research" OR "participatory research" OR "mixed-method*" OR "mixed method*" OR interview* OR observation OR "field note*" OR fieldwork OR "field-work" OR "field work" OR "qualitative survey" OR "qualitative questionnaire" OR "focus group*" OR "diaries" OR "journal entr*" OR "visual data" OR "open-ended" OR "open ended" OR "thematic analysis" OR "framework method" OR "framework analysis " OR "conceptual analysis" OR "descriptive analysis" OR theme* OR categor* ) AND TITLE ( financ* OR money OR econom* ) AND TITLE-ABS-KEY ( breast AND ( cancer OR tumor OR tumour OR carcinoma OR neoplasm ) ) )

**WEB OF SCIENCE**

financ* OR money OR econom* (Title) and breast AND ( cancer OR tumor OR tumour OR carcinoma OR neoplasm) (All Fields) and qualitat* OR "qualitative research" OR "qualitative stud*" OR "qualitative design" OR "grounded theory" OR narrat* OR ethno* OR phenomenol* OR "action research" OR "action-research" OR "participatory action-research" OR "participatory action research" OR "participatory research" OR "mixed-method*" OR "mixed method*" OR interview* OR observation OR "field note*" OR fieldwork OR "field-work" OR "field work" OR "qualitative survey" OR "qualitative questionnaire" OR "focus group*" OR "diaries" OR "journal entr*" OR "visual data" OR "open-ended" OR "open ended" OR "thematic analysis" OR "framework method" OR "framework analysis " OR "conceptual analysis" OR "descriptive analysis" OR theme* OR categor* (All Fields)

**Appendix C.** Reports not included

**Identification of studies via databases and registers – Excluded (n= 12)**

**Reports not retrieved (n= 1)**

1. Zhirong, J., Xiang, L., Min, Q., Lin, L. (2024) A qualitative study of self-advocacy experience in patients with breast cancer. *Journal of Nurses Training*, 39(4),441-445.

**Non-qualitative (n= 2)**

1. Gutnik, L., Fish, L.J., Gallagher, J., Greenup, R. (2022) Cost communication in breast cancer surgery. *Annals of Surgical Oncology*, 29(2), S409.
2. Pitr, K. (2021) Economic and Social Aspects of Secondary Lymphedema following Treatment of Breast Cancer. *Clinical Social Work and Health Intervention*, 12(2), 35-38.

**Non-financial toxicity (n= 3)**

1. Denyse T., Martin K.J., Pageot Y.K., Denise DeLuz K., Kim J.H.J., Owoyemi P., Stanton A.L. (2022) Project SOAR: The strong black woman schema in the breast cancer context. Cancer Research, 82(4).
2. Chand, R., Selvamani B., Balukrishna, Prasad J. (2014) Econommic evaluation and assessment of early toxicity of hypofractioned radiotherapy compared to standard fractionation in breast cancer. Journal of Cancer Research and Therapeutics, 10(S31), S1.
3. Ammar-Shehada, W., Bracke, P., & Ceuterick, M. (2024). Experiences of social burden amongst survivors of breast cancer in Gaza: A qualitative study. European journal of oncology nursing : the official journal of European Oncology Nursing Society, 73, 102716. https://doi.org/10.1016/j.ejon.2024.102716

**Conference proceedings (n= 7)**

1. Jones, S., Yi, J., Henrikson, N., Panattoni, L., Shankaran, V. (2023) “We shouldn’t have to worry”: financial hardship after breast cancer diagnosis. *Psycho-oncology*, 32(87), S1.
2. Mokokwe L.T., Obasohan M., Ralefala T., Barg F., Mosepele M., Gross R., Martei Y.M. (2022) Patient Socioeconomic and Cultural Factors Associated With Fidelity to Guideline-Concordant Breast Cancer Therapy Delivery in Botswana. JCO Global Oncology, 8(9), S1.
3. Chen, L., Lu, Q. (2021) Financial and work challenges chinese American breast cancer survivors face: a qualitative study. Ann Behav Med, 55(1), S1-S618.
4. Vigilance J., Harewood H., Greaves N. (2018) A qualitative exploration of the experiences and perceptions of women living with a diagnosis of breast cancer in Barbados: financial loss. JCO Global Oncology, 4(33s), S1.
5. Ng, S. et al. 428P Financial toxicity and unmet supportive care needs in UK breast cancer care: CASCARA study analysis through structural vulnerability and family systems theory. ESMO Open, Volume 10, 105000
6. Keogh, R. et al. 247P Financial and time burden associated with living with a metastatic breast cancer (MBC) diagnosis in Ireland: An all-Ireland patient-led survey (CTRIAL-IE 23-05). ESMO Open, Volume 9, 103268
7. Kong Y.C., Wong L.P., Bhoo-Pathy N.T., Taib N.A., Yusof M.M., Aziz A.F., et al. (2019) Financial needs of households affected by breast cancer in a middle-income setting. Supportive Care in Cancer, 27(1), S213-S214.

**Identification of studies via citation searching – Excluded (n= 11)**

**Reports not retrieved (n= 3)**

1. G. Yingxue, L. Huini, J. Xin, et al., “Financial Toxicity Experiencesin Breast Cancer Patients: A Longitudinal Qualitative Study,” Psycho-logical Monthly 18, no. 10 (2023): 13–16.
2. R. Saranya, V. Nallepalli, S. R, et al., “European Journal of Molec-ular & Clinical Medicine Lived Experience of Rural Indian Women WithLow Socioeconomic Backgrounds Who Have Undergone Mastectomiesfor,” Breast Cancer 7, no. 8 (2023): 4761–4772.
3. J. Y. Ruan, C. Liu, Y. Kuang, et al., “Coping Strategies for FinancialToxicity in Young Female Breast Cancer Survivors : A QualitativeStudy,” Mil Nurs 39, no. 8 (2022): 29–33.

**Non-qualitative (n= 1)**

K. Parsekar, S. Howard Wilsher, A. Sweeting, A. Patel, and R.Fordham, “Societal Costs of Chemotherapy in the UK: An Incidence‐Based Cost‐Of‐Illness Model for Early Breast Cancer,” BMJ Open 11,no. 1 (2021): e039412, https://doi.org/10.1136/bmjopen‐2020‐039412.

**Non-financial toxicity (n= 4)**

1. Wang, L., Geng, X., Ji, L., Lu, G., & Lu, Q. (2020). Treatment decision-making, family influences, and cultural influences of Chinese breast cancer survivors: a qualitative study using an expressive writing method. *Supportive care in cancer : official journal of the Multinational Association of Supportive Care in Cancer*, *28*(7), 3259–3266.
2. Rocque, G. B., Rasool, A., Williams, B. R., Wallace, A. S., Niranjan, S. J., Halilova, K. I., Turkman, Y. E., Ingram, S. A., Williams, C. P., Forero-Torres, A., Smith, T., Bhatia, S., & Knight, S. J. (2019). What Is Important When Making Treatment Decisions in Metastatic Breast Cancer? A Qualitative Analysis of Decision-Making in Patients and Oncologists. *The oncologist*, *24*(10), 1313–1321.
3. White-Means, S., Dapremont, J., Davis, B. D., & Thompson, T. (2020). Who Can Help Us on This Journey? African American Woman with Breast Cancer: Living in a City with Extreme Health Disparities. *International journal of environmental research and public health*, *17*(4), 1126.
4. Pisu, M., Martin, M. Y., Shewchuk, R., & Meneses, K. (2014). Dealing with the financial burden of cancer: perspectives of older breast cancer survivors. *Supportive care in cancer : official journal of the Multinational Association of Supportive Care in Cancer*, *22*(11), 3045–3052.

**Mixed populations (n= 3)**

1. C. J. Enzler, S. Torres, J. Jabson, A. Ahlum Hanson, and D. J.Bowen, “Comparing Provider and Patient Views of Issues for Low‐Resourced Breast Cancer Patients,” Psycho‐Oncology 28, no. 5 (2019):1018–1024, https://doi.org/10.1002/pon.5035.
2. P. R. Sherwood, H. S. Donovan, M. Rosenzweig, R. Hamilton, andC. M. Bender, “A House of Cards: The Impact of Treatment Costs onWomen With Breast and Ovarian Cancer,” Cancer Nursing 31, no. 6(2008): 470–477, https://doi.org/10.1097/01.ncc.0000339255.75947.a8.
3. K. Kayser, L. Smith, A. Washington, L. M. Harris, and B. Head,“Living With the Financial Consequences of Cancer: A Life CoursePerspective,” Journal of Psychosocial Oncology 39, no. 1 (2021): 17–34,https://doi.org/10.1080/07347332.2020.1814933.

**Appendix D.** CASP checklist Quality assessment

|  | *1. Was there a clear statement of the aims of the research?* | *2. Is a qualitative methodology appropriate?* | *3. Was the research design appropriate to address the aims of the research?* | *4. Are the study’s theoretical underpinnings clear, consistent and conceptually coherent?* | *5. Was the recruitment strategy appropriate to the aims of the research?* | *6. Was the data collected in a way that addressed the research issue?* | *7. Has the relationship between researcher and participants been adequately considered?* | *8. Have ethical issues been taken into consideration?* | *9. Was the data analysis sufficiently rigorous?* | *10. Is there a clear statement of findings?* | *11. How valuable is the research?* |
| --- | --- | --- | --- | --- | --- | --- | --- | --- | --- | --- | --- |
| **STUDIES** |  |  |  |  |  |  |  |  |  |  |  |
| Ko (2025) | yes | yes | yes | no | yes | yes | yes | yes | yes | yes | yes |
| Marshall (2025) | yes | yes | yes | no | No | can’t tell | no | yes | no | yes | yes |
| Do (2024) | yes | yes | yes | yes | yes | yes | no | yes | yes | yes | yes |
| Jones (2024) | yes | yes | yes | no | no | yes | no | no | yes | yes | yes |
| Khajoei (2024) | yes | yes | yes | no | yes | yes | can't tell | yes | yes | yes | yes |
| Patra (2024) | yes | yes | yes | no | can't tell | can't tell | No | no | can't tell | yes | can't tell |
| Ruan (2024) | yes | yes | yes | yes | yes | yes | can't tell | yes | yes | yes | yes |
| Waters (2024) | yes | yes | yes | no | yes | yes | can't tell | no | can't tell | yes | can't tell |
| Khazi (2023) | yes | yes | yes | no | can't tell | yes | no | yes | can't tell | yes | no |
| Kolawole (2023) | yes | yes | yes | no | yes | yes | yes | yes | no | yes | can't tell |
| Lee (2023) | yes | yes | yes | no | yes | yes | no | can't tell | yes | yes | yes |
| Neilson (2023) | yes | yes | yes | no | yes | yes | yes | no | can't tell | yes | yes |
| Walton (2023) | yes | yes | yes | no | yes | yes | no | yes | no | can't tell | can't tell |
| Aitken (2022) | yes | yes | yes | no | yes | can't tell | can't tell | yes | can't tell | yes | yes |
| Marshall (2022) | yes | yes | yes | no | yes | yes | yes | can't tell | yes | yes | can't tell |
| Prabandari (2022) | yes | yes | yes | no | yes | yes | yes | yes | no | yes | yes |
| Agha (2021) | yes | yes | yes | no | can't tell | can't tell | yes | can't tell | no | yes | yes |
| Gharzai (2021) | yes | yes | yes | no | yes | yes | can't tell | can't tell | can't tell | yes | yes |
| Lewis (2021) | yes | yes | yes | yes | yes | yes | yes | yes | can't tell | yes | yes |
| Oshima (2021) | yes | can't tell | no | no | can't tell | yes | can't tell | yes | yes | yes | yes |
| Chebli (2020) | yes | can't tell | yes | no | yes | yes | no | can't tell | yes | yes | yes |
| Iddrisu (2020) | yes | yes | yes | no | yes | yes | no | no | no | yes | can’t tell |
| Kong (2020) | yes | yes | yes | no | can't tell | can't tell | no | yes | can't tell | yes | yes |
| Dean (2019) Cancer | yes | yes | yes | no | yes | can't tell | can't tell | yes | no | yes | yes |
| Dean (2019) SCC | yes | yes | yes | no | yes | can't tell | no | can't tell | can't tell | yes | yes |
| Japhet (2019) | yes | yes | yes | yes | yes | yes | no | yes | can't tell | yes | yes |
| Nolan (2019) | yes | yes | yes | yes | yes | yes | no | can't tell | no | yes | yes |
| Pisu (2019) | yes | yes | yes | no | yes | yes | yes | yes | no | yes | yes |
| McEwan (2014) | yes | yes | yes | yes | yes | yes | can't tell | yes | yes | yes | yes |
| Klimmek (2010) | yes | yes | yes | no | yes | can't tell | can't tell | yes | yes | yes | can't tell |
| Darby (2009) | yes | yes | yes | no | can't tell | can't tell | can't tell | can't tell | yes | yes | yes |
| Lauzier (2005) | yes | yes | yes | no | yes | yes | can’t tell | yes | yes | yes | yes |
