# Supplementary material for: “You're really walking along the razor's edge”: A meta-synthesis on the existential cost of breast cancer related to financial toxicity
Source: Breast. 2026 Jan 20;86:104705. doi: 10.1016/j.breast.2026.104705 (PMC12860959; doi:10.1016/j.breast.2026.104705)
Supplement: Multimedia component 2 [file mmc2.docx]

**Title**

*“You’re really walking along the razor’s edge”*: A meta-synthesis on the existential cost of breast cancer related to financial toxicity

| **Author (year)** | **Authors' interpretation (second order constructs)** | **Participants' voices (first order constructs)** | **Interpretation** | **Labels** |
| --- | --- | --- | --- | --- |
| **Ko (2025)** | **Employment disruption** Most participants described employment disruption, including quitting jobs, changing from full-time to part-time employment, or being on disability post-BC diagnosis, which contributed to income loss. Patients employed in physically demanding jobs (e.g., farm workers) had challenges performing work tasks, imposing a greater risk of losing employment. Working in a strenuous environment with high temperatures and physically taxing labor was identified as posing considerable risks to employees’ physical and mental well-being and potentially leading to employment disruptions. | *I used to work 40 hours from the beginning of the grape harvest in April until October or part of November… I haven’t worked for two years now…. with my hand that is swollen. There were times when it hurt a lot… (Patient, 56 YO [years old]).* | Due to health problems (swollen limb) I was unable to work for two years. | Difficulties in returning to work due to side effects |
|  |  |  |  |  |
|  |  | *I had my savings from the work I had all these years. I lost my job and my savings that I had. My savings are all gone because of my cancer diagnosis. (Patient, 50 YO)* | I lost my job and had to use all my savings for the diagnosis. | Losing your job |
|  |  |  |  | Depletion of savings, assets, pension funds |
| **Ko (2025)** | Patients’ challenges in maintaining employment arose when their employers made inadequate accommodations for their work schedule to meet their health care needs. Some patients experienced resistance to work-related accommodations from their employers. | *I actually had two jobs lined up, but they (employers) ended up telling me that they couldn’t proceed. I had to do a 90-day probation, and my surgery was around the corner, and they couldn’t accommodate it….You know, they [employers] don’t understand the sickness we’re going through, and we’re trying to make money to support our family (Patient, 36 YO).* | I have not received any accommodations from my employer to continue the job I was on probation in. | No practical support from employer/colleagues |
| **Ko (2025)** | **Medical-related and non-medical related financial costs A. Medical related costs** Patients experienced high out-of-pocket costs, copayments, and deductibles. One patient reported her accumulated unpaid co-pay bills due to multiple tests and treatments: | *Well, it is precisely all the expenses resulting from being sick and having to go out for check-ups, sometimes two or three times a week…. That money, I could save or invest in something else…. I have a bill for $2500, which was not covered by the health insurance. (Patient, 50 YO).* | I have had recurring medical expenses that were not covered by insurance | Insurance: limited health coverage |
| **Ko (2025)** | Co-payments varied by the type of insurance and treatment. For those who had limited health insurance coverage such as corporation-supported health insurance, their burden for out-of-pocket costs was greater. | *Patients also incurred indirect medical costs, such as paying out-of-pocket for special creams, equipment, and clothing. A patient stated, “With radiation, I had to buy some lotions, and Medi-Cal [state insurance] did not cover. It [Lotion] is to help with the peeling and stuff, but it was a little bit pricey (Patient, 55 YO).* | I had to pay medical expenses (post-radiotherapy lotions) that were not covered by public health insurance. | Insurance: limited health coverage |
| **Ko (2025)** | **B. Non-medical related costs** Cancer-related non-medical related expenses (e.g., transportation, childcare, utilities) contributed to the patient’s financial hardship. Concerns about gasoline and transportation expenses were frequently cited by patients and family members, especially for those traveling from rural areas. “I was filling up my car. When gas prices are sky-high, it is up to $120 or $130 a week to go to all my appointments… (Patient, 50 YO).” For patients with limited support, financial strain can be even more difficult. Those who required support with household responsibilities or childcare due to their limited mobility, engaging the services of someone to help them resulted in supplementary expenses. | *If you are by yourself, you have to have someone or hopefully a family member to come in. However, if not, you have to pay someone to actually come in and help you with the household chores and just certain daily things (Patient, 50 YO).* | I didn't have anyone to help me and I had to pay someone to help me with the housework. | Incurring non-medical expenses |
|  |  |  | I had to pay non-medical expenses (cost of transportation) | Incur non-medical expenses (transportation) during treatment |
| **Ko (2025)** | **Struggles to meet basic needs** Patients experiencing financial hardship frequently encountered challenges in fulfilling their fundamental necessities, including adequate food, appropriate clothing, and stable housing. Food insecurity emerged frequently, and patients and families employed various coping strategies to make ends meet. Some had to cut expenses on buying groceries, prioritizing only essential items to buy. “Now we have to do it [grocery shopping] every two weeks because we try to manage our food better” (Daughter, 27 YO). Others also had to give up certain high-cost food items such as meat, “I said we would have to make white rice with beans and forget about meat, chicken, and eggs. What a horror!” (Patient, 58 YO). |  |  |  |
| **Ko (2025)** | After depleting their savings, some patients applied and received formal support, such as social security disability benefits, but it wasn’t enough to cover expenses for their necessities. How to manage the deficit in income remained a concern for the future. A patient shared: | *You are paying rent and having expenses on necessities, then [the savings] start to deplete… Then they give me the disability [benefit], approximately $30 to $40, $32 for a day. The amount of disability [benefit] is not the same as [the amount] of salary. It was [lower] almost by half.…What am I going to do if the money is not going to be enough?” (Patient, 65 YO).* | The income I received from the disability certificate did not match the salary I received from work | Disability benefits were not enough to cover expenses |
| **Ko (2025)** |  | *Sometimes, we didn’t even have enough [money] to buy tortillas or anything. I had a very good neighbor who saw we had nothing to eat. She was the one who helped us…I could not buy shoes for my son. It was cold then, and I could not buy a sweater for my child. I would sew it if it tore. All of this affected all of us (Patient, 35 YO).* | I didn't have the money to pay for basic necessities | Not having money for essential goods |
| **Ko (2025)** | **Psychological distress** Psychological distress was the most frequently described issue among the participants, particularly by patients and family members. In particular, worries and anxiety about unknown situations and their ability to handle financial challenges were prevalent. “How am I going to pay my bills? How am I going to pay for my car and the gas and, you know… I just knew I have to be strong because I was going to get emotional.” (Patient, 36 YO). The psychological distress of patients had an impact on their families, affecting their emotional and psychological well-being. A family member shared that the patient’s desire to die in her home country caused anxiety for her children, who were concerned about her leaving: |  | I didn't know how I was going to pay my daily expenses and this was a source of stress. | Not having money for essential goods |
| **Ko (2025)** | Both patients and family members often refrained from discussing financial hardship and rather endured their struggles in silence due to concerns regarding its potential impact on psychological distress for both parties. “When I was having trouble with money, I would never let her [patient] know. If I didn’t eat, she wouldn’t know. If I didn’t have gas, she wouldn’t know. I wouldn’t tell her because I didn’t want her to be more stressed. So I would take it upon myself” (Daughter, 23 YO). | *I was very desperate because I knew that we had to make payments and I could no longer help. My husband was the one who had to pay for everything or had to ask my daughters for money to be able to make the payments. It stressed me out a lot, apart from everything that had happened… (Patient, 49 YO)* | My husband and daughter's earnings were needed to pay the expenses | Dependence on family members financially |
|  |  |  |  |  |
|  |  |  | Asking my family for money was a source of stress | Learning to ask for support from others |
| **Ko (2025)** | **Changes in the family’s daily activities and employment** With the patient’s financial challenges in hiring formal caregivers, family members took on different caregiving responsibilities, including babysitting, providing transportation, and offering financial support. However, coordinating their own work schedules and daily activities, such as their children’s school schedules, posed challenges for these family members. A participant who had to rely on her older children to take care of her younger children while attending clinic visits reported how it caused disruptions to the children’s school schedules. | *I had no babysitter and no one to leave them [young children] with. My other children were in school. It was tough…for example, for today, I had to take my other son out of school to stay with my baby. My child tells me that I have so many appointments and so on. Because I have to take them out of school… (Patient, 41 YO).* | I had a hard time managing my young children because I didn't have a babysitter | Unmanageable non-medical expenses |
| **Ko (2025)** | Family members’ involvement in patient care caused them to take time off work, reduce work hours, or quit their jobs, resulting in a loss of income. Patients were concerned about the financial impacts of these work schedule disruptions for their families. “Sometimes, my children had to take a day off to bring me [to the clinic] and back. When you get chemo, you can’t drive, and you need that help… For them, it was one day without pay” (Patient, 65 YO). |  | My children sometimes had to take the day off work (and therefore lose a day's pay) to accompany me to the hospital. | Job changes of family members affecting earnings |
| **Ko (2025)** |  | *My other daughter was a senior at the time, and she was going to care for my grandson while another daughter (grandson’s mom) drove me to the clinic. So, she dropped out of high school to take care of him. That was her sacrifice. She loved school. She’s a smart girl, you know”? (Patient, 55 YO)* | My daughter had to drop out of school to take care of me and other family members | Change of future plans of family members |
| **Ko (2025)** | **Family stress and strain** Patients and families reported changes in family dynamics and relationships while navigating cancer-related financial hardship. Some developed a deeper understanding of each other and became more supportive. “I guess it has gotten us closer and more supportive of one another” (Daughter, 31 YO). However, other patients and families experienced family conflicts due to financial stress, and perceived unequal distribution of responsibilities among family members, all of which led to tension and discord in the family. A family caregiver shared, “I sometimes feel like it is uneven. We are like, “Oh, we’re doing more than you,” or “No, I’m doing more than you because I have to go out and work,” or “No, we’re doing more than you because we’re physically, emotionally and mentally dealing with it…” (Daughter, 25 YO). Participants reported instances of marital tension and discord brought on by financial hardship. Heightened tensions and arguments arose in relation to prioritizing household expenses, such as the payment of bills. Unknown futures and unpredictable situations created a sense of uneasiness and discomfort. | *I would say that it [financial challenges] does add some strain to my marriage because there’s so much out of your control. You can’t predict what you’ll need or what time you’ll need it. So now that we are feeling more of the pinch, you know, it does make it harder to be comfortable, be at peace, be happy…(Patient, 40 YO).* | Financial difficulties have caused pressure in the family and made life less happy and comfortable. | Pressure on partner/family due to financial stress |
| **Ko (2025)** | **Discontinuation or forgoing of cancer treatment** While most patients reported no negative impacts of financial toxicity on their cancer treatment, some patients and HCPs described how financial hardship can cause stoppage/non-compliance to treatment and to routine follow-ups. A patient shared her decision to halt the treatment regimen due to fear of debt and financial burden on her family. | *I told Dr. [redacted] that I had no choice but not to undergo the treatment if I already had the cancer spread… I was worried that all the money would be invested in my health and they [my family] would run out of money. I was very sad for leaving them without money, without food, and without a house because we were going to be left on the street because of my illness (Patient, 50 YO).* | If the cancer had been metastatic I would have refused treatment because I would have had to use all my financial resources to cure myself and my family would have had nothing left. | Decisions on treatment options based on economic resources availability |
| **Ko (2025)** |  | *There was a medication that Medi-Cal (state health insurance) did not cover. It was for my white cell count. I just didn’t have it. I’m like, “Oh, maybe next time”. (Patient, 55 YO)* | Public health insurance did not cover every medication | Insurance: limited health coverage |
| **Marshall (2025)** | **Theme 1: financial toxicities** The majority of study participants reported financial burdens associated with expensive, ongoing cancer treatment, lack of adequate insurance coverage with high out-of-pocket expenses, and difficulty maintaining work schedules and therefore loss of income. |  |  |  |
| **Marshall (2025)** | **Subtheme 1: Costs of cancer treatments and medications** Participants reported being prescribed newer, more expensive, targeted therapies, resulting in financial burden. Such medications were often prescribed when previous treatments became ineffective, with some participants receiving their fourth or fifth line of treatment. Participant 10 described the high cost of a new drug treatment "a new drug they started me on and the cost of it was about $12,000.00 a month. I still have private health insurance as well as being set up on Medicare and so it was being covered and all of a sudden, I guess I'd been on it about eight months, so in december, my insurance wouldn't process it. The medicare portion would be processed but there would be a $ 5,000.00 co-pay." |  | Even though I was 100% covered by insurance, I ended up having to pay part of the expenses. | Insurance: a co-payment was required |
| **Marshall (2025)** | Participants reported having to choose between cancer treatment and other financial priorities. Participant 16 did not qualify for financial assistance because of an annual household income amounting to over $100,000 per year. She still struggled with the financial burden of treatment and could not meet her 10% co-pay on the $28,000 treatments she received every 21 days. She described the need to choose between paying for her treatments and her daughter's college. | *My husband and I are at the point do we pay for our daughter's tuition or do we pay the medical bills. I'm like we pay for her tuition I mean the whole point of me being here is to make sure she has a better life and we'll just pay what we can and stay with [the cancer center] as long as possible"* | I had to decide whether to pay for my own medical care or my daughter's school fees. | Deciding whether to use your own money for treatment |
| **Marshall (2025)** | Some participants had to carry the burden of knowing that their cancer tratment costs were high and would eventually increase. For example participants 17 stated "What I've been able to estimate from all the guessing and estimating is that's going to cost me $10,000 or $12,000 a year". Participant 2 noted "There was a medication that I need that I could not get, it was a shot I needed to get and it ran about $2,200.00, we can't afford that so I didn't get it". Participants were fearful that they may have to go without treatment because of the extensive cost of treatment. Risk of cancer progression as a resul of no treatment was an inconceivable reality. |  | I couldn't take all the therapies (treat myself as I should have) because of the cost | Decisions on treatment options based on economic resources availability |
| **Marshall (2025)** | **Sub-theme 2: limitation of health insurance** Unexpected out of network insurance expenses and uncovered expenses were financially devastating for women in the study, as evidence by the following quote from participant 2, who was mistakenly told by her insurance company that her out of network provider would be considered in network: "So I ended up with a bill for like $ 150,000.00 that my insurance didn't cover, so that was a barriere for a while, and I had to fight for three years and with insurance appeals". MBC patients undergoing treatment must face many physical challenges but trying to navigate insurance claims and/or denials was overwhelming, adding an external layers of stress. |  | My insurance didn't cover the costs and I had to fight for several years | Fighting against insurance companies to obtain one's rights |
|  |  |  | Dealing with compensation claims has been devastating | Insurance: the need to defend oneself against insurance |
| **Marshall (2025)** | Often when drugs were available, they were not covered by insurance. Participant 14 provided an example of this when she stated: "The chemo has not really had the effect that we want it to so we are trying to go outside of the realm of the breast cancer chemos and see if a different chemo would work and you know the insurance companies are really giving us a hard time". |  | Insurance companies are giving me a hard time | Emotional distress related to insurance aspects |
| **Marshall (2025)** | Participant 2 echoed, "The pricing of that you know is always a constant battle between my insurance company and what they are covering and needing prior authorizations and you'are here, you are in pain, and you can't get your medicine because it is going through a prior authorization process so that in itself is just unreal. I can't believ that they put that stress on sick people". |  | Insurance authorization procedures are delaying delivery of medications |  |
|  |  |  | Insurance caused me stress | Emotional distress related to insurance aspects |
| **Marshall (2025)** | The uncertainty of insurance to cover cancer care was considered by some women to be the worst part of treatment. Participant noted that when she lost her regular employment insurance she went through several changes to coverage, which was more costly. She expressed "When I was working, I had insurance which was good and I did not appreciate it until I lost it. the insurance rigamarole has been extremely stressful that's been probably one of the worst things abouth this whole thing is the uncertainty of it. I moved to my work insurance to COBRA which proved to be very expensive and thent when that ran out, I had to go on the marketplace and, holy cow, that was expensive. Then had to change again six months later to another one and then I go on Medicare in June and then we will deal with alla that. It's just like a fight a constant fight to keep my coverage in all of that and yeah, Medicare is going to be another, a whole other thing". |  | I lost my insurance from work, and then I changed several. | Insurance: maintaining cover after leaving work |
|  |  |  | I have experienced the uncertainty of insurance coverage | Uncertainty about insurance coverage |
| **Marshall (2025)** | Filling out forms and providing information to get financial assistance to compensate for inadequate insurance coverage was burdensome. Patients noted apllying for financial assistanza for inadequate insurance coverage was burdensome. Patients noted apllying for financial assistance took time and energy away from them when they were not feeling well from their cancer or cance treatment. Participant 2 described the paperwork burden: "They have all of these gants out there that you can apply for [...] but I would say thsi when people are dealing with cancer.. it can get a little stressful because they want so much information and I get it they want to make sure that its legit and they are not being scammed by people, but it is king of frustrating when you are not feeling well and you have to go here to that oncologist to fill out this and then you have to send this whole packet full of [...] it's just draining." |  | I have spent a lot of time and energy managing the insurance coverage procedures | Complexity of paperwork management of insurance |
| **Marshall (2025)** | **Sub-theme 3: Impact on employment** A diagnosis of cancer and subsequent treatment demands affected work, or plans for work, creating an additiona financial burden. Participants 3 stated: " My grand plan for when my son went to high school was to go back to work, I am not really planning on doing that unless it is some really flexible part time fashion. I just can't make that kind of commitment to someone, a company, or a person. I basically lost my financial freedom. Being able to pay your health insurance is a big part of that too." Participant 5 said: "I had to cut back on work because staying healthy is a full-time job." |  | I had to cut back on work to have time to stay healthy | Reducing work to have more time for health |
|  |  |  | I can only return to work if I am allowed to work part-time | Reducing work hours |
| **Marshall (2025)** | **Theme 2: Supportive care needs** Participants reported reliance on family, friends and at times hired extra help to continue to meet their responsabilities and function in their various roles. At times the women felt their cancer diagnosis isolated that from others a a time when they needed those connections the most. Resources that had been available to them in the pst, and prior to the pandemic, were no longer available. both individual social support and community support were needed. |  |  |  |
| **Marshall (2025)** | **Sub-theme 1: Managing usual responsibilities** Cooking, cleaning, and transportation were activities that participants sought help in managing. Participant 1 shared, "we have people that do the yard here, my husband has people that come in and clean for us. I have my sister who just came down and cooked a ton of meals. I have frineds that will occasionally drop off meals. My mother came for two months although I ended up doing all the cooking and she did the laundry to help out." The same participant described how others help with transportation and childcare when needed. "My older sister dropped my daughter off at school, she kind of took care of my life. I rely on dear friends to pitch in. We have a sitter that's on college that comes on Fridays so I can recover during the weekend so I can be fresh during the week for my daughter when she is in school." |  | Constructs without relevant content to answer research questions | Constructs without relevant content to answer research questions |
| **Marshall (2025)** | Physical limitations prompted participants to seek assistance. For example, Participant 13 stated: "After the brain tumor they wouldn't let me drive for six months. My daughter has been driving me. I know that I'm not like the wayI was before exactly but I still do try to do my laundry, I do go to the grocery store with her once a week. You know at this point I'm not using a walker or a cane but there were times when I have been in the last year." These comments demonstrate the need for additional supportive care and financial resources for managing home transportation needs, especially for women who may not have family nearby to assit. If costs for house chores and maintenance are needed, this adds to the financial burden experienced. |  | I had non-medical expenses for housekeeping and transportation. | non-medical expenses (housekeeping, transportation) |
| **Marshall (2025)** | **Sub-theme 3: community and organizational support** Participant 6 recalled having a nurse navigator available until being diagnosed with metastatic disease, at which time the urse navigator was no longer available. "The nurse navigator was awesome and she even helped me when I was in line to sign in for a PET scan and my insurance has not approved it yet. I called her and she got the ball rolling." |  | I had support from a nurse who helped me start the process to request the PET scan while I was waiting for insurance approval. | Support from healthcare professionals |
| **Do (2024)** | Simultaneously, Hồng’s husband contacted the village head and commune authorities to inquire about applying for official poor household status to receive a monthly cash transfer from the government. However, their request was rejected. As her hometown was recently awarded with the title “New Rural Commune” – awarded by the government to communes in recognition of their success in improving the local infrastructure and standards of living, they instead directed her to apply for the state allowance as a person with a disability. Her disability allowance application was approved and with that Hồng was granted a government-funded insurance with a 100% coverage. Even with the 100% insurance coverage she was entitled to, she was held liable for paying various expenses during each hospital visit: | *Going to hospital is really costly … Every time I need someone to go with me, so the transportation costs alone are 1,000,000 VND (~US $43.86) … With the insurance, they place a cap on hospitalisation of 20,000,000 VND (~US$877.19), which meant that if I am required to stay longer, I must pay out-of-pocket. And for each stay, sometimes the doctors prescribed me some medication that was not available here [at the hospital’s pharmacy], and I had to purchase them from the private pharmacies [out-of-pocket].* | The cost of medical and non-medical expenses is very high and the insurance had a maximum coverage cap | High cost of medical and non-medical expenses |
|  |  |  | I had to pay for some medications out of my own pocket | Incur medical expenses not covered by insurance |
| **Do (2024)** | In every conversation during our ethnography, Hồng frantically spoke about the possibility of opting out of radiotherapy because she could not envisage a viable source of finance for another five-week stay at the hospital for herself and the caregivers. While complete opt-out of biomedical treatment was not a practice reported among the informants, which was largely due to our hospital-based recruitment method, we met many women who had decided to discontinue after undergoing some forms of treatment. This was particularly so when a patient’s treatment regimen involved targeted treatment which came with tremendous cost and low reimbursement. Only few patients enrolled in targeted treatment but even in such cases, many could not complete the recommended regimen. For example, 55-year-old Bˆong from Hue City had requested that her oncologist halve the prescribed dosage from 12 to 6 months, as the whole course of treatment was beyond her ability to pay. In the final appointment with Hồng, she told us because her oncologist recommended that she should have oral hormone therapy for the next five years, she came up with what Dao (2023)describes as ‘a calculative practice’ to navigate regular hospital visits by weighing up different care routes to select the more economical option. Hồng decided to buy her medications (anastrozole) from a local pharmacy at her own expense without visiting the Central Hospital to get the insured dose, “because the train tickets cost me more.” She planned to go back for her quarterly follow-up appointments only if she could borrow the money from her relatives. These coping measures were similarly adopted by other women interviewed in our study who were diagnosed with positive hormone receptor status – which is found in approximately 80% of breast cancer patients (Giaquinto et al., 2022). Due to existing drug dispensing policies, insured patients like Hồng were only entitled to receive a thirty-day dose of their medications in each hospital visit. Some medications for advanced-stage breast cancer, such as Aromasin (chemical name: exemestane), and Afinitor (chemical name: everolimus)1for postmenopausal women could only be dispensed on a ten-day dose, and hence patients needed three visits to the hospital every month to fully adhere to their prescription. Considering the substantial costs, when patients could not travel to obtain them from the hospital, in most cases, they would miss a recommended dose completely. In Hồng’s case, she adopted individualised responsibility to maintain her treatment compliance rather than depend on the health system to provide the insured medications, and simultaneously controlled the financial risks associated with a long trip to the hospital. Her story exemplifies how fate is turned into problem of the self-management of calculable risks | *Hồng confided in us that to pay for medical and non-medical expenses such as transportation and meals she had to seek loans from “any possible sources,” as her family’s savings had depleted. These included zero-interest loans from her siblings and relatives and a credit from a local post office at an interest rate of 15% per annum. Simultaneously, Hồng took another loan worth 50,000,000 VND (~US$2193) from a commercial bank using her family’s land use right certificate as collateral. This, however, meant that her family would not be able to borrow more money from any formal financial institutions if they wanted to expand their farming activities or in case of other shocks. Borrowing money as a coping measure was especially common among patients from limited-income households who had already spent a significant proportion of their resources on financing burdensome treatment costs. While participants mentioned relying on their social network for help with managing medical and day-to-day expenses during treatment, it usually did not suffice given that those people were likely to have similar levels of wealth to that of the patients. Therefore, cancer patients’ families resorted to loans from credit institutions or the underground market. For instance, 46-year-old Xuˆan, a street vendor and single mother from Khanh Hoa, sought an underground loan which charged her a usurious interest rate of 10%/month to pay for diagnostic tests, surgical costs, and inpatient stays because she had no insurance by the time of her diagnosis. Xuˆan however could not afford chemotherapy and radiotherapy and left after the mastectomy was finished.* | I decided not to do the therapy or to undergo it discontinuously because of its cost. | Decisions on treatment options based on economic resources availability |
|  |  |  | I paid for the anastrazole out of my own pocket because the train ticket to the central hospital would have cost me more. | Decisions on treatment options based on economic resources availability |
|  |  |  | Family savings have been depleted to pay non-medical costs | Depletion of savings, assets, pension funds |
|  |  |  | I asked for a medical loan even though it had an interest rate to repay | Applying for loans |
| **Do (2024)** | **Seeking formal support** **Cancer disclosure as a strategy** For instance, 56-year-old Tr`a, a farmer from Quang Tri, mentioned about the support, including in-kind (such as food and clothes) and cash from people in her village: “A neighbour in my village, he lent me that money. Many others living nearby heard my story, and they came to gift me 50,000 VND (~US$2.20) or 100,000 VND (~US$4.39).” Tr`a described the tactic of making her cancer status known to people in the community, similarly to other women we met during our fieldwork. Having no insurance by the time of receiving a breast cancer diagnosis in 2012 because she could not afford to enrol her family in the voluntary insurance scheme, her family took out a personal loan using their house as collateral to finance her treatment. She explained how the annual interest rate was partially repaid: "*I did not try to hide my illness from others in the neighbourhood, because when I told them I had cancer, they came to visit me. They also helped give me some money. I could use the money to pay back the interest rate."* |  | The neighbors in my village gave me some money | Economic support from the community |
|  |  |  | My family took out a loan by mortgaging the house | Family members applyed for loans |
|  |  |  | I shared my diagnosis with the community and received emotional and financial support | Economic and emotional support from the community |
| **Do (2024)** | More importantly, disclosure carried further benefits for patients because it could influence the process of applying for state welfare for a poor household or disability support. This official recognition may provide them with longer-term entitlements, most notably, a concessional insurance card and monthly cash transfer. According to existing regulations on poverty administration, a household might apply for social welfare themselves by submitting an application form to the local authorities. This process will be accelerated when a household is nominated by other villagers, especially the village head who has a determining role in identifying potential beneficiaries and assists with the application process (Groce et al., 2017). As Tr`a explained: “Other villagers also cared about me because in my family, the mom was sick, the son was also sick. They nominated me to get the poor household status.” |  | Communicating the diagnosis influenced the process for requesting formal recognition of disability or poverty status | Disclosure affects the request of forma lsupport |
|  |  |  | The welfare application was speeded up because my family had been nominated by other members of the village who also helped me with the application process | Practical support from the community |
| **Do (2024)** | **Navigating the politics of social welfare** To mitigate the impacts of inadequate insurance coverage and manage the tremendous expenditure demanded in cancer treatment, seeking a source of longer-term support from the government was a kind of ‘work’ that patients and families actively engaged in. The amount of work to make oneself eligible for some form of allowance from the government was often intensive and strenuous. Our interviews revealed that women carefully researched social security schemes applicable to breast cancer patients. For example, 36-year-old Hˆom, an active member of the peer network, proudly considered herself as a key informant about social protection regulations and entitlements. She often shared her application experience with fellow patients by writing Facebook posts. Following her diagnosis of stage IIB breast cancer and treatment of a metastasis, Hˆom spent months seeking information about the social benefits for people with a disability from her village leader, the local authorities, her peers, and the Internet. She vividly recollected the process which had happened more than three years prior to our interview: | *I brought my medical record and identification card to the social policy department. There was a young female officer there who reviewed my profile. She then asked me to fill in a form and to write a declaration letter. I then needed to get the village head and the commune secretary of the Communist Party to sign on that letter* | I have tried to ask for formal and long-term financial support from the government | Formal social support |
| **Do (2024)** | In contrast with Hˆom’s successful application, our interviewees illustrated the uncertainty inherent to the process of certifying social protection beneficiaries among patients residing in other jurisdictions despite their prolonged, laborious efforts |  | I was uncertain whether my benefit application would be successful | Uncertainty about formal support |
| **Do (2024)** | However, her application was only approved with a health insurance card for a near-poor household with 95% coverage without any cash transfer: “My family was already very poor. But later they told me I was not qualified because | *I could still walk on my own and they thought I was able to continue earning money.”* | I didn't qualify for support because formal systems thought I was still able to earn | Lack of support from formal support systems |
| **Do (2024)** | To mitigate the uncertainty associated with social welfare verification, some patients resorted to paying a bribe to a government official in the hope that it could accelerate the approval process and increase their chance of success. For instance, a former childcare educator Hay from Quang Tri province had already enrolled in the compulsory insurance scheme by the time of her primary diagnosis. With this, she was covered for 80% of her treatment costs. However, when she met fellow patients at the hospital, she was told that “paying only 20% of the remaining costs was already a burden.” Hay attempted to seek a “poor household” certificate by paying a bribe to a commune officer and village head. When it was successful, Hay was granted a concessional insurance card with 100% coverage with a monthly cash transfer. |  | I paid a bribe to a city official to receive 100% insurance coverage | non-legal methods to speed up procedures |
| **Do (2024)** | Paying a bribe was a common practice when patients needed to obtain the approval letter to register their insurance at a national-tier hospital with more advanced oncology services. Like Hay, Minh had already had an insurance card as she was working as a civil servant by the time of her diagnosis. However, she still had to pay for roughly 70% of the surgical costs and all other hospitalisation expenses as a bypassing patient because the insurance transfer had not yet been cleared at the time of her mastectomy. Just before chemotherapy, she paid a bribe to a contact at the province’s office dealing with health insurance in order to facilitate the procedure of having the transfer approval which allowed her to receive full insurance entitlements at the hospital of her choice. The cases of Minh and Hay exemplify how patients have become very “active” navigating state bureaucracies around health insurance and poverty administration throughout the process of seeking cancer treatment. |  | The insurance transfer did not happen in time so I had out of pocket medical expenses | Financial support not timely |
|  |  |  | I paid a bribe to get the insurance rights I was entitled to | non-legal methods to speed up procedures |
| **Do (2024)** | Beyond survivorship: altering life trajectories Due to its chronicity and time commitment, pursuing breast cancer treatment causes remarkable disruptions to patients’ employment and earning capacity, which appeared more pronounced for older women. Because of their age and the acuteness of their health condition that left them with limited ability to seek new opportunities, the financial consequences on those women were often more difficult to manage. 47- year-old Hay, for example, a former childcare educator whose husband suddenly passed away due to a stroke at the time of her mastectomy, had to shift to a factory job: | *My working time [at the childcare] was not flexible. Now that I still have follow-up care, for some appointments I must take leave for 2 or 3 days. I decided then that I would quit because I did not want to affect my school … But because I retired earlier, my pension was reduced a lot … I’ve been working in a new job in a factory, assembling bottles and cleaning … With that I have more money to raise my younger son who is now at high school.* | I retired early but since my income was too low I started working again to earn more money which I used to pay for my son's education | Inability to interrupt work |
| **Do (2024)** | Meanwhile, for other patients who were self-employed in the agricultural sector (like the case of Hồng) or working under a casual contract with little or no income protection, pursuing time-extensive treatment equated to a complete loss of income. These impacts continued beyond the completion of hospital-based treatment. As previous accounts illustrate, many patients were left with little choice but to become indebted while they themselves had lost the capacity to resume the same level of income they had earned in the pre-cancer period. These compounding burdens forced them into precarity, including, for instance, having their sources of income. This strategy was particularly a solution among families residing in rural regions. In Xuˆan’s case, for instance, a single mother to three children aged under 20, her eldest son Bảo permanently migrated to Ho Chi Minh City, 500 km from their hometown, after her cancer diagnosis. The income from her street food stall and her son’s then casual job could not be sustained after she underwent a mastectomy and a lengthy inpatient stay for which she was not insured. Bảo relocated to obtain work for a paint-manufacturing business in Ho Chi Minh City and sent home a monthly remittance to help Xuˆan pay for her debt and support his younger sibling’s education. He also obtained a two-year loan from his employer to cover their debt and pay for expenses related to Xuˆan’s follow-up care. As affected families made hard calculations as to whose needs should be prioritised and whose would be forfeited, in many circumstances they had to choose between the pressing health need of a member diagnosed with cancer and the educational opportunities of their young children. For example, for Ninh, a 51-year-old woman from Quang Binh province, the diagnosis of breast cancer shattered her son’s educational prospects, as he faced the financial pressure from his mother’s expensive treatment: | *My son only finished high school. At that time I fell sick [with breast cancer], so he did not go to university. He just went for a shorter vocational training so that he could go on to earn money soon … My life has already been tough. If I had had more money back then, I would have sent him to college. But I did not. I feel so much pity for him.* | I couldn't pay my son's college fees because I had just received the diagnosis | Change of future plans of family members |
|  |  |  | I completely lost my income as I am a farm worker and could not work | Perceived reduction in earnings due to absence from work |
|  |  |  | I had to go into debt | Indebtedness |
|  |  |  | I moved to change jobs so I could send money home to support my youngest son | Work changes based on earnings |
|  |  |  | I asked my employer for a loan | Applying for loans to employer |
| **Do (2024)** | For affected young women, dropping out of school is likely to lead them to marriage at a young age given the job scarcity in rural areas and their limited capacity to migrate to urban cities in search of employment. For instance, for a year Khanh had to be accompanied by her then 17-year-old daughter to radiotherapy treatment in Danang City, a 3-h journey by bus from her hometown in Thua Thien Hue. This was during the period when her daughter was preparing for university entrance exams. Khanh’s advanced cancer diagnosis prompted her daughter to abandon her university dream to remove the burden of paying for university tuition fees and instead took on a casual job. Not long after that, the daughter got married: | *When she was working at the clothes store, she met a man 5 years her senior who was a timber trader … Then she came back home one day and told me she wanted to marry him. She was only 19 years old then … So my two younger brothers called for a big family meeting on my behalf to discuss her marriage. In the end everyone agreed that she should [get married] because they were all afraid that I would die soon.* | My daughter gave up her dream of going to university so I wouldn't have to worry about paying for her studies. | Change of future plans of family members |
| **Do (2024)** | The early marriage of Khanh’s daughter was considered by her whole family to be a decision that fulfilled the daughter’s filial duties in light of the parent’s uncertain cancer prognosis. In Vietnam, marriage has value as not only an individual’s achievement, but also their family’s symbolic capital (Nguyen and Hoang, 2019). Such a decision was also a strategy of securing the daughter’s future and easing the financial burden for her family when the breadwinner fell seriously ill. Although the marrying decision may reduce the immediate risks to their life, it foreclosed future opportunities of higher education and job security for Khanh’s daughter. |  | My daughter's early marriage eased the financial burden when I was ill. | Change of future plans of family members |
| **Jones (2024)** | **Causes of financial hardship** To inform model revisions, we examined themes around employment and costs. Costs, unless otherwise pecified, is used as a term inclusive of out-of-pocket care costs, basic needs and other living costs. These factors were key causes of financial hardship in the previous model. |  |  |  |
| **Jones (2024)** | **Employment factors** Having to work versus not needing to work (e.g., being retired) was a cause of financial hardship. While many participants reported supportive work environments, it was clear this was not always the case. Provision of reasonable accommodations (flexible schedule, work from home, someone at work to compensate, flexible work tasks) was key to ensuring participants were able to work and maintain their income. Reasonable accommodations were protective regardless of whether they were provided for the cancer or another condition. This was often dependent on an understanding supervisor and/or coworkers who did not pressure the participants. Employment factors increased the level of financial hardship and financial worry for participants. Sometimes this was due to health insurance being tied to employment. Participants experienced substantial administrative hurdles to access employment-related benefits such as Family and Medical Leave Act (FMLA), short term disability insurance, Americans with Disabilities Act (ADA) accommodations or Consolidated Omnibus Budget Reconciliation Act (COBRA) health insurance. For participants that did access these programs, the stress of the administrative hurdles was high. Some participants choose to forego the benefits simply because of the administrative hurdles or used paid time off (PTO) to avoid the application process and its attending burden and stress. One participant talked about the relief about not having to take on administrative burdens: | *“But you know, I think the- what you call like the red tape. If, you know, I had to do a short-term disability or you know leave of absence I’m sure I could have figured it out. But that’s something I don’t look forward to, even as a manager of people, you know occasionally have to, you know, dig into that and it’s always frustrating so that’s another frustration that I’m glad I avoided.”* | I was stressed because of the administrative procedures to apply for work-related insurance benefits and therefore gave up | Stress caused by health insurance administrative procedures |
|  |  |  | Reasonable accommodations allowed me to work and maintain my salary | Practical support from employer/colleagues |
| **Jones (2024)** | Being self-employed could be precarious due to lack of paid leave, or it could be protective if the participant had employees that continued to run the business. Long-term effects on employment included reduced earnings and delayed retirement. |  | As a freelancer, I did not have paid sick leave | Type of work affects sick pay |
|  |  |  | My employees have kept the company going | Practical support from employer/colleagues |
| **Jones (2024)** | Employment practices primarily center on support for maintaining employment and income during the cancer, such as whether leave is paid or unpaid, availability of reasonable accommodations and the ease with which paid leave and accommodations can be accessed | *“He [boss] was like just go home, you know, just those little things. Like, just go home now, no take the day. You don’t have to take off hours today and if you feel good, you know, just check your email or write up a little summary of something you know and that’ll count and like just those little graces.”* | The people I work with have been supportive | Practical support from employer/colleagues |
|  |  | *“They [workplace] were very supportive, but my health insurance was through my employment, which meant I had to stay working the whole time.”* | I had to continue working because my health insurance was work-related | Continue working to maintain health insurance |
| **Jones (2024)** | **Insurance factors** Insurance coverage was a major driver of financial hardship. Some participants had generous health insurance or multiple health insurance plans and experienced little financial hardship. Others had less generous or more difficult to access health insurance; these participants experienced administrative hurdles to cover care, such as needing to get care recoded by providers or appeal an insurance company decision. Others struggled to balance working with receiving cancer care, particularly with maintaining mployersponsored health insurance. Programs to maintain post-employment employer-sponsored health insurance were cost prohibitive or administratively burdensome. Other unhelpful aspects of health insurance were getting coverage for out of network care, multiple bills for the same episode of care, or providers that did not accept insurance that led to huge bills or inability to access care. Care that was often not covered or insufficiently covered included lymphedema, genetic testing, complementary and integrative medicine and reconstructive surgery. When talking about lymphedema, a common side effect of breast cancer treatment, one participant said: | *“And what I did find out is I tried to research was that if I needed, if I needed significant lymphatic massage, say through a massage therapist who’s able to do that, nobody takes insurance for it. Nobody.”* | I experienced financial difficulties because of my health insurance | Insurance: limited health coverage |
|  |  |  | I had difficulty reconciling work during treatment to maintain my company health insurance | Continue working to maintain health insurance |
|  |  |  | The insurance did not cover some of the important expenses for my health/managing the side effects of the treatment | Insurance: limited health coverage |
| **Jones (2024)** | Insurance practices includes health insurance and other types of insurance (disability, life) and encompasses requirements to access coverage and the level of coverage | *“I was probably on the phone with them [health insurance company] once every three or four weeks trying to figure out what it was that they were doing on some of their explanation of benefits resubmitting things. Just it was really painful.”* | I often contacted the insurance company to find out what they were doing with the insurance paperwork | Check insurance |
|  |  | *“Because although I could have COBRA’d, I couldn’t have afforded all my expenses.”* | Even if I could have had health insurance I still would not have been able to pay all the expenses | Insurance: not enough to pay all the expenses |
|  |  | *“But when I was going through this I had to go on disability. My disability insurance tried to screw me. Oh, they tried to mess with how much they were giving me. . . Yes, they tried to short me. Yes, it was thousands of dollars”* | When I applied for disability my insurance company tried to manipulate the amount I was entitled to | Insurance: health insurance dishonesty |
| **Jones (2024)** | Problems with health insurance were not the only driver of financial hardship. Other forms of insurance such as life insurance and short-term disability insurance caused stress and financial hardship. Claims for short-term disability insurance were often denied, introducing an administrative burden to appeal. Overall, generous insurance buffered the best against financial hardship, while administrative hurdles created stress and potentially worsened financial burden. |  |  |  |
| **Jones (2024)** | **Out of pocket care costs** Participants’ out of pocket costs varied from small amounts to greater than $80,000 for one procedure. This included co-pays, deductibles and co-insurance. Sometimes participants were charged for care that would ultimately be covered by their health insurance or received multiple unexpected (surprise) bills; either scenario could create stress. Other out-of-pocket costs included items not typically considered medical care, such as sunscreen or special pillows; new clothes due to treatment-related weight fluctuations; or food costs during cancer care. The line between medical costs and living costs would sometimes blur. One participant reported: | *“But it’s like they give you an itinerary and you’re at [the cancer center] all day basically so you go down to the cafeteria because they have food that you can eat that’s prepared for people that have had transplants very specific. You know specifications and preparation rules. And you just end up putting on a credit card. I mean that was one of our biggest expenses was not being able to go back to the apartment. To like make food? And ending up eating at [cancer center] because it’s not inexpensive so. It’s kind of an interesting rub that the place that’s offering you the food you can eat when you need to eat it is so expensive.”* | I was charged for treatment that was eventually covered by insurance | Lack of transparency in healthcare payment and reimbursement procedures |
|  |  |  | I received some unexpected invoices | Lack of transparency in healthcare payment and reimbursement procedures |
|  |  |  | The food offered at the cancer centre was expensive but I had no choice | Non-medical expenses (food) |
|  |  |  | I had to pay out of my own pocket for the purchase of items that are not considered medical expenses | Financial resources to pay for non-medical items |
| **Jones (2024)** | Costs for healthcare and from treatment side effects. Medical system practices that affect costs includes how procedures are coded, billing practices such as whether a provider will send a bill to collections, locations of clinics and how many appointments a patient need | *“Other types of therapy. You know, acupuncture, that type of thing, and that was all out of pocket.”* | I had to pay for acupuncture and other supportive therapies | Financial resources to pay for supportive therapies |
|  |  | *“The plastic surgery center wanted all of its money up front for the reconstruction surgery.”* | The centre wanted the reconstruction money to be paid before the operation | Advance payment for medical procedures |
|  |  | *“Clothes wise, certainly when I was in chemo, I had to get clothes that were a little bit smaller.”* | I had to get smaller clothes while undergoing chemotherapy | Expenditure on basic necessities |
| **Jones (2024)** | Other costs included healthier food, care for side effects from cancer treatment, and office visits for each chemotherapy appointment. Health insurance characteristics such as deductibles and miscoding of care affected costs of care and whether participants sought care. Methods to reduce costs, such as finding out costs ahead of time to compare treatments or appeal denials of health insurance coverage, were often prohibitively time-consuming. |  | I chose treatment on the basis of the features of my health insurance |  |
|  |  |  | To avoid unexpected costs, I checked the eligibility of costs with the insurance company, but doing so took a long time | Insurance: verifying the eligibility of costs takes time |
| **Jones (2024)** | **Living costs** Participants experienced financial hardship from living costs, including housing (e.g., rent, mortgage payments, yardwork, house cleaning), food, utilities and transportation, and pet care. Sometimes, the struggle to afford living costs could affect participants’ health. One participant mentioned: “I don’t spend a lot, but I probably don’t eat as well as I should because of the cost of food.” While some participants did not struggle to afford living costs, others experienced extreme difficulty paying for necessities partially due to how much cancer and other medical care was covered by health insurance. Recreational activities such as family vacations were often no longer feasible due to increased out of pocket costs and/or reduced income. |  | I did not eat as well as I should have because of the cost of the food | Healthy lifestyle undermined by the cost of food |
|  |  |  | I had difficulty paying for basic necessities and non-essentials | Difficulties in paying for unnecessary goods and basic necessities |
|  |  |  | Health insurance did not cover all medical expenses | Insurance: limited health coverage |
|  |  |  | I perceived a reduction in earnings | Gain reduction |
| **Jones (2024)** | Costs for basic needs and non-essentials (gym memberships, hobbies) | *“And there were things that we didn’t have available to us any longer that. Maybe our idea of our life together just it wasn’t possible, more like travel and bigger purchases.”* | Making trips or having larger expenses was no longer possible | Reduction of expenditure on unnecessary goods |
| **Jones (2024)** | **Dimensions of financial hardship Financial coping** Coping actions ranged from minor rebudgeting to reducing spending on vacations to skipping or delaying healthcare (for cancer and/or other conditions) to delaying retirement and even major decisions about family structure, such as not having children, getting divorced or getting married. One participant stated: “We consider[ed] getting divorced at one point to protect us financially.” Other coping strategies included negotiating with insurance companies or healthcare providers to reduce costs; attempting to fit all care into a single calendar year; or taking less time off work then needed due to lack of PTO. The need to financially cope, and the time and burden required to complete coping actions, was often distressing even for those who successfully avoided financial hardship. |  | We had to reduce our holiday spending | Reduction of expenditure on unnecessary goods |
|  |  |  | We considered divorcing in order to be financially protected | Rearrangement' of family relationships |
|  |  |  | I organised myself to reduce costs but it was distressing because of the load and the time it took to fit everything together | Emotional distress related to payments or organisation to reduce costs |
| **Jones (2024)** | Participants’ actions to afford care and living costs | *“We have not currently delayed retirement, but probably will be doing that.”* | We will probably have to postpone retirement to be able to afford medical and living expenses | Postponing future plans |
| **Jones (2024)** | **Financial consequences** Participants reported well-known financial consequences such as bills going to collections. Other financial consequences included needing to stay married due to finances, not being able to attend cultural practices due to costs (i.e., religious events, weddings), difficulty affording necessities and decreases in credit ratings. Some actions overlapped between financial coping and financial consequences. These included not saving desired amounts for retirement; draining savings or retirement accounts; delaying retirement; and staying with an unwanted job to maintain health or life insurance. Financial consequences were nearly always distressing for participants. |  | I had to stay married for economic reasons | Rearrangement' of family relationships |
|  |  |  | I could not attend cultural events because of the cost | Restriction of participation in social life |
|  |  |  |  | Difficulties in paying for basic necessities |
|  |  |  | I had to postpone my retirement | Postponing future plans |
|  |  |  | I had to keep my unwanted job to maintain my health insurance | Postponing future plans |
|  |  |  | The financial consequences were stressful | Stress caused by financial consequences |
| **Jones (2024)** | Financial events that happen to participants due to trouble coping | *“I was not able to do much savings then for retirement personally.”* | I was not able to accumulate savings for retirement | Inability to accumulate savings |
| **Jones (2024)** | **Financial depression & rumination** Financial hardship led to feeling stressed or upset separately from the other stressors of cancer. This included behavioral symptoms such as crying and physical symptoms such as stomach aches, shaking and tension. This was sometimes described as stress or feeling angry. When reviewing the draft items on financial depression and rumination, cognitive interview participants stated the items made sense and captured patients’ experience. |  | I felt stressed and angry because of economic difficulties | Stress and anger caused by financial consequences |
| **Jones (2024)** | Symptoms of depression due to finances | *“I’m angry or mad because of money.”* | I'm angry because of money | Stress and anger caused by financial consequences |
| **Jones (2024)** | **Financial worry** Participants worried about paying for care and affording health insurance. The lack of transparency around care costs, coverage and timing of bills was often a source of financial worry: | *“I was at my wits end. I was having bills come in and I didn’t know what to do.”* | I am angry about money | Feeling on the edge |
|  |  |  | I didn't know what to do when the bills arrived | Difficulties in paying bills |
| **Jones (2024)** | Co-pays, out-of-network care, whether care would be covered and overthecounter medications were also sources of worry. Financial worry also extended to living costs and maintaining income or employment during cancer care. Participants reported that having generous health insurance or income not dependent on ability to work reduced financial worry. Those who experienced financial worry also experienced impacts on sleep and physical symptoms. |  | Co-payments, health care and treatment coverage, and over-the-counter drugs have been a concern | Concern about co-payments, health care, and the purchase of over-the-counter drugs |
|  |  |  | I have been concerned about the cost of living and maintaining income or employment during treatment | Concern related to cost of living |
|  |  |  | I have been concerned about the cost of living and maintaining income or employment during treatment | Concern related to job retention |
|  |  |  | Having generous health insurance and an income that was not dependent on my ability to work made me feel less worried | Less pre-occupation due to having comprehensive health insurance and a job that was not dependent on my ability to work |
|  |  |  | Financial worry affected sleep and physical symptoms | Financial stress affects well-being and lifestyles |
| **Jones (2024)** | Worry and anxiety due to finances | *“There was a lot of uncertainty about what things would cost.”* | Uncertainty about the cost of things | Uncertainty about the cost of things |
|  |  | *“So, the idea was would I have enough? Vacation or leave or how was I gonna do this without losing my job?”* | I didn't know if I would have enough days off from work to not risk losing my job | Lack of knowledge of labor rights |
| **Jones (2024)** | **Context & individual factors** We examined how community, organizational and policy contexts influence financial hardship. We also examined how individual patient-level differences may influence the experience of financial hardship. |  |  |  |
| **Jones (2024)** | **Positive support** Both formal and informal social support buffered against financial hardships. Formal support included American Cancer Society programs and emotional or informational support from healthcare providers. Even health insurance companies could provide support: | *“The people from Medicaid were really wonderful, I mean they were super nice and helpful, so that made it a lot easier.”* | The people who work for social support have been supportive and made everything easier | People working for social support made it easier |
| **Jones (2024)** | Resources others provide that can buffer the effects of context | *“There was a physical fitness program that they (cancer center) had for those in chemo.”* |  |  |
|  |  | *“My boss even paid for house cleaning and stuff like that and they came to visit and brought food.”* | My boss provided emotional and financial support for some of the non-medical expenses | Emotional and financial support from the employer |
| **Jones (2024)** | Other formal support included social work and nutrition programs. Support from family and friends, providing tangible assistance such as money or groceries, buffered against financial hardship. Coworkers and bosses also provided support. These informal supports often filled the gap in financial needs left by lack of paid time off and insufficient health insurance coverage and could successfully prevent or mitigate financial hardship. However, in some cases, participants needed support but it was not provided. |  | Through formal supports, I had social and nutritional assistance | Formal social support |
|  |  |  | Informal support from family members and colleagues/caregivers helped me mitigate financial difficulties | Economic support of family members and social work environment and help |
|  |  |  | I had finished my paid leave | Job changes affecting earnings |
|  |  |  | My health coverage was insufficient | Insurance: limited health coverage |
|  |  |  | I did not always receive the support I needed | Not getting the support you need |
| **Jones (2024)** | **Social & caregiving context** The support from or need to support partners could influence the degree of financial hardship as did the ages and needs of the participants’ children. The nature of extended family relationships also impacted financial hardship. One participant reported: | *“My family was not functional and so they were not available as resources for me.”* | My family has not been a supportive resource | Lack of support from family members |
| **Jones (2024)** | Some family members had their own illnesses and the patient ended up providing support and caregiving. Patients had caregiving needs themselves while balancing being caregivers to their own family. |  |  |  |
| **Jones (2024)** | The patient’s role as a caregiver to others and the ability of caregivers to support the patient | *“The first time I got breast cancer, I was a single mom with a 9 year old.”* | When I was diagnosed I was on my own and had to take care of my minor son | Being a patient and parent without a support network |
|  |  | *“I was married at the time, so we had two incomes, no kids.”* | We had two salaries in the family | More than one salary in the family helps |
| **Jones (2024)** | **Time & location** Cancer care introduces transportation and time costs that could be substantial. One participant said: | *“I went through [cancer care provider], and their clinics are- at that time-like now they have this big cancer clinic that my daughter went to. So I know the difference. But mine were all spread out at different places. The surgery I had, where I went to chemotherapy.”* | The centers/hospitals where I was treated were scattered in different places, which required time and resources | Location of hospitals where care is received affects FT |
| **Jones (2024)** | Participants observed that the factors that led to financial hardship from cancer had not improved over time, either when comparing their experience to others or comparing initial diagnoses to recurrences. However, changes in the participants’ social and caregiving context could affect financial hardship over time. |  | Financial difficulties have not improved over time | Financial problems that do not improve over time |
| **Jones (2024)** | Cancer symptom burden & financial hardship The side effects of cancer treatment and symptoms of cancer appeared related to the level of financial hardship. For example, lymphedema, fatigue or cognitive symptoms prevented some participants from working at their pre-cancer levels. For others, the need to take off significant time from work negatively affected career trajectories, including job loss, voluntary and involuntary job changes, salary loss and lack of career advancement. Cancer treatment side effects and symptoms also increased the costs of care: | *“The cancer treatment that likely related to having a later procedure did cause some financial hardship, not something super significant. But you know some.”* | Treatment and side effects have caused financial difficulties | The disease and side effects caused FT |
|  |  |  | Symptoms and side effects did not allow me to work as well as before the diagnosis | Illness and side effects affected ability to work |
|  |  |  | Prolonged absence from work caused my salary to be reduced and my inability to have career advancement | Job changes affecting earnings |
| **Jones (2024)** | Treatment of lymphedema led to difficulty working and increased treatment costs. The stress from cancer and cancer treatments could limit participants’ ability to cope with the administrative burdens of accessing disability and health insurance. Some financial effects continued well past active cancer treatment: | *“we’ve had payment plans for medical bills for the last 13 years of our lives.”* | I had a medical expense payment plan that lasted 13 years | Payment of medical expenses that lasts for years |
|  |  |  | The stress caused by the illness limited my ability to deal with the administrative burdens of accessing health and disability insurance. | Stress associated with the impact of illness on the ability to cope with bureaucratic burdens |
| **Jones (2024)** | Location of care centers and changes in causes of financial hardship over time | *“And the bus...when I was doing chemo, I was a little bit paranoid about taking a bus with all those other potentially sick people.”* | I was worried about taking the bus while having chemotherapy. | Constructs without relevant content to answer research questions |
| **Jones (2024)** | **Luck** Multiple participants cited luck as a primary reason for not having worse financial hardship. Lucky circumstances were experienced as either random chance or as good fortune by receiving support, often through reduced administrative hurdles. Chance-related luck included not having worse side effects, COVID-19 providing an alternative reason for accommodations, being able to get all cancer care within a single calendar year or having good income before the cancer diagnosis. Favorable employment and insurance factors were also described as luck, such as working for an employer who decided to provide support and accommodation or having generous health insurance coverage that was easy to access. Support from family, friends, doctors and especially workplaces were cited as luck. |  | I was fortunate not to have financial difficulties | Lucky not to have FT |
|  |  |  | I did not have high financial difficulties because I had a job, a supportive family and work social fabric, and health insurance | Having a job, health insurance and a familiar social network protect against FT |
|  |  |  | I did not experience high financial hardship because I did not have any serious side effects and because the care and treatments were consummated in one calendar year. | Treatment duration and side effects affect FT |
| **Jones (2024)** | Random chance or another providing support that reduced financial hardship | *“I was lucky in that I had a 4/10 schedule.”* | Constructs without relevant content to answer research questions | Constructs without relevant content to answer research questions |
|  |  | *“And I was very lucky in that the insurance I was covered by my spouse at the time was very generous and so took care of most of [the costs].”* | The insurance was very generous and covered most of the costs | Insurance: generous and covered almost the costs |
| **Jones (2024)** | **Individual factors** Participants’ pre-cancer health and money habits appeared to be associated with their level of financial hardship. Pre-cancer health helped protect participants from worse financial hardship. Some people reported tending to save and others tended to prioritize experiences like family vacations, both before the cancer diagnosis and after the diagnosis. Other individual factors included awareness of paid sick leave or work leave policies. Includes financial status before the cancer; pre-cancer financial habits; pre-cancer health; cancer symptoms and treatment side effects | *“[before the cancer diagnosis] We took vacations and saved and paid that off. So I mean, there’s a hardship in the sense of- I mean, we made those personal lifestyle choices to go on vacation or do those kind of things [after cancer diagnosis].”* | I always tried to save money (this protected me from financial toxicity) | Saving (even before diagnosis) protects against FT |
|  |  |  | Knowing the policies of paid sick leave or leave from work protected me from financial toxicity | Knowing job opportunities positively affects FT |
| **Jones (2024)** | **Effects of financial hardship Non-financial consequences** Some participants were unable to adequately rest and recover from the cancer and cancer treatment. Financial coping behaviors, especially those that involved administrative hurdles, took large amounts of time, which further impacted rest and recovery. One participant said: | *“So I’m going to mention it, the amount of time I spent doing really basic things that should have been easy, on the phone advocating for myself”.* | I spent a long time on the phone dealing with paperwork | Procedures for applying for reimbursement of medical expenses/managing paperwork |
| **Jones (2024)** | Indirect effects of financial hardship on participants’ mental, emotional and physical health | *“Meaningful financial support would have meant that I could have the rest time I needed, the recovery time I needed.”* | Having financial support would have given me more time to rest | More financial support affects leisure time |
| **Jones (2024)** | Some participants were pressured to return to work sooner than they wanted by coworkers and did not get sufficient rest. Other consequences included impacts on major life choices, such as forgoing having children. Some participants limited fun activities so paying bills and maintaining health insurance could be a priority. Others reported consequences of financial hardship included stress, ‘sheer hysteria’, worse health behaviors (e.g. less exercise due to energy going to work), physical symptoms and avoidance of finances. |  | I had pressure from the work environment to return to work earlier | Constructs without relevant content to answer research questions |
|  |  |  | We gave up having children because of financial difficulties | Postponing future plans |
|  |  |  | We reduced recreational activities to have money to pay bills and maintain health insurance | Riduzione delle spese per beni non necessari |
|  |  |  |  | Reducing expenditures on unnecessary goods |
|  |  |  |  | Difficulty in maintaining health insurance |
|  |  |  | Because of financial difficulties I was more stressed, had worse lifestyles, and avoided (talking about) finances | Financial stress affects well-being and lifestyles |
| **Jones (2024)** | **Caregiver effects** Participants reported that caregivers, including spouses and adult children, also experienced financial hardship. Some had to take unpaid time off work while others worked more hours to increase income. Others had to quit school or jobs while other caregivers felt locked into a job: | *“He was considering a job transition and he held off on doing that for a while.”* | My caregiver stopped looking for a new job | Pressure on partner/family due to financial stress |
|  |  |  | My husband had to take days off without pay | Pressure on partner/family due to financial stress |
|  |  |  | My husband had to work longer hours to earn more money | Pressure on partner/family due to financial stress |
| **Jones (2024)** | Some couples were buffered from worse financial hardship because both members of the couple were working and they had enough joint resources or the caregiver’s employer provided PTO. |  | My husband and I were working, and we did not perceive any economic difficulties | Continue to work so as not to perceive financial hardship |
|  |  |  | Employer has granted paid vacation/permissions | Practical support from employer/colleagues |
| **Jones (2024)** | Financial hardship experienced by caregivers | *“I’m pretty sure whatever we sold off for assets was mine alone. And so in the vein of, we would have both jointly had that, had it existed in retirement, and we’d still been together, yes.”* | Constructs without relevant content to answer research questions | Constructs without relevant content to answer research questions |
| **Jones (2024)** | **Health behaviors** Cancer diagnosis and treatment often motivated participants to exercise more and improve their diet. Other health behavior changes included taking precautions against COVID-19, sleep hygiene, being more involved in medical care, taking supplements and reducing alcohol use. However, a healthier diet often came with a greater cost that contributed to financial hardship and increased cost of living. The relationship between health behaviors and financial hardship was bidirectional. |  |  |  |
| **Jones (2024)** | Interaction of financial hardship and actions patients take to improve their health | *“Definitely tried to improve my diet after diagnosis, so there is a cost I would say I tried to eat more organic, definitely more local foods.”* | I tried to improve my diet after diagnosis but this came at a cost | Prioritizing one's physical well-being (lifestyle change) |
| **Khajoei (2024)** | **Financial toxicity** The high cost of drugs and treatment, along with reduced work productivity and subsequent loss of income, impose a heavy financial burden on cancer patients, which in turn imposes a unique stress known as financial toxicity [27]. In this study, most patients mentioned financial problems as their most challenging issue. |  |  |  |
| **Khajoei (2024)** | One source of financial problems is the costs associated with cancer care services (e.g., medications, supplies, copayments, and transportation) [28]. Patients who report cancer-related financial problems or high healthcare costs are more likely to avoid or delay their medical care or prescribed medications. This may slow their healing process or aggravate the disease [29]. The participants in this study complained about the high costs of drugs and medical procedures. One participant said: | *I had finished chemotherapy three months ago and was told to start radiation. But I couldn’t afford it, so I gave it up.* | The therapeutic choices I made were dictated by my economic availability | Therapeutic choices dictated by affordability |
| **Khajoei (2024)** | Another financial problem for the participants was that insurance did not cover the cost of some treatments. Obtaining health insurance did not fully protect against cancer-related financial problems. One participant said: | *I am a worker and I have to work hard to improve my wife’s condition. Thank God, most of my wife’s treatment costs were paid by supplementary insurance. However, now the problem is that my wife has to undergo a mastectomy and she is very upset about the change in her appearance and wants to have breast implants. However, because it is considered a cosmetic procedure, unfortunately, insurance does not accept the payment and we are not able to pay the cost.* | I had to work harder to support the costs of my wife's treatment | Constructs without relevant content to answer research questions |
|  |  |  | The therapeutic choices I made were dictated by my economic availability | Treatment choices dictated by affordability |
|  |  |  | My insurance covered a good part of the costs, but not all of them | Insurance: limited health coverage |
| **Khajoei (2024)** | One source of financial distress is reduced income due to loss of employment, missing work, or unplanned retirement [28]. In this study, it was found that some patients were forced to retire and had difficulty with paying the treatment costs. One participant said: | *I am a teacher, and I was forced to retire due to illness. My pension is not sufficient to pay for my medicine and treatment. I really don’t know what to do to pay for the treatment.* | I had to stop working because of illness | Work stoppage |
|  |  |  | My income is not sufficient to meet medical expenses | Insufficient income to cover medical expenses |
|  |  |  | I don't know where to find the money to cover expenses | Difficulties in finding money for treatment |
| **Patra (2024)** | **Financial Challenges** The majority of participants had lost their jobs due to a breast cancer diagnosis. Few had limited insurance coverage, leading to a personal financial burden. All of them had to take out loans at a certain point in time during their treatment to cover the cost. |  |  |  |
| **Patra (2024)** | **Unemployment** | *"When I was diagnosed, a month after that my husband lost his job as he was frequently coming with me to the hospital, so at that time the burden of finance and daily expenses came all together."* | My husband lost his job and the economic burden was felt | Job changes of family members affecting earnings |
| **Patra (2024)** |  | *"I was working earlier, but when I had cancer, I had to leave my job, so the source of income got lost, and we had only one person as the bread-earner, which led to managing my cancer treatment bills and my home," said one participant.* | I had to leave my job due to illness and this led to us having only one income | Job changes affecting earnings |
| **Patra (2024)** | **Borrowed Money** | *A participant said, “After my breast cancer treatment was over, as I had no insurance, I took a loan, so I had to pay them. But after a few years, I got to know that I had cancer in my uterus; at that time, I had to sell my gold to get my treatment done.”* | I didn't have insurance and had to take out a loan to get treatment. | Applying for loans |
| **Patra (2024)** | **Treatment (Before and After)-Related Financial Issues** The cost of specific treatments, such as comorbidities that occurred post-chemotherapy and radiation therapy, was not covered by insurance. The majority of them also stated that you can avail of insurance only when you are hospitalized; however, costs involving preparation before treatment, out-patient costs, and complications post-treatment were not covered, leading to financial strain in their day-to-day lives. Challenges in managing frequent follow-ups and unforeseen health issues arising after cancer treatment also led to financial strain. |  |  |  |
| **Patra (2024)** | **Financial Insufficiency** | *“The only salary that used to come was from my husbands, and when my children were studying, it was a dent in our savings, and at that time they asked me to go for radiation therapy, which I skipped for a while," said one participant.* | I missed a few radiotherapy sessions because the only available salary was my husband's. | Decisions on treatment options based on economic resources availability |
| **Patra (2024)** | **Insurance Disparities** All the participants stated that out-patient costs, implant costs, the cost of plastic surgery, rehabilitation costs, and the cost of side effects that cancer patients face post-chemotherapy were not included in the insurance. Certain medicines, such as Herceptin, were also not included in the insurance scheme, which the patients need for the long term. |  | Supportive care was not covered by insurance | Insurance: limited health coverage |
| **Patra (2024)** | **Insurance Coverage and Disparities** | *A participant said, “If you have side effects, those are not covered under insurance. Certain chemotherapy medications, such as Herceptin, are not covered by insurance. However, these small costs for a longer duration led to financial distress in breast cancer treatment. Also, the rehabilitation costs, such as treatment costs for lymphedema or if you want to buy a prosthesis, all those things are not covered by insurance.” Another said, “Do not discriminate against us based on the disease; treat cancer as any other chronic illness where insurance is covered. We receive calls from insurance companies, but then they shut the phone once they get to know we are cancer survivors.”* | Small medical expenses not covered by insurance have led to financial hardship | Financial stress caused by medical expenses |
|  |  |  | I was treated unfairly by the insurance | Insurance: health insurance dishonesty |
| **Patra (2024)** | **Information and Awareness** Participants reported that insufficient and unclear information was provided about the financial implications of cancer treatment, and they also said there was limited awareness among healthcare professionals about patients' financial distress. There was also a need for financial knowledge for cancer patients. All the participants perceived a need for a financial counselor in hospitals to guide patients and insurance companies and to consider cancer as any other chronic illness. |  | Health professionals are not aware of the financial distress I have felt | Unawareness of financial toxicity issues among healthcare professionals |
| **Patra (2024)** | **Financial Literacy** | *“They don’t have any clue. They are not interested in our financial distress," said one participant. Another participant said, "There is no information on the entire treatment; it’s like you go for surgery, then they give you the estimate, then your next step is chemotherapy, then they give an estimate for chemotherapy, then similar for radiation. Then, in between, you need injections post-chemotherapy, and then you need port insertion. I had to go for two surgeries for that, as no one informed me regarding port insertion. Then I had a chemo leak, and for that, I went for another treatment. Then you do a blood test. And then I had 10 years of hormone replacement therapy (HRT); no one financed me for that.” "There should be a department along with a cancer department. As doctors focus on the treatment aspect and manage the psychological aspect of the patient, this department can focus on giving financial knowledge to the patient regarding the treatment cost. There should be someone who can be an advisor to provide information regarding the treatment cost of the cancer," said a participant.* | I would have needed to be informed about all the expenses I would have to bear | Information need about the economic implications of the disease |
|  |  |  | I would have needed a financial spending consultant | Need to have a case manager of the economic aspects |
| **Ruan (2024)** | **Risk factors associated with financial toxicity** Young women with breast cancer suffered from financial toxicity. Their experience with financial toxicity as associated with sociodemographic factors, financial factors, disease and treatment factors, and personality factors. |  |  |  |
| **Ruan (2024)** | **Sociodemographic factors** Diverse educational backgrounds, family structures, employment statuses, and geographical regions influenced how patients coped with financial toxicity. Participants with higher education levels, with longer working years, who are married individuals with no children, and who are residing in urban areas reported milder financial toxicity. Young breast cancer survivors with higher education levels often displayed a propensity for making informed and prudent treatment choices, resulting in savings from unnecessary expenses. | *I believe in evidence-based medicine, so I didn’t take some expensive nutritional supplements or injections to improve immune system, there isn’t any evidence to prove their effectiveness. (P4)* | I did not buy supplements because there is no evidence that they are effective | Informed decisions on treatment options |
|  |  |  | Young and highly educated women may have less financial toxicity | Young age and high educational level positively influence FT |
|  |  |  | Making informed choices about the type of treatment led me to save money | Informed decisions on treatment options affect FT |
| **Ruan (2024)** | Young women with limited work experience were less likely to accumulate wealth through employment before falling ill, especially those diagnosed at a young age. | *Before I fell ill, I had not worked for a few years. It is definitely not enough to pay for my medical expenses, so my parents must pay for them. (P6)* | I have limited work experience because I am young and could not accumulate sufficient savings to cover medical expenses | Young age affects the amount of savings available for treatment |
|  |  |  | My parents had to pay my medical bills because I did not have the possibility | Economic support of family members |
| **Ruan (2024)** | In contrast to unmarried participants, married women indicated that their husbands shared the financial burden, making them less susceptible to financial toxicity. Nevertheless, women with children faced heightened financial toxicity due to the additional costs associated with parenting. | *Maybe my husband is a little stressed, or maybe it’s because he takes on all the financial pressure, so I don’t feel like I have any pressure. (P22)  After all, my daughter is only more than 5 years old now. I worry about my daughter’s future education and the future expenses of our family. There are certainly financial worries. (P6)* | I shared toxicity with my husband | Pressure on partner/family due to financial stress |
|  |  |  | I am not under pressure because my husband has been under all the economic pressure | Pressure on partner/family due to financial stress |
|  |  |  | I have concerns about the educational future of my children | Concerns for the educational future of their children |
|  |  |  | I'm afraid for my family's future expenses | Concerns for the educational future of their children |
| **Ruan (2024)** | Participants from rural regions encountered more pronounced financial toxicity compared to their urban counterparts. Despite treatment away from home being covered by basic medical insurance, the need for self-payment in advance and the long reimbursement period imposed a significant financial burden on them. | *I paid about half of medical costs by myself because I was covered by the rural insurance system for the first six months. The rural insurance doesn’t cover much money... Sometimes, I have to pay some costs by myself first, then mailed the invoices back home for reimbursement every two or three months. (P23)* | I live in a rural area and have had more financial toxicity | Living in remote areas negatively affects FT |
|  |  |  | Rural insurance cover was only valid for six months | Insurance with limited time validity |
|  |  |  | My insurance system did not cover the full cost of medical expenses. | Insurance: limited health coverage |
|  |  |  | With this type of insurance I had to pay with my own money and was reimbursed after months | Insurance providing only reimbursement of expenses |
|  |  |  | I had to apply for a refund, the procedure was not automatic. | Procedures for claiming reimbursement of medical expenses |
| **Ruan (2024)** | **Financial factors**  Personal and household income, savings, and insurance played a pivotal role in mitigating financial toxicity. Individuals with higher incomes, substantial savings, and supplementary commercial insurance encountered milder financial toxicity. Conversely, those with unstable incomes faced greater financial toxicity. | *I used to open a restaurant by myself, but I stopped opening it after I got sick, so I haven’t had stable income. My parents paid for my medical treatment. Sometimes they had to borrow money from other relatives. (P16)* | I do not have a stable income due to illness | Not having a stable income |
|  |  |  | My working (and earning) future was interrupted by illness | Interruption of the working future |
|  |  |  | My parents paid the medical expenses | Economic support of family members |
|  |  |  | My parents got into debt with other relatives to cover medical expenses | Parental indebtedness |
|  |  |  | Having an income, savings and insurance mitigate financial toxicity | Having economic resources available |
| **Ruan (2024)** | Some participants mentioned that they had individually procured commercial insurance or obtained it through their employers before diagnosis. The insurance payouts provided them with funds to cover medical expenses. | *Because I bought critical illness insurance, I don’t have to pay for the medical expenses myself, and the insurance company can cover hundreds of thousands of them, which can be used for medical treatment. (P14)* | Having insurance to cover medical expenses was important in order not to pay out of pocket. | Insurance providing direct payment of medical expenses |
| **Ruan (2024)** | **Disease and treatment factors** The treatment stages, types, and side effects can all affect financial toxicity. Under different treatment stages, participants perceived financial toxicity differently. During the acute treatment phase, the sense of financial burden was particularly pronounced. However, as they transitioned into the survival phase, their medical expenses became relatively stable and diminished. | *The economic difficulties were mainly due to the large amount of money used in the early stage, which has passed. Now, the main source is wages, which can cover the medical expenses, so there is no difficulty now. (P17)* | The greatest economic difficulties were in the initial phase (acute treatment) | The initial acute treatment phase affected FT |
|  |  |  | Type of treatment, phase and side effects influence the perception of financial toxicity | Treatment, stage of disease and side effects affect FT |
|  |  |  | My medical expenses decreased in the survivorship phase | The survivorship phase is associated with lower medical expenses |
| **Ruan (2024)** | Participants who experienced more side effects and physical symptoms had to pay more for medical expenses to treat their side effects. | *I had a total mastectomy because I had lymphatic metastasis. Particularly tired, prone to fatigue, and now osteoporosis...I have been worrying (about finance) since the end of the acute treatment period. (P23)* | The stage of the disease, the type of surgery and the side effects made me immediately worry about the economic aspects | Cost concerns due to the type of treatment and side effects |
|  |  |  | I paid more because I had side effects that required treatment | Having side effects affects the amount of medical expenses |
| **Ruan (2024)** | Financial toxicity was exacerbated by targeted therapies or long-term medication, primarily because these treatments are often not covered by insurance. | *I’m a little worried about the finances. Targeted drugs not covered by medical insurance are very expensive, probably equivalent to my annual income...Oral targeted drugs will be more expensive. I’m thinking about whether to take them. (P12)* | I am concerned about targeted therapies that are not covered by insurance | Insurance: limited health coverage |
|  |  |  | The cost of the therapies is comparable to my annual income | Work is needed to cover medical expenses |
|  |  |  | The cost of therapies or doing therapies for so long leads me to think about not taking them | Informed decisions on treatment options |
| **Ruan (2024)** | To restore self-image, some participants who underwent radical mastectomy opted for breast reconstruction, exacerbating their financial burden. | *Because I had a bilateral resection, and then had it reconstructed, and a dilator was inserted in the first stage. The patch that I used was more expensive, costing more than 30,000 yuan, this fee is higher. (P4)* | Breast reconstruction is necessary to restore body image but leads to financial toxicity due to its high cost | Restoring the body image has a cost |
| **Ruan (2024)** | **Personality factors** Participants facing the same financial burden felt financial distress differently due to their unique personalities. Optimistic individuals demonstrated reduced subjective financial distress, whereas those predisposed to negative emotions reported heightened financial distress. An optimistic participant maintained hope for recovery, even in the face of substantial out-of-pocket expenses. | *There is not much economic pressure. I am quite confident. My illness will definitely get better in the future. (P9)* | Positive attitude towards the disease positively influencing the perception of financial toxicity | Having a positive attitude affects FT |
| **Ruan (2024)** | Another participant expressed feelings of insecurity and anxiety regarding her financial situation. She was originally the producer of the family but became a consumer after diagnosis, which made her experience financial distress. | *My husband said that I have severe anxiety disorder, I also have that kind of obsessive-compulsive disorder, perfectionism character, and now I can’t fully become the breadwinner of my family. Sometimes, I feel a little unreconciled. (P6)* | Constructs without relevant content to answer research questions | Constructs without relevant content to answer research questions |
|  |  |  | I can no longer support my family economically as I did before the diagnosi | No longer being able to financially support family members |
| **Ruan (2024)** | **Coping resources** This category illustrates the resources to support young women coping with financial toxicity, encompassing both external and internal resources. Sufficient coping resources correlated with milder financial toxicity. |  |  |  |
| **Ruan (2024)** | **External resources** Participants highlighted that support from family, employment, and society was essential to their overall coping with financial toxicity. Family support, in particular, serves as a prevalent and accessible means of both financial and psychological assistance. | *Family support is very important. The support of my husband and the financial and emotional support of my parents, my husband’s parents, played a very big role. (P29)* | The emotional and financial support of close family members and relatives was important to me. | Emotional and financial support of family members |
| **Ruan (2024)** | Some participants also benefited from support provided by their employers, including sick leave compensation or condolence payments. Furthermore, some participants also received support to help them return to work. | *No change in position, no change in salary...The workload is relatively small now; I only need to manage my subordinates, and then I can get off work when the time comes. In the past, I had to work overtime for a long time. (P18)* | The accommodations provided by the employer allowed me to reduce my workload and receive the same salary | Practical support from employer/colleagues |
| **Ruan (2024)** | Few participants actively sought social support, as many felt reluctant to disclose their illness to others. They regarded social support as a final option, utilizing it only if absolutely necessary. | *When I had my operation, I set up a Shuidichou, but I didn’t raise much because my social circle was narrow, and they were all from the grassroots. I raised only 21,000 yuan. (P3)* | I had to organise a fundraiser to support my operation | Economic support from the community |
|  |  |  | The support from my (social) community was there but it was not enough | Economic support from the community |
|  |  |  |  |  |
| **Ruan (2024)** | **Internal resources** Patients’ internal coping resources primarily consist of their psychological traits, knowledge reserve, and survival beliefs. Psychological traits like resilience and independence are commonly observed in young women with breast cancer, who often prioritize self-care over burdening their families. | *They(parents) don’t even know I’m sick now. If I say, they will definitely come to take care of me. But my father is not in good health either, so my mother can’t take care of both of us at the same time. I don’t want them to worry. (P25)* | Constructs without relevant content to answer research questions | Constructs without relevant content to answer research questions |
| **Ruan (2024)** | Furthermore, young women with breast cancer typically have abundant access to disease-related information. This empowers them to make informed treatment choices and navigate both the illness and financial toxicity with greater confidence. | *After illness, I read the literature by myself, then discussed it with the doctor for the optimal treatment method, and adhered to it. I knew how to treat it and understood my own condition. (P6)* | Making informed choices about treatment led me to navigate the disease and financial toxicity with more confidence | Informed decisions on treatment options |
| **Ruan (2024)** | All participants involved in this study exhibited a resolute determination to survive. Even when confronted with substantial medical expenses, the idea of discontinuing treatment never crossed their minds. Survival instinct was their inner strength to cope with financial toxicity. | *The total treatment expenses are about 220,000 yuan. And I borrowed 160,000 to 170,000 yuan. I suffered from this disease, so the money had been spent. Although the expenditures are high, I will not changedrugs. I hope I can achieve best treatment effects. (P27)* | I had to borrow the money for the treatment | Borrowing money for treatment |
| **Ruan (2024)** |  |  | Even though the cost was high I didn't want to change the type of treatment because I wanted to achieve the best effects of the treatment. | Informed decisions on treatment options |
| **Ruan (2024)** | **Unmet needs** Unmet needs played negative roles in the experience of financial toxicity, mainly consisting of information needs, practical needs, and emotional needs. |  |  |  |
| **Ruan (2024)** | **Information needs** Prior to their cancer diagnosis, most young women rarely experienced illness and therefore lacked familiarity with their medical insurance. This informational gap occasionally caused them to miss opportunities for reimbursement, resulting in higher out-of-pocket expenses. | *We didn’t know how to be reimbursed, didn’t know what percentage is exceeded. I think the policy should be more transparent and specific. I think there should be a special person to explain the reimbursement regulations to those who have suffered from a serious disease. This could actually reduce a lot of financial burden. (P28)* | The procedures for claiming reimbursement were unclear | Procedures for claiming reimbursement of medical expenses |
|  |  |  | I would have liked to be followed by a person with specific expertise on reimbursement procedures | Need for a case manager of reimbursement procedures |
|  |  |  | Before my diagnosis I was unfamiliar with my medical insurance | Before the diagnosis I was not familiar with my medical insurance |
| **Ruan (2024)** | Furthermore, participants also highlighted the necessity for healthcare navigation. They expressed the need for guidance in adhering to regular follow-ups and enhancing selfmanagement abilities, which would ultimately help lower indirect expenses like transportation and accommodation fees. | *I really want to have someone specific to set a notification group, or something with a little bit of communication, which could help us to make an appointment for a medical check. That would really save us time and expenditures. (P9)* |  |  |
| **Ruan (2024)** | **Practical needs** Participants predominantly emphasized practical needs, including broadening reimbursement coverage and extending the duration of medical insurance validity. Fulfilling these needs directly contributed to a reduction in their outof-pocket medical costs. | *Drugs outside the medical insurance should be included in the medical insurance as soon as possible, so that patients can substantially benefit. (P12)* | I think the insurance is unfair because it should also cover medical expenses that I have paid myself | Insurance: limited health coverage |
| **Ruan (2024)** | Due to different types of medical insurance, some patients had to make upfront payments for medical expenses before seeking reimbursement. They expressed a strong desire to streamline the reimbursement process to alleviate the financial burden associated with upfront payments. | *I think it would be great if our medical insurance can be used in different places. It would be more relaxed to directly use the medical insurance card for reimbursement. (P23)* | I had to pay out of my own pocket and then ask for a refund | Insurance providing only reimbursement of expenses |
| **Ruan (2024)** |  |  | I would have preferred to use the insurance card rather than request reimbursement but this was not possible | Insurance providing only reimbursement of expenses |
| **Ruan (2024)** | Besides, one-stop inspection service or online inspection appointment service was also urgently needed. Inspection items cannot be completed in 1 day, which increased participants’ visits to the hospital, causing more indirect expenses, especially for those non-local patients | *It takes a long time to take all inspections. It is difficult to make an appointment with an expert. In addition, some inspections cannot be completed on the same day. It occupies a lot of time and money. (P14)* | Check-ups are not organised on the same day and demand time and money especially for those who do not live near the hospital | Timing of follow-ups affects FT |
|  |  |  | I would have preferred to do the checks at once or online to reduce indirect costs | Timing of follow-ups affects FT |
| **Ruan (2024)** | **Emotional needs** Emotional needs were less frequently mentioned by participants, as many of them received substantial emotional support from their families. Nonetheless, patients who were alone in coping with financial toxicity reported a stronger need for emotional support. One divorced patient, burdened by significant financial stress, emphasized that she craved affection more than monetary support. | *Compared to lacking money, what I lack more, I think, is love—the love of spouse and parents. (P23)* | I needed emotional support more because I was alone in dealing with financial toxicity | When alone, one perceives the need for emotional support to cope with FT |
|  |  |  | I am divorced and what I needed most was someone's affection rather than financial support. | When alone, one perceives the need for emotional support to cope with FT |
| **Ruan (2024)** | **Coping strategies** This category demonstrates how young women with breast cancer overcome financial toxicity. They employ four primary strategies: reshaping consumption concept, re-dividing of family functions, re-planning of occupation career, and rebuilding life confidence. |  |  |  |
| **Ruan (2024)** | **Reshaping consumption concept** All participants reported that they reshaped their consumption concept for the sake of treatment and health after being diagnosed with breast cancer. Some individuals adopted greater frugality, while others increased non-medical expenditures. Some participants prioritized treatment costs by cutting down essential living expenses, refraining from luxury and entertainment spending, and minimizing their children’s educational expenses. | *If he (son) wants something that is not related to learning, I will consider whether to buy it, unlike before when he could buy whatever he wanted... As for myself, now I basically don’t buy luxury goods. (P2)* | I have reduced my spending on unnecessary goods since my diagnosis | Reduction of expenditure on unnecessary goods |
|  |  |  | I reduced my child-rearing expenses | Reduction of expenditure on necessary goods |
|  |  |  | I increased expenditure on unnecessary goods | Increased expenditure on unnecessary goods |
| **Ruan (2024)** | Conversely, certain participants who had previously been consistently economical and dedicated to their families gradually shifted their focus toward their physical well-being, endeavoring to lead healthier lives. | *In the past, I gave my family first priority and put myself last. But now it’s different, as if there have been some changes in thinking, why not put myself first? Something I wanted to buy but refrained from to save money before, I feel I can be willing to buy now. (P18)* | I put my own purchasing needs ahead of those of my family | Priority to personal purchasing needs over family needs |
|  |  |  | I focused on my physical well-being and leading a healthier life | Prioritising one's physical well-being (lifestyle change) |
| **Ruan (2024)** | **Re‑dividing of family functions** Young women usually put more effort into work before. However, due to their illness, they have now redirected their attention to their families, assuming greater familial responsibilities. The family function experienced re-dividing. | *(Family role) it has changed a lot. Because I used to be a workaholic, and now I take care of my children at home. I used to have no time for her at work, but now I’m the main caregiver. (P5)* | I put less energy into work | Lower labour investment |
|  |  |  | I focused on the needs of my children | Priority to children's needs |
| **Ruan (2024)** | Conversely, some patients decreased their engagement in family matters and household chores as they heightened their focus on prioritizing their health. | *In the past, I had to worry about all the big and small things in the family, including the elders and the younger ones. Now other family members take on a little more, maybe they want me to focus more on maintaining good health. (P4)* | The management of family aspects was taken over by others so that I could focus more on my health | Practical support of family members |
| **Ruan (2024)** | **Re‑planning of occupation career** Some participants actively or forcibly adjusted their career planning after entering the survival period. Some fortunate participants returned to work smoothly without employer resistance. However, considering their physical condition, they may not aggressively pursue career advancement. | *The intensity of my work now is completely different from before I fell ill…Due to illness, career planning and development will basically be suspended because I will not work hard anymore to seek a job promotion or salary increase. Being competent for the current job is enough for me. (P29)* | I reduced the intensity of my work because after the diagnosis I no longer sought a promotion or an increase in salary | Lower labour investment |
|  |  |  | Due to my physical condition I did not push so hard on career advancement | Lower labour investment |
|  |  |  | It is enough for me to be competent in my work | Lower labour investment |
| **Ruan (2024)** | On the other hand, some participants faced job loss as a result of their illness, compelling them to revise their career plans. Changing to another job or seeking for part-time employment was their strategy to cope with the financial burden. | *Because I am currently working part-time, I can earn an annual income of 50000, 60000, or 100000 yuan. But the job is part-time, so my income varies from month to month. (P6)* | Now that I have a part-time job I have no stable monthly income | Job changes affecting earnings |
|  |  |  | I lost my job after my diagnosis and had to look for another job in order to earn money | Job changes affecting earnings |
| **Ruan (2024)** | **Rebuilding life confidence** Some participants grappled with anxiety and fear after being diagnosed with breast cancer. These negative emotions were triggered not only by the cancer itself but also by unknown financial stress. As their financial situation improved, participants found themselves gaining greater confidence in life. Engaging in discussions about costs with medical staff diminished participants’ uncertainties regarding financial expenses and contributed to their re-established sense of confidence in life. | *I asked the nurse about the costs of treatment. She said that it would take about tens of thousands of yuan. I have enough savings to cover these expenses, so I was not so worried about that. (P25)* | I had enough money to cover expenses so I did not worry | Having economic resources available |
|  |  |  | I was stressed not only because of the diagnosis but also because I did not know the associated costs | Stress associated with lack of information on costs associated with treatment |
|  |  |  | Receiving answers regarding costs from the health personnel allowed me to have more self-confidence | Higher self-esteem associated with information on medical expenses |
| **Ruan (2024)** | Successfully returning to work instilled young women with hope and confidence, enabling them to rebuild their lives and reintegrate into society. | *I was quite worried at that time, but later I found out that the company did not fire me. I have returned to work, and I also have an income, so the worry at that time is slowly weakening. (P29)* | The worry disappeared when I returned to work because I realised that I would not be fired. | Returning to work positively affects economic concerns |
|  |  |  | The worry of losing my job has diminished over time | Changing work concerns over time |
| **Waters (2024)** | **Causes of financial burden** Participants discussed both medical and non-medical sources of financial burden. Among medical costs, the most frequently discussed source were medication costs. One participant shared: | *“Well, that was over $4,000 every two weeks, just that shot. You know, so thank God we had insurance to cover that” (57-year-old, White, MBC patient).* | I had insurance that covered medical expenses. | Health insurance that covered all costs |
| **Waters (2024)** | In addition to medication costs, co-pays and bills from appointments, labs, and scans contributed to financial burden experienced by patients—in part due to the intensity of treatment and the accumulation of smaller costs. | *A 41-year-old, Black, MBC patient shared this sentiment by stating “Our copay used to be like $10, okay, $15, they would cover all my labs. But then, for some reason they don’t, and I have to pay $100. Or, the medication itself is really, really high. So, out of the medication, they leave me with $200 or $300. And after a while, it adds up, because I’m used to having cultures and labs done like every four weeks.”* | I had unexpected medical expenses that should have been covered | Having medical expenses affects FT |
|  |  |  | The cost of medical expenses was very high | High cost of medical expenses |
|  |  |  | Medical expenses piled up because I had frequent paid examinations | Accumulation of medical expenses |
| **Waters (2024)** | However medical costs were not the only driving factors of financial burden reported by participants: | *“As far as the cost, there’s more to it than just the prescription cost” (58-year-old, White, MBC patient).* | My financial burden was not only caused by medical expenses | FT caused not only by medical expenses |
| **Waters (2024)** | Non-medical costs such as travel costs, parking fees, and lost wages due to employment changes often became unmanageable in the context of high out-out-of-pocket costs. Travel costs were a major burden identified by a few participants, because of the distance they had to travel to receive to care was compounded by the frequency of their visits for scans, labs, and doctor’s visits: | *“I have to travel like two hours away when I go… I was coming back like every two weeks” (47-year-old, Black,MBC patient).* | The cost of travel/transportation to do the treatment was one of the main burdens | Non-health care costs (travel) contributing to FT |
|  |  |  | Non-medical expenses to my charge had become unmanageable | Unmanageable non-medical expenses |
|  |  |  | Changes at work have caused me to lose income | Job changes affecting earnings |
| **Waters (2024)** | Another participant shared that regardless of distance traveled, parking fees added up due to long visits stating | *“I could be there sometimes up to four, four and a half hours. And then you’re charged for parking the whole time that you’re there. And I’ve got to do that every month.” (57-year-old, White, MBC patient).* | The cost of parking was my responsibility for all the hours I was in the hospital | Non-health care costs (parking) that contribute to FT |
| **Waters (2024)** | Lastly, most participants shared that after being diagnosed they were overwhelmed by the extent of financial burden they faced: | *“I didn’t really know what it all entailed so, I was in for a shock and I lost a lot.” (44-year-old, Black, MBC patient).* | I did not know the economic consequences of the diagnosis | Lack of knowledge of the cost of medical expenses |
|  |  |  | I was in shock and lost a lot (money?). | Impreparation affecting the amount of FT |
| **Waters (2024)** | **Financial assistance mechanisms** Participants report receiving financial assistance from informal sources such as family and friends as well as formal sources such as manufacturer assistance, charity care, and parking vouchers. Several participants reported receiving informal financial assistance from family and friends. The caregiver of a 65-year-old, White, MBC patient shared this sentiment by saying: | *“Because, you know, her monthly income is so low that she doesn’t even have–she, frankly, does not have enough money for her own expenses. So, therefore, I assist her, and my girlfriend assists her.”* | I received financial support from family and friends | Financial support from family and friends |
| **Waters (2024)** | Further, all participants reported applying for or receiving some type of formal financial assistance. The most common types of formal financial assistance included manufacturer assistance on medication costs, organizational assistance funds, gas cards and parking vouchers, charity care, as well as Social Security Disability Insurance (SSDI), and assistance with costs of living (e.g., utility bill assistance, food stamps). Most formal financial assistance mechanisms were reported to be helpful in reducing patients’ financial burden: | *“The one thing that has helped is the nurse navigator gives me the parking vouchers.” (58-year-old, White, MBC patient), “I received things that I didn’t even ask about, just in sharing, you know, my situation…[social worker] signed me up for things that I wasn’t even aware of. You know, so it was great to open the mail, and get a gift card for [groceries]” (47-year-old, Black, MBC patient).* | Parking vouchers have been helpful to me. | Receive vouchers to pay for parking |
|  |  |  | I received formal financial assistance that allowed me to reduce the financial burden | Receiving formal financial assistance has been supportive |
|  |  |  | After sharing my situation with the social worker, I received some things that I did not expect (in a good way) | Social worker support |
| **Waters (2024)** | However, many participants did report challenges to accessing formal financial assistance mechanisms, particularly in the form of administrative burdens in the application process for programs aimed at alleviating non-medical costs. One 44-year-old, Black, MBC patient shared her experience of not being aware of the needed documentation to receive assistance: | *“I need help with my electricity bill. And, it was kind of like, well, we need proof. They said, we’re sure you’re telling the truth but, we need proof from your doctor that you have cancer. So, I had to leave there, go all the way down to the hospital…wait for it and then come all the way back and get assistance. And, if you don’t get it by a certain day, you miss it that month.” While a 28-year-old, White, MBC* | I had difficulty accessing formal financial assistance | Difficulties in accessing formal financial assistance |
|  |  |  | In order to receive support to pay a bill, I had to obtain medical documentation | Difficulty paying bills |
|  |  |  | Paperwork to access formal financial assistance has a completion deadline | Deadline for paperwork to access formal health care |
| **Waters (2024)** | patient shared that they could not get access to gas cards as frequently as they needed them | *“I think I get a gas card maybe twice a month, usually because either they’re out or, you know, they’re still waiting on some.”* | Formal financial support was not enough to cover the fuel expenses I needed | Insufficient formal economic support to cover non-medical costs (fuel) |
| **Waters (2024)** | Another MBC patient shared their experience of having to wait for SSDI payments to begin and the associated stress | *“And of course, you know, that doesn’t start for like a month or so. So, I have to kind of watch my bills and see when I can pay them, and that just, you know, that’s upsetting sometimes if it looks like I’m going to be late on one because my social security’s not going in in time.” (55-year-old, White, MBC patient).* | I was late in paying my bills because my social security had not yet arrived | Delay in bill payment due to lack of welfare funds |
|  |  |  | I was upset to pay my bills late | Concern about not being able to pay bills on time |
| **Waters (2024)** | **Health insurance and financial burden** When discussing experiences of financial burden, participants commonly mentioned their experiences with health insurance while navigating MBC treatment. Participants’ experiences with health insurance varied substantially but were always intrinsically tied to their financial burden. While few participants had no issues with their coverage: | *“I haven’t had any troubles with it…they pretty much cover everything.” – 41-year-old, Black, MBC patient* | I had no problems with the coverage of my health insurance costs | Health insurance that covered all costs |
| **Waters (2024)** | Others reported the amount their insurance covered to be inadequate. This sentiment was shared by a 60-year-old, White, MBC patient: | *“Well they’re paying it but like I said, they don’t pay much. It’s just—it’s ridiculous to have two insurances and still have to pay lots of money. It’s not, as you know, it’s not cheap. And I get a bill every couple of weeks from the hospital and the physicians, of what I have to pay.”* | The insurance company did not pay enough | Insurance: limited health coverage |
|  |  |  | Although I had insurance, every fortnight or so I got a bill from the hospital to pay | Insurance: limited health coverage |
|  | Further, some participants reported substantial administrative burden due to having to constantly contact their insurer to get their care covered. One 58-year-old, White, MBC patient shared their experience with denials by saying: | *“I had to get back on the telephone because you know they had denied it first time and I basically had to advocate for myself and let them know, hey, I’m a person who previously was diagnosed with cancer and now they think the cancer has spread. So how can you refuse to pay for this?”* | I had to defend myself against insurance companies | Insurance: the need to defend oneself against insurance |
| **Waters (2024)** |  |  | I frequently had to contact insurance companies to get my medical expenses paid | Fighting against insurance companies to obtain one's rights |
| **Waters (2024)** | Another participant reported constantly getting denials due to their insurer believing they were double covered—they were not: | *“They kept trying to say I had insurance through my husband, six or seven years ago. And I told him you know “He was not working, there’s no way I had insurance.” And we found an old phone number with his insurance and called them, and they sent us a letter so that we could prove that we didn’t have the insurance. And they finally started covering it, but that was a very stressful–we had to deal with all that” (60-year-old, White, MBC patient).* | I had to prove that I did not have a second insurance for my medical expenses to be paid | Insurance: the need to defend oneself against insurance |
|  |  |  | it was stressful dealing with insurance companies | Stress associated with dealing with insurance companies |
| **Waters (2024)** | At the same time, some participants also reported uncertainty about what their health insurance should be covering and to what extent as well as when co-pays would be collected and how much they would be. One participant reported being unclear about when co-pays are due stating: | *“But there is an inconsistency when you check-in about whether they want a co-pay or not… Because they insist on one, sometimes they don’t, sometimes they don’t even mention it. Sometimes they’re not going to register you unless you do a co-pay, and it’s all the same stuff, which seems a little confusing.” (61-year-old, White, MBC patient)* | I was uncertain about insurance cover and how much the co-payment of medical expenses would amount to | Lack of knowledge of the cost of medical expenses and insurance cover |
|  | A 58-year-old, White, MBC patient reported being unsure how much they would be charged for services: | *“I get this bill on down the line and I’m thinking oh maybe it will be $40 which is a usual copayment amount or $70 if you go to a specialist. Oh, no. Sometimes it’s 100 or 200 some dollars.”* | I expected the co-payment of medical expenses to be lower | Lack of knowledge of the cost of medical expenses |
|  |  |  |  |  |
| **Waters (2024)** | **Cost‑coping behaviors** While participants reported that formal and informal financial assistance and health insurance alleviated some financial burden, there were nearly always excess costs that participants were unable to afford. A caregiver of a 61-year-old, White, MBC patient shared: | *“It’s still something that even with the Charity Care, which is taking on the lion’s share of is stressful and kind of weighs on us both because, you know, we’ll see a bill come in and say, okay, yes I understand that that’s what the remainder is and [example amount] was covered by Charity Care and [example amount] was covered by Medicaid, but we’re not even in the position to pay the meager portion that is left over for self-pay right now”.* | Although I had health insurance and both formal and informal forms of financial assistance, I was still unable to meet the remaining costs | Difficulties in meeting residual costs despite the presence of health insurance and formal and informal assistance |
| **Waters (2024)** | The remaining financial burden experienced by participants resulted in a variety of cost-coping behaviors including worrying about the costs later, attempting to maintain employment, cutting household spending and taking on debt, as well as alterations in medication use. One 28-year-old, White, MBC patient shared that they were not worried about the cost of their care at the moment because: | *“I’ve got a four-year-old and trying to live, I don’t care about the cost at the minute, you know?”* | I was not worried about costs because my only thought was about my young son and surviving | I was more concerned about my son and surviving than the economic aspects |
| **Waters (2024)** | Another participant shared they had to keep working after their diagnosis to afford their care: | *“For me, even though it was really, really hard and I wanted to stop working, but I couldn’t because of the fact that my income counts as well.” (47-year-old, Black, MBC patient).* | Although I would have liked to, I could not stop working because of the salary | Inability to interrupt work |
| **Waters (2024)** | Other participants reported taking on debt or reducing their spending to afford their care, stating | *“I had credit card debt out the wazoo, just trying to keep up with some of my bills. I just refinanced my house. I don’t know if I’m going to be here in 30 years but it doesn’t really matter.” (40-yearold, White, MCB patient). “* | I got into debt to pay medical expenses | Indebtedness |
|  |  | *I’m trying to make sure that I have the money that I need to, you know, go to the doctor and have the basic things that I need for it. So I’m not able to do some things that I would have been able to do before.” (47-year-old, Black, MBC patient).* | I have reduced spending on unnecessary goods to pay for my care and essentials for living | Reduction of expenditure on unnecessary goods |
| **Waters (2024)** | The same participant reported delaying medication refills due to inability to pay, stating: | *“I have had to wait to get things filled because I didn’t have the money.” (47-year-old, Black, MBC patient).* | I had to postpone the delivery of the drugs because I had no money to pay for them | Postponing treatment because of costs |
| **Khazi (2023)** | **Financial distress** The majority of participants in the present study were from lower socioeconomic classes. The family experienced financial hardship as a result of expensive testing and investigations required for diagnosis. Most of them received assistance from family and friends in this predicament. Two participants were more concerned about the costs than the illness itself. | *No … we did not have any money … not even a rupee … I was very much worried about it. At that time, I was not thinking about my cancer … I had no idea how I will arrange the money for tests. How will I be able to spend so much. From where will I get the money … I used to think (Participant 4)* | We have no money, I am more worried about how to support the investigations and examinations than about the disease | More concerned about costs than illness |
|  |  | *We had to take a loan. We are farmers … we don’t have a lot of money … if the rains are good … we get good yield. We borrowed money from relatives and friends … after we used up all the money we had. (Participant 10)* | Our humble work does not allow us to pay all expenses | Low socio-economic conditions |
|  |  |  | Due to our low status we ran out of money and had to borrow from relatives and friends | Applying for loans to family members |
| **Khazi (2023)** | **Stress and uncertainty** While older participants were anxious about their daughters getting married, younger participants worried about their children’s education and keeping their homes together. |  | I am no longer young but I worry about not being able to help my children get married | Different age-related concerns for children |
|  |  |  | I am young and I worry about not being able to guarantee my children's studies and keep the family together | Concerns for the educational future of their children |
|  |  |  |  | Concerns about future family expenses |
| **Kolawole (2023)** | **Financial implication of treatment** In response to the question “how has breast cancer treatment affected your income?” Almost all (19 of 21; 90.4%) of the participants reported that breast cancer treatment has affected their income greatly. Participants reported spending large portion of their income or saving on their treatment. | *" Most of the money I was using for treatment every three weeks affected my business. I spent close to twenty- five thousand naira on the treatment every 3 weeks. I was nearly started begging for alms. I was supported by my siblings. Presently I don’t have a penny for subsequent chemotherapy” (P1, 66 years, widow)* | The expenses I had for treatment took a toll on my business | Job changes affecting earnings |
|  |  |  | My brothers supported me | Economic support of family members |
|  |  |  | I was in the process of begging | Difficulties in surviving economically |
|  |  |  | I had no more money for more chemotherapy | Lack of money to pay for treatment |
|  |  | *“It really affected my finances, I cannot be recounting my ordeal, I look for money from any avenue in order to continue with my treatment. Even to carry out test it is difficult, but I believe that everything would be better” (P3, 65 years, Married)* | I looked for money from any source to be able to continue treatment | Searching for resources from any source |
|  |  |  | I felt reluctance to talk about the financial situation | Reluctance to talk about finances |
|  |  | *“Yes, it has affected the family finance badly. I cannot run my business at the moment which means we only have my husband’s income for the family. So, I took unexpected loan for the treatment and am paying back without making profits on it” (P8, 46 years, Married)* | My illness has negatively affected the family budget | Concerns about future family expenses |
|  |  |  | The only source of income is my husband's because I cannot run the business | Burdened by knowing your partner/family member is the only financial support for the whole family |
|  |  |  | I am repaying the loan money I asked for treatment without being able to earn | Indebtedness |
| **Kolawole (2023)** | **Effect on functionality and social relationship** This sub-theme explains how their experiences have affected their functionality and their social relationship with friends, family, neighbors, and others. Majority (16 of 21; 76.2%) of the participants affirmed that their functionality as daughter, sister, mother, wife, friend, career women or members of the society had been affected by the nature of their health condition. Some responses are as follows: | *“It brings fear; I don't have time to cater for my children. I cannot provide for my children. I failed in my responsibilities to them. it makes me to be sad and discouraged... (sobbing)” (P21, 64 years Married)* | I can no longer provide for my children | Feeling that you do not fulfil your role in your family anymore |
|  |  |  | I feel sad and discouraged | Sadness and loss of courage due to the financial situation |
|  |  | *“The weakness often experiences each time of treatment could not allow me to do home chores. I am a teacher I cannot go to school because of my physical changes, and it affects me a lot, though I am being paid my monthly salary but there are some benefits I am deprived of, such as gifts from parents and lesson money (allowance) shared by participating teachers” (P10, 34 years, Married)* | The weakness caused by the treatment prevents me from doing domestic activities | Constructs without relevant content to answer research questions |
|  |  |  | I could not continue working due to physical changes | Job changes affecting earnings |
|  |  |  | Although I continued to receive my salary by not going to work, I did not have access to certain benefits | Job changes affecting earnings |
| **Lee (2023)** | **Financial toxicity** Participants discussed both the financial impact and distress of breast cancer treatment with few differences noted between the four subgroups, including those with intersectional identities. Findings represent all groups except where noted. The women discussed their experience with financial distress in two distinct timeframes: short-term and long-term FT. The immediate financial impacts from the time of diagnosis, through surgery and the early stages of primary/active treatment (e.g., radiation, chemotherapy) (hereafter labeled short-term FT), differed from financial impacts of prolonged or maintenance treatment (e.g., endocrine therapy) (defined as long-term FT). |  | The financial impact resulting from the disease is very different when considering the early, active treatment phase from the later, long-term phase, which includes maintenance treatment | The financial impact of the disease varies according to the stage of the disease |
| **Lee (2023)** |  |  | The financial impact from the disease is very similar across groups (women <40 years old, women from colre, women from rural areas, and Medicaid beneficiaries) | The financial impact of the disease appears not to vary among different groups of patients |
| **Lee (2023)** | **Short‑term financial toxicity** The financial impact of active breast cancer treatment on study participants included both direct medical and nonmedical costs, as well as indirect costs, all of which contributed to participants’ experience with short-term FT. We characterized four themes around short-term FT, as described below, with additional comments from patients illustrating these themes presented in Table 2. |  | The disease has a short-term financial impact involving both direct costs (medical and nonmedical) and indirect costs. | The financial impact of short-term illness concerns direct costs (medical and nonmedical) and indirect costs |
| **Lee (2023)** | **No concern about cost/“I just want to live**”  After the initial shock of their breast cancer diagnosis, participants discussed feeling overwhelmed and emotional, with these feelings asked about primary treatment decisions and direct medical cost, participants shared that their focus was on getting well. Many participants talked about doing anything to live, regardless of the financial cost. | *For me… cost didn’t come up in my mind. Whether I’m gonna live or die was my main concern, I ain’t gonna lie. I had healthcare insurance. I wasn’t worried about out-of-pocket costs. I wasn’t worried about finances at all…. like costs was not something that was on the front end of my mind. I could care less. I wanted to live, I was just more concerned like, what can we do? I want surgery now. (Black patient)* | The financial aspects were my last concern. My concern was whether or not I would survive. I didn't care how much I would have to pay. | I was more concerned about survival than the economic aspects |
|  |  |  | My health insurance covered part of my expenses. | Insurance: limited health coverage |
|  |  | *“I just wanted to make sure that I got the best treatment that I could get. And so, honestly, I didn’t [factor in the costs of treatment]. Like, at that point I really wasn’t thinking about like the financial impact of it.” (Rural patient)* | I wasn't thinking about the economic impact at that time, I just wanted to get the best treatment | I was more concerned about getting the best treatment than the economic aspects |
|  |  | *“I didn’t really care about the costs, I really just wanted to be well. I work for the state…, so I have pretty good insurance. So, I guess that really wasn’t a concern.“ (Black and rural patient)* | I wasn't thinking about the economic impact at that time, I just wanted to get the best treatment | I was more concerned about getting the best treatment than the economic aspects |
|  |  |  | My health insurance covered part of my expenses. | Insurance: limited health coverage |
| **Lee (2023)** | **Transportation and childcare** Participants had direct nonmedical costs in addition to the cumulative medical expenses of surgery, chemotherapy, and/or radiation. For example, patients shared concerns about transportation to and from treatment and added childcare burdens for those with young children. Although transportation was an issue for many, regardless of where they lived, those who lived furthest from the medical center expressed that the daily trips, often for a month or more, quickly added to their expenses and distress. | *The transportation costs is pretty big for me. I live an hour and 45 minutes away so, I mean gas can get pretty pricey. Like, especially when I did radiation because I was there five days a week, for like six weeks.(Rural patient)* | I faced significant transportation expenses during the active treatment period | Major nonmedical costs (transportation) |
| **Lee (2023)** | For some participants, especially those 40 years and under, the cost of treatment also included the cost or stress of finding additional help for childcare. | *You’re coming from two hours away…. I mean at the time we were … paying an after-school program to watch the kids until we got back. (40&under patient)* | Living far from the hospital has forced us to incur expenses for an after-school program to look after my children until our return | Increased spending on basic necessities |
| **Lee (2023)** | Another woman similarly reported how multiple direct non-medical costs exacerbated each other and contributed to her stress: | *So, sometimes food, because I didn’t always have money to cover for food, and I do have two children at home. Gas to put in the car. And one of my kids was in daycare at the time, and I was paying for daycare, and I had to apply for a scholarship … for them to help assist with that as well. (Rural, 40&under, and Medicaid)* | Sometimes I didn't have enough money to buy food or gasoline | Lack of resources for basic necessities |
|  |  |  | I needed to apply for a subsidy to pay for the nursery school expenses of one of my children. | State financial support to pay for children's educational expenses |
|  |  | *“My mom and dad would loan us $20, $30, to get us through. We had a little bit of support here and there, but there was times where I just couldn’t make my appointment because I didn’t have the gas to go; I’d have to change my appointment day.” (Medicaid and 40&under patient)* | I had to borrow money from my parents and people close to me. | Applying for loans to family members |
|  |  |  | I had to miss appointments because I could not afford the gasoline to go | Skipping medical appointments because of non-medical costs (gasoline) |
|  |  | *“No, the hardest part for me was transportation. So, it cost a lot because of all the treatments, when you go in, just plan on being there all day.” (Rural, Medicaid, and 40&under patient)* | I faced significant transportation expenses during the active treatment period | Major nonmedical costs (transportation) |
|  |  |  | I had to miss appointments because I could not afford the gasoline to go | Postponing treatment because of non-health care costs |
|  |  | *“And then my sister would come up and help with the kids as much as she could, and she was three hours away at the time. So, I know that was a lot on her.” (40&under patient)* | I had to ask a lot from my family members, to take care of my children when I was doing treatments | Emotional and financial support of family members |
|  |  | *Oh yeah, I had several people take off work to take me to chemo. And then my daughter’s dad took off a month when I had surgery to stay with me and take care of the baby and all that. (Medicaid and 40&under patient)* | Emotional and financial support of close persons | Emotional and financial support from people close to you |
|  |  |  | My former partner had to take time off work to take care of me and my son | Dependence on family members financially |
| **Lee (2023)** | **Changing work and living situation** Beyond direct medical and non-medical costs of treatments, participants discussed needing to make accommodations to receive treatment. These changes had economic repercussions that affected women’s ability to work or work as many hours as they could previously. For instance, one participant on Medicaid noted: | *“So, it was really scary like that for about 18 months with having to take off a month here or two months here…”* | I needed to take several long layoffs from work, and that scared me a lot | Concern related to prolonged work absence |
| **Lee (2023)** | For some individuals, receiving treatment meant changing their work and living situation, for example, returning to live with family who supported them emotionally, physically, and economically through treatment. | *I was planning to stay abroad for a few years and work there… I had no intention of going back to Ohio. And so that was, … very much a left turn…. I was interviewing [for new jobs] and it was just like very exciting. And then I’m back in my childhood room; I need to ask to borrow the car… Yeah, that it was very, very shattering in many ways…. I feel so lucky. But just from a lifestyle perspective, it felt like the rug has been pulled out from under me. (40&under patient)* | I had to disrupt the plans I had made for my future professional career and return to being financially dependent on others | Reviewing the future of work |
|  |  |  | I had to disrupt the plans I had made for my future professional career and return to being financially dependent on others | Loss of financial autonomy |
|  |  | *“I think I’m a little probably unique to most women. I’m a truck driver by trade. So, my income is fairly good. I am single, and I own a home by myself, so I didn’t have a lot of money in savings. With the chemo and the radiation, I had to work a lot less than what I’m used to. But because I have a decent income annually, I really wasn’t able to get any financial support at all….So that was a big year to my livelihood.” (Black patient)* | Illness has put me in a position where I can work and earn much less than before | Job changes affecting earnings |
|  |  |  | I have not been able to get financial supports, and this has had a major economic impact for me | Lack of financial support |
|  |  | *“Living with my parents, quitting one job, quitting another job later. Kind of not being able to apply for jobs because of this or that treatment. These are the, you know, side effects, fatigue, everything.” (Medicaid and 40&under patient)* | Illness has made it difficult for me to hold down a job | Job changes affecting earnings |
|  |  |  | I had to go back to being financially dependent on my family | Dependence on family members financially |
| **Lee (2023)** | **Long‑term financial toxicity** As patients progressed in their cancer journey from surgery and primary treatment to prolonged treatment and follow-up, such as regular surveillance and endocrine therapy, the financial burden of breast cancer treatment was often compounded. Patients described how their distress grew as the burden of the medical, non-medical, and indirect costs increased. We characterized five themes around long-term FT, as described below, with additional verbatim comments from patients illustrating these themes presented in Table 3. |  | Having passed the primary treatment phase and entered the maintenance and follow-up phase, the financial impact was often even greater, between direct and indirect costs | Higher economic impact in the maintenance and follow-up phase |
| **Lee (2023)** | **Never‑ending medical costs** Direct costs continued beyond the early treatment phase for many patients, especially for those needing annual follow-up testing and prolonged therapy. Even though participants expected the early medical expenses, they did not anticipate these enduring costs, and some reported they needed to modify their treatment plans because of the unexpected expenses: | *Yes, I actually have [modified treatments], especially with these follow-up appointments. I’m curious if they are very needed, you know, because it’s accumulating on the inside, on the back side of me owing this balance. So, my cancer has been treated, you know, you got your follow-up appointments, but it’s kind of like expensive. You know, it’s building up. (Black patient)* | The expenses for follow-up treatments are very expensive, and I wonder if they are really necessary | High economic impact for treatments in the follow-up phase |
|  |  |  | The treatment choices I have made have been dictated by my affordability | Therapeutic choices dictated by affordability |
|  |  | *“I have over 5,000 dollars in balance. I never expected to get cancer, to have to even save this type of money to pay this type of bill. That part is very frustrating because I have a balance and it messes with your credit, I mean with your future, if you want to get a house. So, I find it to be a little frustrating that this is something that I couldn’t control, yet I’m responsible and stuck paying for it, for the treatment for my life.” (Black patient)* | The illness has led me to face expenses that I had not planned for, and it has been very frustrating | Lack of knowledge of medical costs |
|  |  | *“I think insurance is the hardest thing because you have to pick plans where you have to have a lower deductible because of my chronic illness, and so it’s so much out of your paycheck all the time. So, like you’re working just to pay for your health care. And now that I don’t have 33 dollars left over a month and we don’t qualify for any help because my husband makes too much money.” (Rural patient)* | My medical insurance is very expensive and everything I earn goes there | Earnings covering health insurance costs only |
|  |  |  | I have not been able to get financial supports, and this has had a major economic impact for me | Lack of financial support |
|  |  | *I pay, right now it’s up to $165 a month, and that won’t be paid off until the summer. I mean, and it’ll keep growing. I’ll get another, well come January, we’ll just keep going, just keep adding to it. It’s never ending, it’s never ending. I’m not poor enough to get any assistance, so we just, I just pay it. (Black patient)* | Lack of Follow-up treatment expenses are very expensive and continue to grow financially | High cost of follow-up treatments |
|  |  |  | I have not been able to get financial supports, and this has had a major economic impact for me | Lack of financial support |
| **Lee (2023)** | **Growing debt** Some of this ongoing care resulted in increasing debt to the medical facility and to others, like credit card companies. For those making monthly payments, the accumulation of expenses from ongoing therapy added to what was owed. For example, one patient mentioned maxing out her annual deductible yet still having to make monthly payments: | *“I was not able to pay my bills, any of them, like my rent; I had car insurance. My mom, my sister, my brother came together, and they ended up paying my bills, but there was still something that I had to let go. Like I had credit cards that I wasn’t able to pay. "(Black, Medicaid, and 40&under patient)* | My family had to shoulder the expenses I was unable to pay, such as my utility bills and rent | Dependence on family members financially |
|  |  |  | I had to give up things [not medically but e.g., having a credit card] because I couldn't afford them financially | Waive/Remand the purchase of services. |
|  |  | *“I was paying $75 a month on my current bill that I have. But then it just got too bad that, just me working from home, it was the only thing that kept us going, you know what I mean? And I couldn’t afford to, we couldn’t afford it no more. And so, we had to stop paying. Right now, I mean, it got to point where we almost got evicted and it’s just, it’s been hard. It’s been a lot of stressful days.” (Black patient)* | I got to the point where I could no longer pay my expenses with what I was earning, and now I'm in danger of being evicted | Not being able to meet the costs from their own earnings |
| **Lee (2023)** | **Bankruptcy** Unfortunately, for a few individuals, the extreme degree of growing debt led them to contemplate or file for bankruptcy. | *So, during that process, I was forced to file bankruptcy; I have no money to pay up like that…For me it got costly, because when you say it cost me $150,000 to go through breast cancer and my cost is $40,000 out of that $150,000. That’s a lot for [my employer’s insurance] and it’s a lot for me. (Black patient)* | I was forced to file for bankruptcy/bankruptcy [seems to be something you can apply for even if you are an employee, because she has a boss] | Declaring bankruptcy |
|  |  |  | The expenses I incur exceed those of my employer's insurance | Insurance: limited health coverage |
|  |  | *“So basically, I just did the best that I could to hold on and I thought that once I got back to working full-time, I would be able to dig myself out like usual, and it just took a little longer than what I anticipated. It was a lot more stressful, you know, just trying to catch up everything. So, I decided to file bankruptcy.” (Black patient)* | I was forced to file for bankruptcy/bankruptcy [seems to be something you can apply for even if you are an employee, because she has a boss] | Declaring bankruptcy |
|  |  |  | I thought I could recover financially once I went back to work full time, but it took much longer than expected | Declaring bankruptcy |
|  |  |  | I thought I could recover financially once I went back to work full time, but it took much longer than expected | Difficulty in recovering the money spent |
|  |  | *“Knowing that there’s a net to catch you if you need to rely on someone else or you know, whether that’s a system like, asking for financial help … or it’s you know, people that you trust like your in-laws or your parents. Just knowing that there is that someone was going to keep us from going into bankruptcy for example, right? And I was doing my best also to prevent that from happening. But you know, I just hear all these horror stories about people who get sick and then and then lose everything because they don’t have those resources.” (Rural patient)* | My family network was the only thing that kept me from being forced to declare bankruptcy | Financial support from family members |
| **Lee (2023)** | **Lifestyle changes and economic compromises** Participants reflected on the long-term financial consequences of breast cancer, including changes to their employment situation (e.g., retiring, changing jobs, remaining on reduced hours) and decisions about where to reside (e.g., living with family, not buying a home). The ongoing consequences of breast cancer also changed purchase decisions and their lifestyle, causing additional stress: | *… pretty much being behind on literally every single bill that I could possibly think of medical-wise destroys your credit. So, it’s not like I can go get a loan, you know? …. It has financially destroyed me. I can’t buy a house, you know; I have to rent; I have to. I can’t finance things on my own; I lease my car. It’s ruined my credit. (Rural and 40&under patient)* | I always found myself paying late for every expense | Inability to pay expenses on time |
|  |  |  | I can no longer afford to buy anything | Reducing expenditures on unnecessary and essential goods |
|  |  |  | Illness has ruined my finances | Disease as a cause of FT |
|  |  | *“I’m selling everything, everything I worked hard for in the 30 years we’ve been married; I’m selling everything. Our favorite thing to do is camp and I’m selling my camper. We have to sell our truck. We’re driving a car that has 200,000 miles on it and I hope I can get back and forth. My husband, he has to drive that back and forth to work and you know, you just pray that it has life left in it. And fortunately, it’s paid off. But you just don’t know how much longer it has on it. You just do what you got to do. You just got to sell it all, you just got to get rid of it, to make the bills go away, to pay off everything.” (Rural patient)* | We were forced to sell everything we cared about to meet expenses | one sells one's assets to meet expenses |
|  |  | *“I owe so much in medical bills that I have decided not to purchase a home. Like, it’s overwhelming. Just the amount of cost associated with, with, with health care and cancer.... So, they’re just building up and it’s just the stress of not being able to pay. I’m on a payment plan already, but it’s just the stress. I mean, I can’t. When I have that debt. I can’t go into, I can’t buy a house if I still have this though, this large amount of debt from these medical bills.” (Black patient)* | The large medical expenses I face prevent me from making any kind of financial investment I had planned for the future | Give up/resend future investments |
|  |  |  | I'm terrified that I won't be able to pay my expenses | Fear of not being able to pay |
| **Lee (2023)** | **Future financial uncertainty** Long after the initial treatment course, along with lifestyle changes and compromises, concern about the prospect of unanticipated health costs led to continued distress among participants with limited resources or who had paid off past debt. Participants were hesitant to spend money or look positively towards the future. Their uneasiness about their future health and finances seemed to blend into uncertainty that impacted their current way of living, causing more distress. For some participants, the seemingly never-ending financial impact of breast cancer left them with little hope for improving their situation: | *Long term, I don’t expect to have, I don’t think I’m going to be able to save up for retirement…. I don’t think I’m going to be middle class. I think I’m just going to be able to pay for essentials, rely on my parents to hopefully help me have a living quarter of some kind, and that’s about it. I can’t really access these dreams of having a house in the suburbs, and going on vacation, and having a job with insurance that lets me take time off … I’m going to be lower income forever, just because it’s how it is really. (40&under and Medicaid patient)* | I can never have the future I dreamed of because of the expenses I have to face | Disruption of future plans |
|  |  |  | I will have to depend on my parents' economic resources | Dependence on family members financially |
|  |  | *“Yeah, I send a payment in every month. I got, I’ve got it down to, I think we’re under $3,000. And I could like, pay that probably, but then I get nervous. Like okay, if I pay the $3,000, what if something else comes up?” (Rural patient)* | I can barely cover the expected expenses. I wonder what will happen if an unexpected expense arises | Barely managing to cover expenses |
|  |  |  | I can barely manage to cover the planned expenses. I wonder what will happen if an unexpected expense arises | Concern in case other expenses take over |
|  |  | *“For years I was the breadwinner of our family; I wanted to do whatever it took to live. But then on the flipside, for financial reasons, I just felt like, I don’t know. It’s like, okay, so if I have these two years left, I need to pay whatever I can to live. But on the flip side, I don’t want to go on any vacations, I don’t want to buy anything extra. I want to pay down as much debt as we possibly can. Because if things go sideways, I’ve got to leave my family in a good situation. So, it’s a real razor wire that you’re walking, for cancer treatment, I think. You know, it’s not like, you can’t like bucket list things, like, ‘well, I’m just going to go out, we’re going to go to Disney, and we’re gonna…’ You know, everything was just super frugal.” (40&under patient)* | I have to calculate every little expense and no can afford to have expenses for things that are not strictly necessary | Reducing expenditures on unnecessary goods |
|  |  |  | I want to make sure I don't leave debts in my family's charge if I don't make it | Concern to exhaust debts so as not to overburden the family in case of death |
|  |  |  | I must also ask my family for efforts not to impact the budget granted to me | Dependence on family members financially |
| **Neilson (2023)** | **Unexpected costs** The overwhelming majority of patients endorsed unexpected costs as a significant contributor to financial distress. These included out-of-pocket medical expenses, such as uncovered physical therapy, acupuncture, and mental health services, as well as nonmedical expenses, such as costs associated with traveling (gas, parking, and lodging) or childcare during treatment. One patient stated, “It’s misleading at best to say that you get an estimate when all you are getting is a list of factors that will play into what your final bill is.” Surprise insurance bills, higher than anticipated deductibles, billing errors, and payments due to unrecognized out-of-network providers also contributed to financial toxicity. All the patients endorsed the need for improved financial transparency desiring realistic cost estimates and lamenting unexpected bills. |  | Unexpected costs have a huge impact on financial stress | Unexpected costs |
|  |  |  | it is impossible to have a real estimate of the costs that will have to be incurred | Greater transparency/clarity on costs to be incurred |
|  |  |  | Greater transparencies necessary to have greater transparency on costs and more realistic estimates | Greater transparency/clarity on costs to be incurred |
|  |  | *“We had to meet different deductibles, so there’s just been a lot of outgoing money to medical bills that was not able to be used for anything else”* | There were unexpected deductible expenses that tied up our savings from other expenses | Unexpected expenses that tie up savings |
|  |  | *“The surgeon used a certain anesthesiologist, and they suddenly were out of my network like 2 days before surgery”* | There were unforeseen expenses for medical choices that were not agreed upon and not communicated with enough time for us to arrange | Unexpected medical expenses given short notice so that they can be arranged |
|  |  | *“I see an out-of-pocket psychiatrist.I’m paying like $200 to $300 per session and then during chemo I was going maybe like every 2 weeks” “There are some organizations that can help you pay for travel that I wasn’t aware of before, and I had already gone and gotten a lot of treatment, and just kind of gave up”* | I had to incur incidental medical expenses not covered by insurance | Insurance: limited health coverage |
|  |  |  | No one ever told me that there are organizations that can help you with some expenses, and I only found out too late | Lack of knowledge of financial support options |
| **Neilson (2023)** | **Lost revenue** Interviewees reported lost revenue from missed work or job loss. The majority of patients reported missing work or using all of their vacation days for appointments and treatment. “I had no idea that the cancer was going to take this long and that I’d have to be unemployed this long.” Many discussed the effects of lost income that came from missing work to attend appointments. “It’s half a day for one 15-min appointment.and that’s a half a day’s income that is lost for me.” Some patients could not hold a job due to their treatments and were left with little to no financial assistance. “I don’t know how anybody works during [treatment].shortterm disability support from the government, after you’ve paid in for years and years, while working would have been tremendously helpful.” Patients discussed how helpful it would be to find ways around having to miss time from work for appointments. One patient emphasized, “I really would like to say, if there [were] night or weekend treatments available.I would think anyone would really appreciate that.” |  | Often there has been loss of income due to prolonged absence from work or loss of this | Job changes affecting earnings |
|  |  |  | The time when it would be necessary to be absent from work was not clearly explained, and this greatly affected my economic income | Information need about the economic implications of the disease and available options |
|  |  |  | Being present for a 15-minute visit means being absent from work for half a day, and this has a major impact on my economic income | Medical examination schedule may affect the amount of work and thus the FT |
|  |  |  | It would be helpful to have government financial support for the periods when, during treatment, it becomes difficult to be able to work | Information need about the economic implications of the disease and available options |
|  |  |  | It would be nice to find ways to arrange the visits so that they do not fall into work schedules (e.g., evenings, nights, or weekends) | Job changes affecting earnings |
|  |  | *“As far as financials go, the effects of the hole that I dug, because of not getting my full income. It’s going to take a long time to get out of it. So I still have that to face. It’s going to take years”* | It will take many years to fill the hole in my personal finances caused by the expenses I had to incur | Time to accumulate lost savings |
|  |  | *“So you’re sort of damned if you do, damned if you don’t. If you don’t work during your treatment, you know, you don’t have resources, and if you earn too much or there’s another source of income, then you’re penalized for it”* | If you do not have work during the treatment period, you do not have the economic resources to support them | Having a job during treatment protects against FT |
|  |  |  | If you had a well-paying job, you are penalized during the treatment period because you are not entitled to financial supports, but still you are not able to work and earn as before | Having a job does not allow access to formal financial supports during treatment when work capacity is reduced |
| **Neilson (2023)** | **Altered budgeting** All patients reported altering their budget in order to prioritize medical bills. Many patients reported limiting leisurely expenses by cutting out certain hobbies, such as cancelling golf memberships, vacations, or being cautious of their utility usage. One patient, when asked about areas where she cut down on expenses stated, “Leisure activities, vacations, home related costs, all the things related to my kids, like various technology expenses.” Most had to dive into their savings and use money set aside for future plans to afford treatment payments. “All of my savings were depleted, and I had nothing. I had to sell everything in my house and sell my car.” Some had to withdraw from their retirement accounts to make ends meet. Patients also mentioned changes to their future financial plans, such as no longer being able to afford the home they desired, while two lost their homes. |  | It was necessary to change one's personal budget to support and prioritize medical expenses | Readjustment/changes in finances to support medical expenses |
|  |  |  | Had to give up some family expenses and hobbies to prioritize medical expenses | Reducing expenditures on unnecessary goods |
|  |  |  | It has been necessary to draw on funds set aside for future projects to cover medical expenses | Depletion of savings, assets, pension funds |
|  |  |  | it was necessary to sell some personal belongings (even the car) to be able to cover medical expenses | Depletion of savings, assets, pension funds |
|  |  | *“You minimize expenses entirely because you have to prioritize your health. Treatments and going to doctor’s appointments instead of working”* | it was necessary to limit all expenditure in order to prioritise healthcare costs | Reducing expenditures on unnecessary and essential goods |
|  |  |  | Often, it was necessary to be absent from work in order to be able to attend appointments, and this affected financial income | The time of medical examinations can affect the amount of work and thus the FT |
|  |  | *“In order to pay the bills.to pay the deductible and the out of pocket, I had to take money out of my retirement fund”* | I had to withdraw money from my pension fund to pay expenses | Depletion of savings, assets, pension funds |
|  |  | *“I had to miss life. I Had to miss things with my kids and events”* | I had to give up things that gave meaning to my life and to do things with my children | Reducing expenditures on unnecessary and essential goods |
| **Neilson (2023)** | **Patient recommendations and timing of education** Patients attributed a limited use of financial resources to lack of awareness of options available. Additionally, patients consistently expressed a desire for early exposure to the financial implications of breast cancer treatment and to the potential resources available to alleviate these stressors. Patients also discussed the need to improve how resources are organized and offered. Many recommended creating a patient portal or information packet highlighting the different resources available to assist with different situations. Most felt a need for information at the time of diagnosis, even though some felt overwhelmed initially by the diagnosis. All participants revealed a desire for financial check-ins early and throughout their treatment. One patient reported, “I didn’t know what questions to ask at that first meeting, and I didn’t really understand a lot of what they give you in this packet full of information.” Patients reiterated the importance of being informed of the financial implications of treatment in addition to being provided easy-to-understand options to help manage these challenges. |  | I had difficulties in managing financial resources to a lack of information on the options available to deal with the costs of treatment | Lack of information on available options to address treatment costs |
|  |  |  | It would be helpful to receive clear information from the outset on the economic implications of the therapy and the resources (e.g. financial aid, support funds) to mitigate these problems. | Information need about the economic implications of the disease and available options |
|  |  |  | It would be useful to create more accessible tools, such as an online portal or an information package, that bring together the different options for financial support in an orderly manner. | Information need about the economic implications of the disease and available options |
|  |  |  | It would be useful to receive regular updates on the economic aspect throughout the treatment process | Information need about the economic implications of the disease and available options |
|  |  |  | it is of paramount importance that clear language and easy-to-understand economic options are used, so that informed decisions can be made without additional stress. | Information need about the economic implications of the disease and available options |
|  |  | *“It would be helpful to have somebody check-in.wouldn’t it be amazing if every patient had.like the equivalent of a project manager?”* | It would be useful for each patient to have a case manager | Need for a contact person for financial matters |
|  |  | *“Tthere are a lot of resources, but when you have insurance you just feel like you don’t qualify for anything. And it would be nice.to get a packet upfront of resources”* | It would be useful to have a package with all the supporting economic resources available right away | Information need about the economic implications of the disease and available options |
|  |  | *“I think cancer patients get bombarded with information in the beginning”* | Too much information is given at the beginning, when one is not yet ready to store it properly | Information overload in the early stages |
| **Walton (2023)** | **Economic factors** **Financial security** Not having to worry about financial issues was one of the major protective resilience factors which would have otherwise been a major barrier for some of them. Many of the participants were able to arrange finances for treatment through savings, insurance, or help from family as seen in the verbatim below | *‘My daughter had insurance, 70% was paid through insurance and as my daughters were working and we had some savings, we managed’. (Age: 63, Homemaker).* | Having insurance, savings and the possibility of having working family members helps to manage financial difficulties. | Insurance, savings and work positively influenced FT |
| **Walton (2023)** | **Financial assistance** Participants obtained financial assistance through various means to complete treatment. It is interesting to note that a participant who did not have money obtained help from friends by communicating with them through social media. | *‘I told one of my friends, she sent a message to the group and it went through to all the classmates, one friend said I will pay your bill, I managed with the money sent by friends’.* | Friends used a group message and financial help from them helped me to pay. | Economic support from the community |
| **Walton (2023)** | **Financial barrier** Finance was a major concern for some of them as they did not get financial support from their spouse or children. The inability to arrange finance for treatment deepened their stress and anxiety. | *‘My son takes care of me, he takes me to the hospital but I have to pay the bill. I did not get any support from my husband or children; I still have credit to pay back’. (Age: 68, Homemaker).* | I got support from my son | Support from close family members |
|  |  |  | I didn't get any support from my husband | Concerning going into debts |
|  |  |  | I still have some bills to pay | Lack of support from family members |
| **Aitken (2022)** | **Physical Symptoms** Fatigue not only impacts BCS during chemotherapy but lasts for years post-treatment. Three women reported that they feel like they have less energy, and two others say they have issues with maintaining work-hour requirements. On average, fatigue was worse during treatment, and now most BCS have reported improvements. Participant 009 was one of many BCS who experienced issues returning to work due to fatigue. | *“I would say for the first three years the fatigue is really, really tangible, very hard to, I guess, to sustain things for a long period of time . . . I found working, going back to work, even though it was a couple of days a week at first, were exhausting. Now, I still, even now, I still don’t have the physical, I guess, stamina that I used to have.” (P009)* | Returning to work was made difficult due to fatigue | Difficulties in returning to work due to side effects |
| **Aitken (2022)** | Cognitive changes impact self-confidence and workability. BCS have reported feeling like they can’t remember words and suffer from chemo fog and short-term memory loss. Four of these participants are worried about these cognitive changes impacting their working life. This issue was highlighted by participant 002, who stated: | *“As well, so I think once I got back into that, I felt like my brain came back online, but before that, It’s like I could. Sometimes I couldn’t even find the words to say, and I was worried. I’m like how am I gonna go back to work and have these conversations with families? And you know, I sound dumb pretty much.” (P002)* | I am afraid that my current cognitive problems may impact on my return to work | Difficulties in returning to work due to side effects |
| **Aitken (2022)** | **Health Services Experience** BCS in both the public and private sectors reported financial distress throughout their journey (see Table 5). Out of the 15 participants, only 5 reported not having financial stress during this time. Of these BCS, all five were part of the private health system. Many did, however, acknowledge the steep prices and shock felt over the cost of their treatment, commenting that they were “lucky” to be able to afford treatment. Of the BCS experiencing financial stress, three were in the public system, and five were private. One woman highlighted the stress of having a lack of income on her family: | *“My husband had to take time off because he had to return to his work. The cancer therapy helped us pay half the bill, which they do because of the stress that comes on between a family and then the kids trying to go to school, go to work, and then your husband’s gotta look after the little one, cook, clean and wash.” (P012)* | I feel stressed because of the impact the illness has on family income | Stress associated with the impact of illness on family income |
|  |  |  | The redefinition of family roles has a negative impact on work participation | The redefinition of family roles has a negative impact on work participation |
|  |  |  | I felt lucky to have been able to afford the costs of treatment. | I felt lucky to have been able to meet the costs of the treatment |
| **Aitken (2022)** | There were also reports that post-treatments, due to financial constraints, BCS were not able to access services to improve their QOL. | *“Everything that’s come after it is virtually out of pocket so that I can’t afford to seek the therapy that I actually really need on a regular basis.” (P007)* | I had to pay out of my own pocket/pay for maintenance therapies | Financial resources to pay for treatment |
|  |  |  | The therapeutic choices I made were dictated by my economic availability | Therapeutic choices dictated by affordability |
| **Marshall (2022)** | **Negative cancer treatment–specific medication beliefs that caused concern for cancer treatment** Negative cancer treatment–specific medication beliefs included concerns specific to symptoms, side effects, and drug-drug interactions as well as financial toxicity, and lack of a guarantee that the cancer medication would work. Subthemes and supporting quotes are provided below. |  |  |  |
| **Marshall (2022)** | **Financial toxicity** Cost of the cancer medication and associated treatment was noted as a concern and a barrier to continued cancer treatment. | *“The cost of the medications is concerning even though I have health insurance.” Participant 5 (age 41, 5 years since initial diagnosis) echoed these concerns, “I mean the cost is always a barrier even with conventional treatment and insurance, and just availability of what you can do, those are the two barriers.”* | Even though I have health insurance the cost of the treatment worried me | Concern about the cost of treatment |
| **Marshall (2022)** | Another discussed the stress of cost as she feared postponing her treatment: | *“I certainly did not have $200,000.00, and the stress financially over the payment of that, I would certainly have said we are going to postpone my treatment for a month. I will risk that rather than risk the stress of this amount of money that I am going to owe, but it was after the fact.” Participant 7 (age 66, 6 years since initial diagnosis)* | Concern about the high cost of the treatment led me to postpone it | Postponed or cancelled a medical appointment because of cost implications of going |
| **Marshall (2022)** | Other participants noted issues regarding the transition of insurance impacting the cost of drugs after quitting employment, as one MBC patient shared: | *“We had a couple of issues with some of the costs of some of the drugs. When I got off of my insurance, we had to go on COBRA which was pretty expensive. We did not know that when you sign up for Medicare, you are supposed to sign up for Medicare Part D to get some other stuff so we had to get help initially for some of the drugs because some were $20,000.00 a pop and the insurance would not cover them. The insurance really did not want to cover any oral medication, they would rather pay for you to go in and get IV treatment which seems ridiculous because you spend all that time in the hospital versus taking a pill. So, I’d have to go on and write to the pharmaceutical company or they would pay for one month which would cover my deductible and then I would be able to take it.” Participant 1 (age 59, 9 years since initial diagnosis)* | After quitting my job, I asked for the passage/continuation of insurance | Insurance: maintaining cover after leaving work |
|  |  |  | I didn't know how to fill out the insurance paperwork in order to get full coverage | Lack of knowledge on how to handle insurance paperwork |
|  |  |  | The insurance company would only pay for one way of administering the drug | Insurance: limited health coverage |
| **Prabandari (2022)** | **Early experiences, prior to accessing health care** Costs of medical care were perceived as unaffordable by participants, despite continuing to incur costs from traditional medicine providers with little or no improvement in their symptoms (quote 5). | *“I took alternative medicine … there was no improvement, Ma’am. The practitioner guaranteed this, this, this, but there was no improvement. One of them sold the medicine for one million [rupiah] per package … but it’s pricey.” (Participant 08, 56 years old)* | I have tried alternative medicine, but without the improvements I was guaranteed. And this type of treatment is very expensive | Alternative medicine has come at a cost |
| **Prabandari (2022)** | **Navigating the system to access treatment** Following numerous primary care consultations, a referral to a larger hospital facility occurred for diagnosis by medical specialists (quote 11), followed by further onward referrals to other medical specialists for treatment (quote 12). Multiple delays often took place between each point of access with health providers. Every trip affected and sometimes worsened participants’ physical condition due to exhaustion and discomfort during transportation (quote 13). Participants who had no private vehicle would have to use public transportation. These commuting routines resulted in significant expenses for transportation and accommodation (quote 14). Some participants and family members even lost their income sources, as they had to take leave or quit their regular jobs to attend treatment sessions (quote 15). | *“… When I felt pain, I told my husband to take me to Puskesmas that night. Then he took me to Puskesmas and I got referred to a district-level hospital. Then, I got referred to a provincial-level hospital.” (Participant 02, 53 years old)* | When I needed, before I was taken in, I was shuttled from one territorial hospital to another | Constructs without relevant content to answer research questions |
|  |  | *“The physician at Puskesmas said, ‘Ma’am, you have to … you need to go to the hospital. I will make a referral. Where do you want to be referred to?’ The doctor asked me to choose between several hospitals in Jogja. I chose one hospital (Hospital P). But the doctor (internist) at Hospital P said the lump needed to be removed with surgery. It cannot be treated with medication alone. Medication will not be suitable. Just undergo surgery as soon as possible (the internist referred the participant to a surgeon)” (Participant 03, 64 years old)* | After being redirected from one service to another, I was told that I would have to undergo urgent surgical removal, because my nodule could no longer be treated with medication. | Constructs without relevant content to answer research questions |
|  |  | *“I had experienced bad physical conditions during my trips to the hospital. My haemoglobin level was only 5,8. During the trip, I vomited blood … I came from Klaten [a neighbouring city about 45 min from Yogyakarta] which is quite far. (Participant 06, 41 years old)* | The hospital is very far away, and I felt very ill on the way there. | Constructs without relevant content to answer research questions |
|  |  | *“The transport [travel to the hospital] was far. It costs three hundred thousand rupiahs [equivalent to 30 US dollars] for one trip, and six hundred thousand rupiahs [equivalent to 60 US dollars] for round-trips … for every treatment visit, I had to stay here for 2–3 days, and initially, I spent two hundred thousand rupiahs [approximately 20 US dollars] per night [for the accommodation]. Luckily I had a nephew here who now provided me with room to stay for free with every hospital visit.” (Participant 07, 58 years old)* | Transport to the hospital each time I had to have treatment was very expensive. Each time I also had to stay there for a few nights, again at my own expense. | Non-medical costs borne by the patient |
|  |  | *“My husband was so supportive of my treatment that he had to leave his job. He used to work at the airport, but because of taking care of me, he quit his job and is now working nonpermanent jobs … as he’d need to accompany my visit to the hospital 2–3 times a week, for chemotherapy, routine check-ups, and picking up the medication.” (Participants 10, 46 years old)* | In order to be able to assist me during my treatment, my husband had to give up his full-time job and now does occasional work | Dependence on family members financially |
|  |  |  |  | Job changes of family members affecting earnings |
| **Prabandari (2022)** | Family members maintained optimism to motivate participants to complete treatment programs, alongside physically accompanying them to hospital visits (quote 25). | *“I did the whole treatment process patiently. Sometimes, I feel sorry for my children who accompany me for treatment. I often say I want to quit the treatment so they won’t be troubled. But my children encouraged me to continue treatment”. (Participant 09, 63 years old)* | I feel so guilty for what my children are doing to support me on this path, that I often say that I would like to stop doing the treatments, so that I am no longer a burden to them. | Constructs without relevant content to answer research questions |
| **Prabandari (2022)** | Participants explained that family roles extend into financial support, as families would collect funds to cover expenses, mostly non-medical, throughout the treatment program (quote 26). | *“I am a housewife. My living expenses including meals are fully provided by my children. They cover medical expenses that are not covered by BPJS. Alhamdulillah, they are very caring, taking care of me wholeheartedly” (Participant 05, 55 years old)* | All my medical expenses not covered by the National Health Service and also non-medical expenses are paid by my children | Dependence on family members financially |
| **Agha (2021)** | **Geographical Barriers** The women we interviewed usually visited Larkana for BC diagnosis. During the interviews, they said that LINAR was not up to the mark in terms of BC treatment. They further added that doctors at LINAR often recommend BC patients to visit Karachi – the capital of Sindh – for proper treatment. Karachi is located around 450-500 kilometre away from the villages and towns where these women lived. This longer distance caused serious demographic concerns because the women were poor and less autonomous to make health decisions. They did not have money to afford expensive treatment, transport and living expenses. Consequently, longer distance adversely affected the attitude of women’s families to go to Karachi for treatment. |  | I did not have the money to pay for medical (treatment) and non-medical (transportation) | Lack of financial availability to cover medical and non-medical expenses |
|  |  |  | Long distances affected the choice of whether or not to do the treatment | The choice of treatment also depends on non-medical costs |
| **Agha (2021)** | The fact that cancer related diagnosis and treatment can be sought only in big cities has impacted the access of families in remote areas. The distance of Karachi, which is the capital of Sindh and where all tertiary level quality care is available, was a problem for poor families. This added travel, accommodation and maintenance expenses, which could have been reduced if treatment facilities were available in or close to their own villages and towns. Some of the families had to move to Karachi to overcome the distance constraint and avoid frequent travel, as in the case of 30-year-old Falak. She narrated: | *I have been to LINAR for almost 5 months then my family suggested a hospital in Karachi, and till date I am following my treatment there. Initially it was a major problem while travelling around 450 kilometres to Karachi, but later we shifted our half family to Karachi and rented a house. Now I only travel for my treatment twice a week and visit my home town once in two months.* | I had to drive 400 km to reach the hospital to receive treatment | Incur non-medical expenses (gasoline) in order to get treatment |
|  |  |  | My family and I had to rent a house to get closer to the hospital where I was receiving treatment | Incur non-medical expenses (apartment rent) in order to get treatment |
| **Agha (2021)** | **Financial Barriers** Most of the women in this study had poor socioeconomic background. They and their families had limited resources to survive on. They lived in remote areas and this geographical barrier further increased the financial burden on them to manage the expenditure, if any of the family members was diagnosed with any disease. Financial barriers also appeared as determining factors in the women’s treatment process. Cancer related screening and treatment is expensive and there are no such financial assistance programs to help the needy. The situation is even worse if a woman’s husband has no source of earning or is involved in drug addiction. Treatment cost for BC varies from hospital to hospital and the stage of cancer. On average, the women’s families spent $10,000 to $15,000 on the treatment. This cost went even higher for the patients who were treated at private hospitals. Some families were not able to arrange the required money which forced them to sell their valuables such as property or business. For example, 50-year-old Rabia had to visit Karachi frequently for her treatment. Her husband was unable to meet the cost of the treatment. Thus, he had to sell his agricultural land, which was the family’s only source of income. Some families, however, managed to arrange money while others waited for any external help in the shape of loans. Husbands of the poor women in our sample tried their best in the beginning to arrange some money in order to seek treatment for the wife. This was apparent in the case of Falak and Naheed. Falak’s husband was not earning thus the father-in-law supported her financially. She also received her share in the property from her natal family which was also used for her treatment. In Naheed’s case, the family first tried to seek loans from relatives, but it was not sufficient for the treatment. They then sought a loan from a bank. Others, who did not have any property, had to leave the patient at the mercy of god. Many survivors in our study said that their husbands and in-laws showed less willingness to initiate the treatment of BC because of unaffordability. In this situation, women had to look to their natal families for support. Many women in our sample sought the financial help of their fathers and brothers after their husbands did not initiate the treatment process, such as 37-year-old Ruhi: | *I had been facing tremendous financial constraints while having treatment of this disease. My husband left me at the mercy of my fate. He said that he had no penny to pay for incurable disease. I was greatly upset. My father demonstrated full support to me. My parents were not rich, but they still assured me of their help. My brothers also came to the forefront to support me. They arranged money and decided to get my treatment done from a good hospital in Karachi.* | I had economic problems while doing the treatment | Economic difficulties during treatment |
|  |  |  | My husband did not support me emotionally and financially | Lack of emotional and financial support from family members |
|  |  |  | According to my husband, it is not worth using money for an incurable disease | Lack of emotional and financial support from family members |
|  |  |  | My family members have arranged to support me financially even though they do not have many financial resources | Financial support from family members despite limited resources |
| **Gharzai (2021)** | Regardless of economic, social, and familial circumstances, respondents experienced emotional and financial stress and disruption because of their cancer diagnosis in addition to objective costs, confirming the established framework. Many respondents explicitly discussed the double whammy impact of worrying about finances on top of a cancer diagnosis, confirming the synergistic effect of objective burdens and subjective distress. | *I think that’s the hardest thing for a cancer patient—for me, wasn’t the treatments themselves. It was the stress and the burden of the financial hardship that you go through, the loss of losing your hair and losing your breasts and your eyelashes, and feeling like you’ve lost your identity. Then the financial burden on top of all of it is unbelievably stressful. You feel like it’s never going to end or that you see the light at the end of the tunnel.* | I found the psychological stress ( worry) and economic damage (objective) heavier than the treatments. | Concerns and economic damage caused by the disease |
|  |  |  | in addition to the disease-induced physical damage that created loss of identity for me, the economic hardship made me feel that I was in a tunnel without light | Feeling of despair due to financial stress |
| **Gharzai (2021)** | The synergistic stress of the impact of a cancer diagnosis on objective financial burden along with subjective financial distress that creates the experience of financial toxicity | *…to have that stress all while you’re trying to just to get through and survive, it really hinders the healing process. You lay awake at night and you’re like, How am I going to do this? How am I going to feed myself? How am I going to make sure that I have what I need and that I get the treatments I need and see the doctors I need and get the medications I need and things like that? (31-year-old, White, non–college-educated woman)* | I experienced two types of synergistic stress from my economic problems: the objective one given diagnosis and the psychological stress given by the fear of not being able to support myself | Fear of not being able to support myself |
|  |  |  | Worries about economic hardship hinder my healing process | Financial stress affects well-being and lifestyles |
| **Gharzai (2021)** | **Application of Existing Financial Toxicity Framework** The qualitative themes emerging from interviews confirmed the existing framework developed by Witte et al9 (Fig 1) and identified a novel addition, with supporting exemplary quotes in Table 1. |  |  |  |
| **Gharzai (2021)** | **Objective burden: direct and indirect costs.**  Objective financial burdens experienced by participants included direct and indirect costs. Direct costs included treatmentrelated costs (such as copays and deductibles) and supportive care costs (such as paying for lotions during radiation or wigs during chemotherapy). Indirect costs included loss of income and employment disruptions such as reduced hours, extended leaves of absences, and loss of employment, experienced by most participants. Other indirect costs were related to an inability to perform unpaid duties that had been previously borne by the participant, such as childcare or domestic duties, requiring paid outside help or volunteers. |  | My economic hardship is caused by direct costs(such as co-pays and deductibles) and costs for supportive care (such as paying for lotions during radiotherapy or wigs during chemotherapy) | Medical and non-medical expenses |
|  |  |  | My economic hardship was worsened by indirect costs: I had to reduce my working hours, take extended absences and leaves of absence | Job changes affecting earnings |
|  |  |  | My economic difficulty is made worse by indirect costs: I have had to pay someone to do the things I cannot do at home | Non-medical expenses |
| **Gharzai (2021)** | **Direct costs**  Direct, measurable costs related to cancer treatment | *I was totally naive about it. I didn’t even really think about the cost of care as far as my cancer care goes with copays. […] They’re a specialty for my insurance, so they’re even more than regular copays. Each ultrasound, doctor’s visit, chemo visit, nurse practitioner visit, and nonstress tests, those were all $30 each. It just didn’t even dawn on me how quickly all of that adds up when you’re going into [redacted] on a weekly. I would have like two or three appointments a week. That’s 90 bucks. Then on top of it, it’s gas. You’re there all day, so you need food to eat and parking. (40-year-old, White, college-educated woman)* | I was unaware that for my insurance the co-payments for each visit were a payment because they were considered as specialized ( $30) and how quickly those expenses accumulate (direct expenses) | Lack of information on insurance coverage |
|  |  |  | I was unaware that for my insurance the co-payments for each visit were a payment because they were considered as specialized ( $30) and how quickly those expenses accumulate (direct expenses) | Accumulation of medical expenses |
|  |  |  | I didn't think that expenses (gasoline , parking, eating out) would come to weigh so heavily on my resources | Non-medical expenses (parking, gasoline, food) |
| **Gharzai (2021)** | **Indirect costs** Indirect costs related to cancer treatment, such as income loss | *I was put on six months leave of absence frommy job, which really hurt me—devastated me financially. (50-year-old,race unknown, some college)* | Being on time off work has drained my resources. | Job changes affecting earnings |
|  |  |  | I must have someone (in-laws, relatives, caregiver) to help me full-time with child care. | Practical support of family members |
|  |  | *My mother-in-law and my father-in-law are both retired. They actually care for our little one. They didn’t have to take any time off from that. I know some of the other family members had to help out, juggle around schedules to make sure that we had childcare and somebody to help me out… (39-year-old, Asian, college-educated woman)* | I must have someone (in-laws, relatives, caregiver) to help me full-time with child care. | Non-medical expenses |
| **Gharzai (2021)** | **Subjective distress: material, psychosocial, and behavioral.** Subjective aspects of financial toxicity describe consequences of financial concerns including material conditions,psychosocial responses, and coping behaviors. This component describes additional burdens that patients face beyond direct burdens. Material conditions relate to changes in spending and use of resources. Respondents took on debt, used savings, or paid bills late. Another material impact was related to property or homes. The most important material concern raised by participants centered around maintaining housing and preventing eviction or foreclosure. Participants additionally expressed concern about making car payments to maintain transportation, which was important to attend treatment visits. Psychosocial responses were related to individual perceptions of stress, fears, worries, and distress. Participants reflected on the emotional toll of cancer, with almost all participants endorsing significant anxiety, stress, and worry related to not only their diagnosis but also the financial implications of treatment. Participants acknowledged these emotional responses as an integral component of the experience of financial toxicity. Financial coping behaviors included lifestyle changes and asking for help from family, friends, and social workers (or other ancillary providers). Participants also noted coping behaviors related to maintaining health insurance, such as intermittently going to work specifically to maintain insurance, or seeking other sources of health insurance such as COBRA or disability income. Thus, the themes emerging from this analysis confirm the existing framework. |  | The psychological stress I experienced from economic problems brought material consequences: we had debts, used our savings and paid bills late, had difficulties in maintaining house (risking foreclosure) and car (both in maintaining it and paying the installments) | Indebtedness |
|  |  |  |  | Difficulty paying bills |
|  |  |  |  | Difficulty in maintaining possession of real estate |
|  |  |  | The psychological stress I experienced due to economic problems had psychosocial consequences: I experienced anxiety, stress, and significant worries anxiety i related not only to the diagnosis but also to the economic implications | Financial stress |
|  |  |  | The psychological stress I experienced due to economic problems led to changes in behavior and lifestyle: I had to seek help from family, friends, and social workers, I adopted as a strategy to maintain insurance to return to work intermittently. | Learning to ask for help from more than one agency/person (coping strategies) |
|  |  |  |  | Returning to work to maintain insurance |
|  |  | *I was too sick to get out of bed most days so it was hard to—I think my biggest stress was making the mortgage payment and the car and the car insurance because those were top priority but still have enough to be able to get groceries and gas in the cars and that sort of thing. (43-year-old, White, college-educated woman)* | Economic concerns were paying the mortgage, car and insurance but also being able to get groceries and gasoline | Difficulty in maintaining possession of real estate |
| **Gharzai (2021)** | **Psychosocial response** Psychosocial manifestations of financial distress | *I wanted to stress the emotional and physical toll that it has a person’s body. Again, like I said, the professionals can tell you what the side effects are. That’s physical. They don’t really know about the emotional part, and of course, that depends on the individual and how they handle things. (68-year-old, Black, college-educated woman)* | While people understand the physical part of my problem, they are not familiar with the emotional part, which depends from person to person | Constructs without relevant content to answer research questions |
|  |  |  | The economic worries were paying the mortgage, the car and the insurance but also being able to buy groceries and petrol. | Difficulty in paying for basic necessities |
| **Gharzai (2021)** | **Coping behaviors** The behaviors and coping strategies used to mitigate financial distress, which include support seeking, coping lifestyle, and coping care | *We had to ask for help a lot. My mom really helped and really had to pull family in to help as best I could. My mom’s job, they were so nice. They did a fundraiser for me. That helped us get through a bit. Yeah, people brought us groceries and food. By other people helping bring some of those things in and having that community around to help is what enabled us to take care of some of the things. Some of those other basic needs. (28-year-old, Black, college-educated woman)* | To cope with financial toxicity we had to ask for help from the family | Financial support from family members |
|  |  |  | The community around me did a fundraiser to help me | Economic support from the community |
|  |  |  | The community around me brought you groceries and food | Practical community support |
| **Gharzai (2021)** | **Novel Addition to Financial Toxicity Framework** Patient knowledge and expectations related to treatment costs and burden were identified as a novel recurring theme that did not fit into the pre-existing financial toxicity framework and significantly affected participants’ experiences. Expectations affected both objective financial burden and subjective financial distress and thus patients’ experience of financial toxicity. There was a substantial mismatch between patients’ expectations of what would occur during treatment, both in terms of timeline (length of expected treatment) and ancillary costs (such as out-of-pocket costs or costs related to supportive items) that led to inefficient choices, increasing participants’ objective financial burden. This included actions such as not planning to be off from work for an extended period and expecting that work-related income would return to baseline levels sooner than actually occurred, or not expecting that costs from treatment would meet insurance deductibles sooner than expected. Expectations held by participants affected not only objective burdens as above but also subjective financial distress experienced (Fig 1). The psychosocial component of the subjective domain was frequently noted as affected by emotions, primarily surprise, related to uncertainty of amount of treatment, and ancillary items needed during treatment. The coping behaviors that were needed to get through treatment were also unexpected by many participants, and the material conditions at times represented a stark contrast from daily lifestyle before cancer diagnosis. Participants explicitly stated that having better managed expectations about financial burdens would have resulted in better preparation, both for the objective burdens of cancer treatment by changing expectations for work and savings needed, and for the subjective distress by preparing patients for the emotional toll and behavioral and material changes in lifestyle during treatment. Better alignment of expectations and reality could improve financial toxicity: | *Had I known about the fact that it could get costly [it would have been helpful]. Fortunately for me, I was able to get health insurance, but had I known about the fact that it can be costly—not so much for the medical supplies, but it can be costly because you’re going to be laid up for a long period of time, and you’re not going to be able to physically do the things that you were able to do prior to the diagnosis and prior to the surgery…I could have prepared better, or at least have had a savings account or had some kind of a cancer plan in place…* | Being aware of the illness-induced expenses not only the health expenses but also the incidental expenses (lost income, not being able to return to work as I expected, needing others) I could have dealt with them better, otherwise | I could have dealt better with medical expenses and indirect expenses if I had been aware of them |
| **Gharzai (2021)** |  |  | If my expectations and reality had been aligned I would have suffered less from financial toxicity | Knowledge about costs that protects against FT |
| **Gharzai (2021)** | **Expectations** Patients endorsed uncertainty related to treatment that affected financial planning and subsequently increased both objective and subjective financial distress | *Had I known about the fact that it could get costly [it would have been helpful]. Fortunately for me, I was able to get health insurance, but had I known about the fact that it can be costly—not so much for the medical supplies, but it can be costly because you’re going to be laid up for a long period of time, and you’re not going to be able to physically do the things that you were able to do prior to the diagnosis and prior to the surgery… I could have prepared better, or at least have had a savings account or had some kind of a cancer plan in place… (51-year-old, Black, college-educated woman)* | Knowing the effects of the treatment on me (secondary effects, such as not being able to work) would have made me better prepared for the economic hardship | I could have coped better with medical expenses and indirect expenses if I had been aware of the effects of the treatment |
|  |  | *You know, honestly, I had no idea. I think I had sticker shock every time I saw, though. Every time I saw how much anything was. Yeah, I think honestly, I didn’t really know how much things were going to cost. (28-year-old, Black, college-educated woman)* | I had no idea I had to incur all these costs, each time it was a beating | Lack of knowledge of medical costs |
|  |  | *I honestly thought this would be really quick. I didn’t think anything. I knew I would be off work. I figured I’d probably only be off work for may be three or four months. […] it’s like a domino effect. One thing happens, another thing happens, another thing happens. What really turned out is this, me going back to work since 2017 twice a year. It’s been three years of me just going back to work for a week or two just to reinstate my insurance. (31-year-old, Black, non–college-educated woman)* | I hadn't estimated that I would be so absent from work I thought much less, I was only coming back to restore the insurance | Failure to match work absence expectations with actual expectations |
|  |  | *They’re just more miscellaneous expenses that one doesn’t expect when you get diagnosed with cancer that later you realize, oh, I need this. (49-year-old, Hispanic, non–college-educated woman)* | We do not expect all the expenses secondary to treatment | Constructs without relevant content to answer research questions |
| **Gharzai (2021)** | **Gaps Identified** Participants identified four areas of concern (or gaps in support) that led to subjective or objective financial distress: incorrect treatment expectations, lack of provider conversation, inability to identify resources, and lack of social support. Participant comments and suggestions also pointed to ways to mitigate financial toxicity (Table 2). As mentioned above, participants wished they had been better prepared for the timing and financial implications of treatment. They highlighted the importance of exploring ways to manage cancer treatment expectations such as through decision aids or access to allied health professions (eg, social workers and financial advisors) to help mitigate financial toxicity. Participants indicated that they had few or no conversations with their doctors regarding the impact of treatment on finances or the financial burdens patients faced from treatment: | *“…I can’t remember any of my oncologists saying, ‘Hey, are you financially stable?’ They just wanted to know the physical and mental part…”* | No oncologist investigated my financial problems, only my physical ones | Lack of interest of financial condition by physicians |
|  |  |  | I identify 4 problems that are not addressed in considering my economic problems: incorrect treatment expectations, lack of conversation with health care providers, inability to identify resources, and lack of social support. | Lack of conversation with health care providers, inability to identify resources, and lack of social support |
| **Gharzai (2021)** | Participants largely noted that they wished that the potential financial implications of treatment had been discussed early on in their treatment course to better plan for the financial burdens. A small number of participants did note that this could be overwhelming early on when patients are first diagnosed with breast cancer. Participants noted a significant challenge with identifying resources for financial assistance during treatment, with many participants turning to sources of information outside of the health care system (such as internet searches and charitable organizations). Participants desired more access to allied health providers, such as social workers or nurse navigators, for assistance in identifying resources. However, participants also noted that allied health providers in many health systems are overwhelmed with the number of patients that require assistance, identifying a key area of need: | *“The social workers at those cancer places, they’re so bombarded by different people that they don’t have that time they need with a individual sometimes.”* | If I had known the potential economic implications, I would have handled the economic problems better. | Lack of knowledge of economic implications |
|  |  |  | None of the health care providers told me about these issues and discussed them with me, I had to get information from outside the health care system (from associations, internet,..) | Seeking support from associations/support groups due to lack of health care support |
|  |  |  | I would have preferred to discuss the potential economic problems with the healthcare professionals, but then again, they are bombarded with requests from patients | Need to have a case manager of the economic aspects |
|  | Finally, participants noted challenges in navigating the health care system and identified support groups as an important source of emotional assistance during treatment, advising patients to seek similar sources of support. Many participants reached out to various nonprofit and charitable organizations for support, but many noted difficulties in finding such sources of assistance and used support groups to identify philanthropic organizations. |  | Since this problem is not addressed in the health care system I relied on associations and nonprofit organizations but it was not dsimple to find them and I had to use support groups | Seeking support from associations/support groups due to lack of health care support |
| **Gharzai (2021)** | Gaps Identified: Incorrect expectations about how treatment would affect finances Participant Advice for Patients: Request clarity, as much as possible, on expected costs and timeline | *I wish I would have known what kind of money I would have been expected to pay. With that, that could have been something I could have been saving for. If I would have known something like that and had savings for medical expenses, then that’s something that would have been a big help. (41-year-old, Black, college-educated woman)* | Knowing the costs beforehand I could have handled it differently and saved money | incorrect treatment expectations |
|  |  | *I definitely wish they let me know what I was in for, what to expect… (48-year-old, White, non–college-educated woman)* | I wish I had known what was in store for me | incorrect treatment expectations |
| **Gharzai (2021)** | Gaps Identified: Lack of provider conversations about finances  Participant Advice for Patients: Discuss early that finances can be an issue during treatmen | *With my general providers, I don’t think it was ever really brought up at all. When I first was diagnosed, I would go in and see my breast surgeon and my team, and they would talk to me about the treatment aspects of it. I don’t really think that anything was really mentioned about the cost. (39-year-old, Asian, college educated woman)* | With primary care physicians, I talked only about diagnostics never about costs | Lack of provider conversations |
|  |  | *Right from the very beginning, absolutely, [is the right time to mention financial strain related to treatment]. As soon as you’re diagnosed and you go in there and they give you that packet saying this is what you have, this is what we’re going to be doing next, they should give you the information to say, ‘I know this is overwhelming, but here’s some other information just for you to have. You may not need it, but just in case. We give it to everybody.’ Let them have it because it’s better to have it early than too late. The sooner the better, I think. (43-year-old, White, college educated woman)* | They should tell you with the diagnosis and what they will do with you that you will encounter financial burdens | Lack of provider conversations |
| **Gharzai (2021)** | Gaps Identified: Inability to identify financial resources Participant Advice for Patients: Seek out allied health provider to find resources for assistance | *The nurse navigator helped so much. I was so glad that he actually—because I was prideful and didn’t want to say anything, but I was glad that he pushed me [to get help]. (41-year-old, White, college-educated woman)* | The nurse navigator helped me to ask for help, I out of pride would not have done it | Strategie di coping |
|  |  | *Well, I wish I had known about [the financial advisor] from the beginning because I didn’t find about her until—I probably was halfway through treatment when I found out about her. (31-year-old, Black, non–college-educated)* | I met the financial advisor in the middle of the treatment, it would have been helpful to know him earlier | Inability to identify financial resources |
| **Gharzai (2021)** | Gaps Identified: Inability to identify financial resources Participant Advice for Patients: Seek out assistance outside of medicine through friends and family or philanthropic organizations | *We had to ask for help a lot. My mom really helped and really had to pull family in to help as best I could. (28-year-old, Black, college educated woman)* | We often had to ask for financial help from the family | Financial support from family members |
|  |  | *There’s a bunch of different organizations, and they would help with little things. Like one might help with a car insurance bill. I did research for hours on the computer trying to find what organizations were available for me. That’s kind of how I made it through the second time [I was diagnosed with cancer]. I even got help from my church. My church was able to help me make mortgage payments for two months so just kind of reaching out. (42- year-old, White, college-educated woman)* | I asked for help from many different associations, to pay car insurance but also from the church. | Learning to ask for help from more than one agency (coping strategies) |
| **Gharzai (2021)** | Gaps Identified: Lack of support in navigating the health care system Participant Advice for Patients: Find a support group, get help of allied health professional | *I think I would…definitely [advise] a group or a Facebook page, a website, or something where they can go to talk with other women who’ve been through it. I think you learn more when you connect with other women that are going through it or have been through it. […] Mainly just let them know there’s people out there to help them. (43-year-old, White, college-educated woman)* | Would need a sharing group on social to share info and experiences | Coping strategies |
|  |  | *I wish I would have had a buddy system to be matched up with a fellow, another—I mean, like multiple battle buddies that were going through it. With now going through it, I wish it would have been able to where they could have helped with getting a lot of information out with other resources, and just really understanding the process more… (37-yearold, Black, college-educated woman)* | Would need a sharing and support group to share info and experiences | Coping strategies |
| **Lewis (2021)** | **The temporal tussle surrounding decisions of daily (and future) living** Choices about treatment, care, work and family life could not be disentangled from the anticipated or imagined future of living-with advanced cancer (Kenny et al., 2017; Llewellyn et al., 2018). Participants discussed the challenges of making decisions about how to live in the now, in relation to the largely unknown, but likely contracted, future. This was particularly evident among young women, women who discussed financial difficulties, and women who had been living with metastatic cancer for longer periods of time. What emerged were frequent considerations (and reconsiderations) of how to spend finite time and financial resources, and the consequences of such decisions (e. g., about participation in paid and unpaid work, care responsibilities, dating, housing and travel for everyday life both now and into the future. Take for example the following excerpt from Lorraine, who had recently stopped working due to ill health: | *All those decisions are really hard when you’re in this position because you just don’t know how long you have. If I knew back when I was 49 I was going to still be here when I was 55, I possibly would have done things differently … the big worry is, now I’ve retired, have I plunged us into poverty? We could have done so much more had I stayed and worked for another 10 years. Have I done the wrong thing? … There’s just all this ongoing financial burden and, again, it’s making decisions. If I jump now, am I going to live for another 30 years and be absolutely poverty stricken? I don’t know. In the beginning it was, “Your prognosis is bad … Two to five years is usually what you’re looking at … there’s no choice about it. I don’t know. I try to be a pragmatist and just try to think, “I’m here.” (Lorraine aged 55, Interview 1)* | With inauspicious prognosis I managed my resources in an inadequate way because I survived longer! | Prognosis and resource management |
| **Lewis (2021)** | As Lorraine alludes to, her decisions about continuing work were complicated by ambiguity around prognosis, and the changeable nature of prognosis (see Jain, 2007; Lewis et al., 2020). Several participants described their initial decisions as made in the context of a (particular) prognosis, and the subsequent social and financial implications of living beyond prognosis. Contemplating and foreseeing an imagined future was hindered by the unpredictability of living with an incurable, progressive condition. In practice, the process of deciding how to live in the now, and plan for the future, never unfolded straightforwardly, and was always imbued with emotions. Denise and Amber, both in their early sixties and living with metastatic cancer for ten and four years’ respectively, discuss how balancing living in the now with living in the future (for self and others) was continuously being deliberated and negotiated. | *I’ve got to be a little careful. I want to do all this stuff, but what happens if I do last several more years and I need care, if I need to be put into a high care facility … that is a bit of a concern. I don’t want to leave my husband with nothing. (Denise, aged 63, Interview 1)* | Uncertain timing on prognosis makes me have doubts about how to manage my resources | Prognosis and resource management |
| **Lewis (2021)** |  | *You’re told that you’ve got 12–18 months to live so you’re counting the months thinking, “Oh my god, I may only have nine more months to live” … But when that time comes and I’m still going, I’m still going through treatment, I’m still here, relief took over … There’s always uncertainty in your life. (Amber, aged 62, Interview 1)* | Vivid temporal uncertainty in prognosis | Constructs without relevant content to answer research questions |
| **Lewis (2021)** | Suggestive of the moral responsibility women felt, even in very constrained circumstances or when decisions were imposed on them, participants described their decisions as accompanied by feelings of guilt or anxiety about the possibility of making the ‘wrong choice’ (Bell, 2016). The negative repercussions of these decisions on themselves and others were described at length (e.g., cancer progression, treatment failure, the hastening of death, increased financial burden on family members, the unaffordability of future treatment or care). The following excerpts illustrate the inseparability of treatment decisions from relationships, time and money, highlighting the necessity but also the complexity and difficulty of the notion of ‘patient choice’ as mobilised in any straightforward or linear way (Llewellyn et al., 2018). For instance, Kylie, in her late forties who was actively looking for work and reported financial difficulties, talks about being offered a choice by her oncologist between two treatment options, with a significant difference in cost (one was publicly subsidised, and one was not). The more expensive option was framed as ‘better’ (reflective of a wider market logic) but was prohibitive due to her limited financial resources. Yet she articulates the failure of the treatment as a personal failing, provoking feelings of guilt and worry that her decision may have shortened her life. | *… my husband said, “No, we’ll do it,” and I said, “No, we won’t,” because I’m high-risk. I wouldn’t put our family into any – I’m already a financial burden. I’m not going to make it worse. You know what I mean? The hundreds of dollars already spent this year, especially I’m not now working and he’s just working part-time, it’s a concern nowadays. (Kylie, aged 49, Interview 1)* | Choosing to treat myself and spend the money causes me guilt toward my family: they are an economic burden | Feeling of guilty |
| **Lewis (2021)** | Making the ‘right’ choice was just one of many forms of normativity that shaped decisions about how best to live-with cancer. Being a good patient, and particularly a good mother, was foregrounded. Tammy, a fifty-year-old with two dependent children living with metastatic cancer for five years, articulates the inseparability of decisions to care of self and their sense of care and responsibility to their children. | *When I stopped working it was a very conscious decision. It was because I worked all day every day providing care to others, that I felt I needed to be engaged in self-care at that point. So, things like looking after my diet, looking after exercise, making sure I took up meditation. A friend introduced me to tai chi and so I still practice that every day. I felt like my job then was to be looking after myself, and I still, to some extent, feel that is my main job, to look after myself so I can keep being a good mother to my daughters. (Tammy, aged 50, Interview 1)* | Constructs without relevant content to answer research questions | Constructs without relevant content to answer research questions |
| **Lewis (2021)** | Illustrative of the temporal dimensions of choice, decisions about the future could not be disentangled from the multidimensional complexity of participants’ daily life. This included the interwoven affective and economic aspects of living-with cancer. Sense of duty, morality, and time and resource constraints all shaped decision-making, both within the present moment and in the imagined or anticipated future. This involved engaging in an ongoing process of (re)adjustment to the changing demands and expectations of the present and the future; and how resources were mobilised (and rationed) in an attempt to manage uncertainty. |  | I had to continually readjust my choices to balance economic, family aspects to manage the uncertainty of prognosis | Balancing economic and family aspects according to prognosis |
| **Oshima (2021)** | **Financial Impact Due to Direct Costs for Medical Care** Within the theme of financial impact due to direct costs for medical care, three distinct groups of patients emerged from the study cohort: those with adequate financial resources, limited financial resources, and inadequate financial resources. |  | The authors distinguish three different groups of patients: those with adequate financial resources, those with limited financial resources, and those with inadequate financial resources | Availability of financial resources |
| **Oshima (2021)** | Of the 511 participants included in this study, 313 (61.3%) respondents wrote about financial burden. Of the comments coded under this theme, 180 (57.5%) reflected adequate financial resources to cover all out-of-pocket expenses related to their cancer care. These respondents routinely expressed perceived privilege and gratitude in being able to pay for their care. | *“I amSO grateful that I had such good insurance for my breast cancer treatment. I was able to get treated at a cancer center, and not restricted to a certain hospital such that an HMO would require. After I met the large deductible of over $5,000 my insurance covered most of the costs of all of the treatment. The costs that I did have to pay were manageable for me. I realize that not everyone is as fortunate as I am financially when being treated for cancer”* | Positive role of having good insurance that helps manage illness-related costs and make better care choices. I am privileged and aware of the financial difficulties of those without good insurance. | Insurance: having good health coverage |
| **Oshima (2021)** | Participants with limited financial resources (n 5 99, 31.6%) reported that they were able to pay for all expenses of their cancer treatment but still incurred significant stress in doing so. Many respondents reported that they were faced with a situation in which they had to choose between significant financial burden and their health. In paying their cancer-related medical bills, respondents reported depleting their savings accounts, retirement funds, and borrowing money from friends and family. Despite these negative financial consequences, many respondents with limited financial resources simultaneously expressed gratitude for existing insurance coverage and their survival. | *“For me, the financial aspect of my treatment was incredibly anxiety provoking and, subsequently, guilt ridden. There was no question that I was going to undergo treatment, but we had to use savings to cover all of the costs, ask our families for support, and constantly deal with various hospital/office billing departments”* | Using family savings to pay for necessary treatment costs creates stress and guilt. The need to constantly deal with various offices/departments also creates stress. | Family savings as available financial resources. Need to deal with costs. |
| **Oshima (2021)** | Finally, women with inadequate financial resources (n 5 34, 10.9%) often noted catastrophic financial damage as a result of treatment costs. For these women, breast cancer treatment resulted in irreparable financial damage, such as declaring bankruptcy or losing their home, to pay off medical debts. | *“Because of the gap in my vitae and my age I have found it difficult to find full time professional employment again. I finally got into HUD housing. I have food stamps. I still owe $4,200 for cancer care (both cancers combined having hit the deductible and out of pocket for 2 years running initially, and then in 2014 again with what we thought was a recurrence, right now between insurance—which this year runs $1,119.19/month, no subsidy as I don’t make enough for one, no Medicaid expansion and the likely out of pocket deductible that I will spend this year (anticipated). I will spend close to $18,000 this year on medical, plus around $1,700 on the payment plan for past due bills that go all the way back to 2014. I make less than that a year so have had to spend retirement money to pay. Obviously, I will now be in poverty the rest of my life”* | Job instability and limited benefits are not enough to pay for treatment costs. Due to my limited economic resources I have medical debts and need to use retirement savings to be able to pay. This has generated concern about a condition of poverty that will continue for years. | Job instability and limited economic resources negatively impact FT and create concern for the future. |
| **Oshima (2021)** | **The Financial Arc and Long-Term Impacts** There was a wide range of reported time since diagnosis in this study cohort (Table 1), with around 30% of the cohort diagnosed over 10 years prior to survey completion. This variation meant participants were approaching the topic of treatment costs from very different perspectives, with some women currently undergoing treatment, whereas others were decades out from their initial diagnosis at the time of survey completion. When sorting participant responses that directly mentioned financial impact by time since diagnosis, a financial arc in patient experiences emerged. Around the time of breast cancer diagnosis, women described uncertainty around treatment costs. As treatment progressed, participants reported increasing cancer-related financial burden as deductibles, co-payments, and other expenses accumulated. For some participants, these costs dwindled following the end of treatment. For others, this financial burden persisted for years, sometimes increasing with compounding debts long after the initial diagnosis, representing drawn-out financial arcs. Finally, participants living with metastatic breast cancer had a notably different shape to their financial arc, with increasing self-reported costs over time as participants remained in treatment indefinitely. Most participants noted difficulty in anticipating future costs, with many explicitly reporting stress and fear over unexpected costs associated with potential recurrences or unexpected treatment (eg, chemotherapy and exchange in breast implants). | *“We were in shock when I was diagnosed. We knew we had insurance and we knew once I chose what to do, they would handle everything and we would work out payments on anything they didn’t pay. I knew because I had insurance, I would be able to choose whatever route I wanted to go. I was never told that my breast implants would need to be replaced and I never thought about it until they leaked. In the 18 years since my surgery I have had both implants replaced twice. The cost is very high. We have private insurance with a large deductible, so it is a burden to have such a large expense. Also, with my insurance I always have to pay for the doctor bills myself, including the anesthesiologist. I worry that in 10 years when I need to have my implants replaced again, it will be an extreme burden on our family, considering neither my husband nor I will be working”* | At the time of diagnosis being informed about insurance coverage helps address concerns about treatment costs. Positive role of having good insurance that helps manage disease-related costs and make better treatment choices. Unexpected treatments such as implantation/breakage of prostheses with associated high costs may be necessary over time. This generates concern for the future, both because of the need to use savings and possible change in the family's working conditions creating economic instability. | Role of the care pathway phase on FT. Being informed about costs to be incurred and insurance coverage helps. Concern about costs of unexpected/unplanned treatments. Using family savings as financial resources creates pre-occupation about the future. |
| **Oshima (2021)** |  | *“I am still undergoing treatment, so I do not know what my total expenses will be. Medical treatment in general is very expensive, and copayments for a major illness like cancer are really impactful to a family and household budget. I don’t feel like I have any option to NOT do treatment, as the result would be death. So faced with the options of death or financial hardship, I chose the later”* | Being in the process of treatment creates uncertainty for me with respect to the cost that I will incur. The costs affect the family budget, and I cannot choose not to get treatment because the alternative is death. | Role of stage of care pathway on FT related to lack of information regarding costs associated with treatment creates stress. Forced choice between having to treat and death based on financial availability. |
|  |  | *“I heard rumors at work that I was often passed over for promotions, because they feared that if I got cancer again, they would have to pay me more if I had to go on medical disability. It took me over ten years to finally get a promotion that I had deserved years earlier”* | Difficulty in career advancement at work due to fear of having to pay more for disability in case of recidivism. Need to work harder to get work recognition. | Need to work harder for work recognition. |
| **Oshima (2021)** | **Discordance in Stated Financial Burden** Most participants’ self-report of their financial burden on multiple-choice survey items aligned with the comments included in the open-ended responses. For example, one participant described being financially ruined with no way to recovery after surgery and appropriately selected catastrophic financial burden in response to the survey question “How much of a financial burden did you experience as a result of your breast cancer treatment?”10 Notably, we also observed discordance between the degree of a participant’s stated financial burden and their descriptive financial burden on open-ended response in 15 of 313 responses coded under the financial burden theme (4.8%). For example, one survey participant described owing $40,000 (USD) to her radiation providers, being harassed by the oncologist’s office and a collection agency for bill payment, and developing panic attacks as a result of this stress. However, on the survey, this participant chose slight financial burden. Another participant described anxiety and guilt surrounding the financial aspect of treatment, requiring her to use savings to cover all the costs and ask her family for financial support, but indicated somewhat of a financial burden on her survey response. Yet another participant noted that her family had spent thousands of dollars on treatment in addition to the thousands in lost wages and wrote: ...... This participant chose somewhat of a financial burden on her survey response. | *“I worry that a re-occurrence would bankrupt our family, and in that case, I would probably forgo treatment despite being a relatively young person”.* | Fear of relapse from not being able to pay the high costs. Inadequate availability of economic resources driving treatment choice (not getting treatment) | Fear related to unexpected treatments that generate costs. Availability of financial resources. |
| **Oshima (2021)** | **Financial Impact Because of Indirect Costs** Regardless of treatment type or time since diagnosis, a common theme for survey participants was the unexpected indirect and hidden costs associated with breast cancer treatment, represented in 136 (26.6%) of responses. Participants reported that their breast cancer diagnosis resulted in career repercussions beyond lost vacation and sick time, for example, long-term professional opportunities or slowed or halted workplace promotions. In addition, hidden costs were broadly characterized. For some, the additional expense came from the frequent travel that was required to receive care from in-network providers. For others, the ancillary cost of wigs, new clothing, and other cosmetic products proved to be unexpectedly costly. One of the most significant and frequently reported hidden expenses was the time they needed to take off work to undergo treatment, leading to substantial lost wages and depleted vacation or sick time. | *“Living 100 miles from Dr’s and treatments—traveling was my biggest hardship”* | Travel distance to be bridged for treatment is a cost to be met | Financial barriers |
| **Oshima (2021)** |  | *“My husband is delaying his retirement in part because of the uncertainty of insurance due to the repeal of the ACA and whether or not my pre-existing conditions would be covered (I’m not old enough for Medicare yet) and the costs of buying private insurance”* | My husband has to work more because of insufficient insurance | Support from close family members. Role of insurance. |
| **Oshima (2021)** | **Cost Transparency and Communication** Many women (n 5 42, 8.2%) reported a lack of cost transparency and communication; for some, cost discussions did not arise, yet in others, cost conversations were actively averted by patients or providers. Participants reported avoiding cost discussions because of the perceived association that cost of treatment correlated with the quality of care; thus, expressing an interest in reducing costsmight negatively affect their treatment. One participant also reported that her oncologist verbalized that she would not discuss costs, for fear it would cloud her clinical judgment. Despite this, most survey participants reported a desire for cost transparency at the time of diagnosis and when facing treatment decisions along the continuum of care. | *“Costs were not discussed prior to surgery. It probably would not have changed my decision if costs had been discussed”* | I was not informed about the costs before having the surgery. Discussing the costs beforehand would not have changed my choice of treatment. | Informed decisions on treatment options |
|  |  | *“Our savings had been used to pay for medical care for a developmentally disabled daughter. The only asset we had was our house. I brought up the cost with every doctor I met and every time I had an appointment. My oncologist finally told me not to mention it again as she did not want my financial situation to affect her medical decisions!”* | Not having savings makes FT worse. I needed cost information, but this could affect my medical decisions. | Not having savings makes FT worse. Need for information on treatment costs. Cost-based treatment choice. |
| **Oshima (2021)** | **Navigating Insurance** Interacting with insurance was a key element of many participants’ self-reported breast cancer experience (n 5 344, 67.3%). When compared with national averages, study participants were of higher socioeconomic status and education level, with many having pre-existing comprehensive insurance coverage. Several participants reported gratitude for their extensive insurance coverage that allowed them to receive breast cancer treatment with manageable out-of-pocket expenses. Additionally, several participants expressed frustration regarding the time and energy it took to negotiate insurance coverage of their treatment while managing the physical and mental debilitation that accompanies complex oncology treatment. Other women commented on the experience of changing insurance plans over the course ofmultiple different cancer diagnoses and the financial implications to their lives. These women experienced firsthand the financial devastation that can result from being underinsured, a realitymade clearer after undergoing different rounds of treatment both with and without comprehensive coverage. Regardless of prior coverage, for many participants, the uncertainty of the changing national insurance landscape contributed to underlying stress and fear over potential coverage loss. | *“I could not have done this without insurance. I had two surgeries after the initial lumpectomy raising the total cost even higher. My left breast is now more expensive than my home”* | The treatments I had to do are very expensive, I could not have dealt with them without insurance | Role of insurance in good coverage of high treatment costs |
|  |  | *“It isn’t so much the COST of treatment, but the FIGHT with my private insurance company over things they would decline. It is very difficult to muster the energy to fight the insurance company when one is exhausted because of effects of chemo, surgery and radiation”* | It is difficult to fight against my private insurance to have the costs covered because of the lack of energy given by the side effects of treatment. | Difficulty of being in active treatment and being debilitated and having to fight insurance companies to cover costs. |
| **Chebli (2020)** | **Individual-level Determinants: Lack of knowledge and financial planning** Both survivors and healthcare professionals identified the lack of knowledge and delayed financial planning as individual-level determinants of financial toxicity for all breast cancer survivors. These individual-level factors were considered universally important, but more common among disadvantaged communities. Healthcare professionals perceived that Latina breast cancer patients’ lack of knowledge compromised their ability to obtain financial assistance efficiently. Latina breast cancer survivors explained these delays differently. They emphasized that survival at any cost was initially prioritized over financial concerns, in line with theoretical models regarding disadvantaged communities’ competing priorities. | ***Latinas’ limited knowledge about the financial aspects of breast cancer*** *“I have a private insurance from my husband…we don’t worry about anything, right? But there was a time in which my husband was receiving notices, that he became aware of the actual costs [related to my breast cancer] because they were very expensive.” (Survivor Focus Group #1 Respondent #6)* | At first we did not worry because my husband had private insurance, but then we realized the actual costs were very high. | Not knowing the actual costs of treatments worsens FT |
|  |  | *Latinas’ prioritization of survival over financial aspects “Regarding the money, I didn’t even think about it. One would just say “operate me, so that I can survive from this.” (Survivor Focus Group #1 Respondent #3)“At that moment when they told me you have cancer, I did not think about how much they would charge me… at that moment it’s not when you think about money. You do not think about how much they will charge me, [it’s whether] I’ll be fine.” (Survivor Focus Group #1 Respondent #5)* | The priority was not money but surgery to survive. At the time of diagnosis, the priority was not money but health. | Health and survival are the priorities respect financial concerns. |
| **Chebli (2020)** | **Interpersonal-level Determinants: Access to Social Networks with Cancer Experiences** Healthcare professionals and survivors both emphasized the importance of access to individuals with relevant cancer experiences within their networks (e.g., other survivors, caregivers of cancer patients) as a protective interpersonal-level determinant of financial toxicity. Healthcare professionals perceived that survivors with such access had greater knowledge about financial assistance programs and applied for financial assistance early in the process of cancer treatment. Survivors confirmed the importance of social networks for sharing information. They specifically described situations wherein they themselves disseminated information on financial assistance to family members. | ***Social networks that have lived experiences with cancer can provide Latinas with information about financial resources*** *“Now I’m learning so much more with the [support] group because at least I know where to ask [for financial assistance] at least, right?” (Focus Group #1 Respondent 4)* | Now I am learning a lot more because the group supports me, I know who to ask about financial assistance. | Information support on financial assistance from community associations/groups |
|  |  | *“I’m going to tell my cousin, because in March they removed both her breasts and she paid a lot for her medicine. So, I am going to tell her [about financial assistance].” (Focus Group #2 Respondent #6)* | I will share informaizons with my cousin about financial assistance. | Information support on financial assistance from family members |
|  |  | *“I have sent people who also do not have insurance to [social worker name].” (Focus Group #2, Respondent 7)* | I have sent people without insurance from those who share information | Information support on financial assistance from patients |
| **Chebli (2020)** | **Community-level Determinants: Cultural Norms and Community Dynamics** Healthcare professionals highlighted cultural norms (e.g., vergüenza/embarrassment, stigma) and community dynamics regarding documentation status as community-level determinants of financial toxicity. Healthcare professionals and Latina breast cancer survivors identified the importance of culturally- and linguistically-congruent personnel and organizations to address cultural norms, language barriers, and barriers associated with non-citizenship status. Survivors specifically expressed gratitude for a local community-based organization led by Latina breast cancer survivors. | ***Culturally astute Latino community-based organizations facilitate Latinas’ willingness to access resources*** *“I am going to call and ask what is covered because it is important to know and there’s people that speak Spanish. If you don’t get something let them know and tell them to explain it again.” (Focus Group #2 Respondent #6)* | It is important to ask, understand (if you speak a different language) and know what is covered and what is not. | Information support on financial assistance from community associations/groups |
|  |  | *“Thanks to the girls from [a Latino community-based organization] who are our angels. They have helped us a lot, they are always helping us with appointments—they simply help us fill out papers to see if we qualify or do not qualify. But thanks to them. They are the ones who are helping us a lot, we thank them.” (Focus Group #1 Respondent #3)* | We received practical support from community associations. We are grateful to them. | Information support on financial assistance from community associations/groups |
|  | Organizational/Healthcare Policy-level Determinants: Limitations of Existing Financial Assistance Programs Healthcare professionals and survivors referenced several financial assistance programs commonly utilized by Latina breast cancer patients and survivors from disadvantaged communities. These included public (e.g. local affiliate of the National Breast and Cervical Cancer Early Detection Program), foundational (e.g. Patient Access Network, Patient Advocate Foundation) and hospital-based programs. These programs however had some limitations. First, some programs had restrictive eligibility criteria in terms of insurance status and income. Ineligible patients who were underinsured and/or who had minimal assets reported high levels of financial stress. Relatedly, these program criteria impacted eligible participants’ behaviors in potentially maladaptive ways. Having to re-apply for programs without guarantee of support was a major stressor. As well, some participants declined employer-based health insurance plans, because these plans provided less comprehensive coverage than financial assistance programs. In addition, healthcare professionals discussed most financial assistance programs’ nearly exclusive focus on medical costs during treatment. Both groups agreed that survivors lacked access to financial resources for competing costs (e.g., costs of living, family care) and indirect costs associated with treatment (e.g., travel, housing). Finally, healthcare professionals stressed the limited amounts of money offered and the instability of funds. These problems were particularly common for financial assistance programs that covered all costs and were discretionary. | ***Latinas who are not eligible for programs suffer worse financial burden than eligible counterparts*** *“Because we are a low income family the coverage is up to the sky. When you are low income the coverage is 100% but when you aren’t low income then you have to pay.” (Focus Group #2 Respondent #2)* | Having a low income results in maximum cost coverage. | Eligibility criteria for financial assistance programs. |
|  |  | *“I was diagnosed this past October…and there’s a lot of things we have to worry about including economically plus or well-being…when you have health insurance, costs are higher because you don’t qualify as low income or having insurance or etc. and well sometimes you have too many bills.” (Focus Group #2 Respondent #9)* | Diagnosis leads to economic concerns. Having insurance increases costs because it is not a requirement of people with low incomes. | Disease stage influences FT. Eligibility criteria for financial assistance programs. |
|  |  | *Limited availability of financial assistance programs post-treatment negatively impacts Latinas “We were left with nothing…then you are in recovery. Every time I went to the doctor after the operation, the nurse told me, ‘You have this [bill].’ I would say, ‘Look, please right now I am* | We had nothing, I was in recovery, and every vote I went to the doctor the nurse would present me with the bill and I would reply that I was not working at the moment. | Not having financial resources and not working worsen FT |
|  |  |  | I was financially stressed because of the restrictive eligibility criteria for applying for insurance coverage | Financial stress due to eligibility for health insurance coverage |
|  |  |  | Reapplying for health coverage was a source of stress | Financial stress due to the process of applying for health coverage |
|  |  |  | I refused the insurance coverage offered by the employer because it was not as comprehensive as that of the financial assistance programs | Insurance: limited health coverage |
| **Iddrisu (2020)** | **Socioeconomic concerns** This theme has two subthemes which illuminate the employment difficulties and financial challenges participants encountered. Most of the participants reported that they stopped work to concentrate on their health. One participant said she took a year leave from work without pay. Almost all participants complained of huge hospital bills, and also, most of the treatments were not covered by the national health insurance scheme. Due to the financial challenges, one participant could not start the chemotherapy prescribed for her after surgery. Another participant struggled financially to complete subsequent sections of the chemotherapy. |  |  |  |
| **Iddrisu (2020)** | **Work and employment concerns** Most of the participants abandoned their work to take care of themselves. Some participants cited treatment effects and stress as reasons for their inability to work. | *I get stressed up and stress is not good so I had to slow down for a while. I finally withdrew from work totally. (W1)* | I withdrew from work because of stress | Withdrawal from work due to stress |
|  |  | *My condition has affected my work a lot, my work is a practically oriented one and I have to go up and down with my students to see whether what I asked them to do they are doing it. But now I cannot move around. I feel dissatisfied and uncomfortable with my work output. (W11)* | My illness has negatively affected my work performance | Illness affecting work performance |
| **Iddrisu (2020)** | Some participants took leave from work without salary to recover before going back to work. | *…I have asked for leave without pay for a year to recover fully. (W4)* | I asked for an unpaid leave of absence to fully recover | Unpaid leave of absence from work |
|  |  | *…I have stopped work until I am done with chemo and my surgery. I do not have the strength to go to the market. (W9)* | I stopped work during treatment because I didn't have the strength | Job changes affecting earnings |
| **Iddrisu (2020)** | **Cost of treatment and financial challenges** Most of the participants complained of financial constraints and the high cost of bills with the treatment of breast cancer. The inability of the national health insurance to cover the full cost of treatment made it difficult for some participants to initiate treatment on time. | *… because of financial challenges, I could not go for chemotherapy. I also realized that it is not just once but involves six cycles so there was no way I could afford it. More so, I was told insurance does not cover the drugs and they are expensive too. (W12)* | I couldn't pay for chemotherapy | Lack of money to pay for treatment |
|  |  | *I was told to pay three thousand cedis (about 500 dollars) for the surgery alone and I did not have money so I left…The laboratory investigations alone cost about six hundred ($) not to talk about the medications. (W2)* | I could not pay for the surgery | Lack of money to pay for treatment |
| **Iddrisu (2020)** | A participant lamented that she could not finance her surgery because she had spent all her monies taking care of herself. She is unable to complain when in distress because the doctors would not care about her financial status but would prescribe as she complains. | *I was told if I do not go for the surgery, cancer can kill me but I do not have money to go for the surgery… I have spent all my money on this disease. Now getting the subsequent cycles of the chemo is also difficult. As for the doctors, they do not care whether you have the money or not. Once you complain they will prescribe so if something is worrying me, I am not able to complain again. (W8)* | Doctors did not care about my financial situation | Lack of interest of financial condition by physicians |
| **Kong (2020)** | **Health Costs** Health costs are discussed in Table 2. Among participants from the Ministry of Health hospitals, the majority described conventional treatment costs as affordable and did not incur substantial financial burden in paying for their treatments. “After we get our bill from the government hospital, we are very, very thankful. It is still affordable,” said a 56-year-old Chinese patient from the middle-income group who had no medical insurance coverage.   Although participants who were insured indicated that their financial burden in paying for conventional cancer therapy was alleviated by theirmedical insurance, **many expressed that they had poor prior knowledge of their insurance coverage, such as the insured amount, benefit packages, and coverage limitations and exclusions**. **Such patients were “shocked” and distressed upon learning that their medical insurance policies were unable to fully cover their cancer treatment costs**. **Notably, underinsurance led to financial hardship in some participants who still needed to pay out of pocket for certain aspects of their treatment that were not included in their benefit package**. These women reported forgoing certain recommended treatments as they were unable to afford them on their own. Some participants described experiencing financial burden only toward the end of their treatment when their insurance has been fully used up.   Patients also mentioned that this led some of them who initially sought treatment in private hospitals to be referred to public facilities upon exhausting their insurance policy limits. Participants also talked extensively on financial difficulties stemming from their insurance’s “reimbursement basis” policy, as not only do they have to pay first and then proceed with the reimbursement paperwork, but they also had to wait for a long period for the claims to be reimbursed. **These patients also reported being distressed because of the fact that they had to have “cash in hand ready,” which could be a huge sum at times, to pay for their ongoing treatments while waiting for their previous claims to be reimbursed**. It was also emphasized that there remained an unmet need for a more efficient system to facilitate obtainment of the necessary supporting documents for their insurance claims. The need to spend on essential items, such as breast prosthesis, mastectomy bras, corsets, and wigs, was repeatedly highlighted by a number of participants.  **Although women from higher socioeconomic status generally found these items to be affordable, those from lower socioeconomic status found them to be overpriced.** Because of the high costs, some participants decided to forgo buying these items. A number of women also highlighted that they were “forced” to pay out of-pocket for their breast reconstruction surgery, which was categorized by their existing insurance policies as a cosmetic surgery and thus not reimbursable. Apart from spending on conventional medical care, **participants also mentioned spending on traditional and complementary medicine**, which was deemed as costly. However, most women felt compelled to spend on traditional and complementary medicine because of the need “to try.” As mentioned by one participant, even though she “didn’t really understand whether it [traditional and complementary medicine] was good or not.” she felt like she was “not doing her best” if she “did not give it a try. | ***Poor insurance literacy*** *We just know that cancer is covered. 57 years old, Chinese, low-income, personal medical insurance coverage* |  |  |
|  |  | *I took life insurance and they said it covers 36 critical illnesses which include cancer. Only later when I was diagnosed then I know. They said it [cancer] should have spread all over the body, only then I can claim. 47 years old, Indian, low-income, no medical insurance coverage* | The terms of the insurance coverage had not been explained to me in detail | Failure to explain insurance coverage clauses |
|  |  |  | I was not aware of what my insurance would cover. | Failure to explain insurance coverage clauses |
|  |  | ***Underinsurance (high out-of-pocket,limit exhaustion)*** *They advised me to take hormone therapy for one year which costs about MYR150K (USD36K) [whole course]. The moment I heard the cost I said I don’t want to undergo hormone therapy. My insurance will not cover the cost due the limit of my coverage plan…. I am struggling financially and I do not think I can afford it… I feel that financial crisis is worse than my health issues. 47 years old, Indian, low-income, employment medical insurance coverage* | I didn't have hormone therapy because my insurance wouldn't pay for the treatment and I couldn't afford it. | Waiving treatment due to lack of insurance coverage of the cost |
|  |  |  | Economic problems were more serious than my health problems | Economic problems were more serious than my health problems |
|  |  |  | I had financial problems because I had to incur medical expenses that were not covered, and this caused me distress | Financial stress caused by medical expenses |
|  |  | *For adjuvant therapies I had to pay using my own money because there was no money left in my company insurance [after using it for surgery]. So basically, I suffered financially after my surgery. 39 years old, Malay, low-income, employment medical insurance coverage* | I had financial difficulties only toward the end of the treatment when the insurance had run out | Major economic difficulties as insurance runs out |
|  |  | ***Reimbursement policies*** *That is a burden because you have to prepare the cash… so you have to always come out with the cash and claim later. And the claim may not come instantly. So you have to have cash in hand. That is the difficulty. 41 years old, Chinese, middle-income, personal medical insurance coverage* | In order to get treatment I still had to have money available, and this caused me distress | Financial stress caused by medical expenses |
|  |  | *As a patient, it is a big hassle for us to go through the process of getting our claim form… they must make sure that the claim process is easy. But that’s not the case, the process is very troublesome. They should have a systematic way to ease the process of preparing documents for claims. 47 years old, Indian, low-income, employment medical insurance coverage* | The procedure for claiming remittance of expenses was not simple, and the reimbursement did not come to me immediately | Claim: complex and not immediate |
|  |  | ***Coverage***  *It [mastectomy bra and prosthesis] comes to MYR1000+ (USD 241) which I am quite mindful. If the prosthesis is cheaper, then I will pay for it. But since it is over MYR1000, I am willing to forego it because of costs. 56 years old, Chinese, middle-income, no medical insurance coverage* | I gave up breast implants because of the cost | Giving up breast implants because of the cost |
|  |  | *I had a reconstruction surgery and that has to be paid on our own because it is cosmetic surgery. It costs about MYR 15,900 (USD3830). 44 years old, Malay, high-income, personal and employment medical insurance coverage* | Breast implant would have been my charge because it is considered cosmetic surgery | Giving up breast implants because of the cost |
|  |  | ***Traditional and complementary medicine*** *I didn’t really understand whether it was good or not… just psychologically… you know… if I don’t go, feels like not good like that. But because it is too expensive so I decided not to go anymore. But whether it is good or bad, I really don’t know… Because if you don’t do it, it’s like I amnot doingmy best. 65 years old, Chinese, low-income, nomedical insurance coverage* | Although I didn't know if it was of benefit, I paid for complementary medicine because if I didn't it was as if I was doing my best | Incur non-medical expenses (complementary medicine) so as not to feel guilty |
|  |  |  | Socioeconomic level affects perception of financial toxicity | Socioeconomic status affects FT |
| **Kong (2020)** | **Nonhealth Costs** The financial needs for nonhealth costs were found to be different depending on the participant’s socioeconomic status (Table 3).   Participants from lower socioeconomic status described facing difficulties affording very basic necessities, such as food, following their cancer diagnosis. Meanwhile, participants from higher income households brought up the need to spend on perceived “better” food, such as organic food, as well as dietary supplements.  This substantially increased their household expenses, as they were not just spending for themselves but also for their families. Some patients also mentioned spending on appliances such as fruit juicers or special water filters. Transportation costs to the hospital for treatment and follow-up visits were also frequently reported as a burden by participants from lower socioeconomic status. As parking fees were mainly charged at an hourly rate, it became expensive, as participants from public hospitals tended to spend at least half a day in the hospital for their appointments. The cost for transportation further escalates for patients requiring commuting to hospital, for instance for their radiotherapy sessions. This was especially burdensome during the first year after their cancer diagnosis in which hospital follow-ups are usually as often as every other week. Participants from higher socioeconomic status also cited financial burden due to expenditures on childcare or household help following their cancer diagnosis. Despite the high cost, they viewed that these were necessary, as they were unable to care for their kids or perform household chores especially when they were undergoing active cancer treatment. | ***Daily living*** *I didn’t have enough money to run the family and had to return back to work earlier… There was a time when I gave my kids to eat fried rice and fried egg every single day… we didn’t have enough money so I had to make sure the small amount of money we had will last the entire month. 35 years old, Malay, low-income, employment medical insurance coverage* | I had no money to support the family, and because of that I had to go back to work earlier | Having made work decisions according to financial burdens |
|  |  |  | I had to make sure that the little money I had was enough for the whole month | Economic resource planning to survive in the short term |
|  |  |  | I had to change the family diet due to economic problems | Reduction in spending on essential goods |
|  |  | *For those who believe in their diet, they will go for more clean food, which is organic… food [expenses] definitely raise up like crazy, double, triple… Thinking for the sake of the other family members because you want them to eat well as well. 41 years old, Chinese, middle-income, personal medical insurance coverage* | I had to bear the cost of parking and diesel fuel to have radiation therapy every day | Incur non-medical expenses (gasoline, parking) in order to get treatment |
|  |  | ***Transportation*** *It is a burden. Because you must come for radiotherapy every day. Everyday parking is MYR3 (USD0.75). I came 23 times… That is not including the costs for petrol. 47 years old, Indian, low-income, no medical insurance coverage* | The cost of parking was high and sometimes I had to stay in the hospital half a day or even all day | Incur non-medical expenses (parking) in order to get treatment |
|  |  | *Parking here is not cheap at all. And when you wait, its not just one or two hours. Sometimes half a day, sometimes even the whole day. 41 years old, Chinese, low-income, no medical insurance coverage* | The cost of transportation and parking were particularly high during the first post-diagnosis year because hospital appointments were more frequent | Incur non-medical expenses (parking) in order to get treatment |
|  |  | ***Household help/childcare*** *I had to hire 2 maids to come once a week… I also had to hire someone to cook for me… I needed to rest. 44 years old, Malay, high-income, personal and employment medical insurance coverage* | I needed to rest so I hired people to come and clean and feed me at home | Incurring non-medical expenses |
|  |  |  | Socioeconomic level affects perception of financial toxicity | Socioeconomic status affects FT |
|  |  |  | I could afford to spend more to buy better food and tools to eat and drink better | Socioeconomic status on the FT |
| **Kong (2020)** | **Employment and Earnings** Participants who were employed prior to cancer diagnosisreported financial burden upon diagnosis or after starting their cancer treatment. Many experienced reductions in income due to the prolonged unpaid work absences or reduced productivity.  The loss of income not only strained household budgets but also exacerbated the burden of paying for health care and treatment costs (Table 4). Many study participants were unable to continue working following diagnoses with breast cancer. Although some employed participants voluntarily resigned, some reported being terminated as they were often absent from work while being on active treatment, or because of the side effects of cancer therapy. Although some participants reported that they received considerable support from their employer and were offered workplace flexibility such as reduction in workload or working hours, some participants reported that their employer and/or colleagues failed to show empathy and continued giving them difficult tasks.  A few participants also described facing discrimination at workplace such as being overlooked for a promotion or in getting hired for a new job because of their cancer. Cancer diagnosis and treatment also adversely impacted participants who were self-employed who reported decreased work productivity due to ill health. “Although I amself-employed. For me, I go outstation a lot but when we get sick like this, what we target cannot reach, things like sales…,” said a 43-year old participant from a high income household who had personal medical insurance coverage. In the discussion, participants also noted that their cancer diagnoses negatively impacted the employment and earnings of their spouses or caregivers, further diminishing their household incomes | ***Income loss*** *It was quite difficult [to pay for treatment costs] because the salary is not in. My budget was just enough for the household… It was very difficult for me at that time… When we have limited income, the budget is very tight. 35 years old, Malay, low-income, employment medical insurance coverage* | The money I had on hand was for the family and it was quite difficult to pay for the treatment | Supporting medical expenses and supporting the family |
|  |  | *I am self-employed. For me, I go outstation a lot but when we get sick like this, what we target cannot reach, things like sales. 43 years old, Malay, high-income, personal medical insurance coverage* | I couldn't meet my work (sales) goals and earn as much as before because I was on sick leave | Job changes affecting earnings |
|  |  | ***Job loss*** *When I went back to work, the boss said he didn’t need me anymore, that I don’t need to come back… they fired me. 37 years old, Chinese, middle-income, personal and employment medical insurance coverage* | I was fired because the employer no longer needed me | Change in working condition affects FT |
|  |  | ***Workplace flexibility*** *I work in a factory so my job scope requires me to do heavy jobs. Even though the doctor advised me not to lift heavy things, I had to do it because it is my job. Even if I try to explain to my boss about my condition, they just disregard my complaints. 35 years old, Malay, low-income, employment medical insurance coverage* | I had to do work activities that I should not have done because of my employer's health reasons | Constructs without relevant content to answer research questions |
|  |  | ***Discrimination*** *I applied for a new job. But upon doing a background check they did find out that I have cancer. Although I satisfied all the requirements but my application was rejected because of cancer… This is like discrimination towards us. 39 years old, Malay, low-income, employment medical insurance coverage* | I received discrimination while searching for a new job because of my cancer diagnosis | Limited job future due to cancer |
|  |  | ***Impact on carers*** *My husband is a taxi driver. So when I have treatment, he can’t work as he needs to take care of our 3 year old child… my cancer affected his income. 44 years old, Indian, low-income, personal medical insurance coverage* | The tumor affected my husband's income because while I was having treatment he had to work less to take care of our children | Job changes of family members affecting earnings |
| **Kong (2020)** | **Financial Assistance** To cope with treatment costs and decreased household income, many participants described tapping into their personal savings, with some even exhausting their savings (Table 5). Participants also mentioned borrowing money from informal sources such as their relatives, friends, or even employers. As described by one participant, “somehow I have to look for money, if I don’t, then I will die,” illustrating the dire need for money in this self perceived life or death situation. Many participants highlighted their lack of eligibility to access formal financial resources such as from the Social Security Organization (SOCSO) or Employees Provident Fund. This was due to either their disease status or financial status, both of which were deemed not severe enough to receive financial aid. This rendered needy participants who were deemed “not ill enough” or whose financial statuses lay just above the cutoff for financial aid, unable to access formal financial support.  The participants also highlighted the lack of transparency and efficiency in the application system for financial assistance programs. In the discussions, they frequently brought up their frustration with the lengthy, complicated, and tedious application processes. Despite taking the trouble to go through the “red tape,” their applications were often rejected without reasonable justifications. The need for a financial navigator was also brought up by some participants. It was suggested that having a specific person to guide them toward the appropriate financial assistance programs, as well as in assisting them in the related paperwork, would be most helpful. Furthermore, the presence of a financial navigator to help with insurance claims was also deemed as a pressing need. | ***Informal financial resources*** *I am facing financial difficulties, I have to withdraw my savings to support my daily living. 39 years old, Malay, low-income, employment medical insurance coverage* | I am using my savings to manage daily expenses | Depletion of savings, assets, pension funds |
|  |  | *I have to ask for help from my relatives. Somehow I have to look for the money, if I don’t then I will die. 59 years old, Indian, low-income, no medical insurance coverage* | I asked my family members for financial support | Financial support from family members |
|  |  | ***Formal financial resources (****Eligibility) They just said I do not have a valid reason to withdraw from my Employee Provident Fund (EPF) because I was only at Stage 2. Then I applied for Social Security Organisation (SOCSO) and the same thing happened. I was rejected because I was not sick enough and my case is not that serious. 39 years old, Malay, middle-income, employment medical insurance coverage* | I did not have the criteria to fall under formal financial support systems because my cancer or financial situation was not severe enough. | Lack of support for formal support systems |
|  |  | ***Formal financial resources*** *(Transparency and efficiency) My doctor say I am already stage 4, so I apply [for SOCSO] but they rejected me. They asked me to get the latest report to appeal so I went here and there to get medical report. but the report department ask you to wait one month for the new report. After that, I submitted to SOCSO and also have to wait to go for the interview. 51 years old, Chinese, low-income, personal medical insurance coverage* | The process to re-enter formal financial support systems has been complicated | Receiving support from formal support systems has been complicated |
|  |  |  | Even though I had gone through all the paperwork my application was rejected without a reason | Lack of support from formal support systems |
|  |  | ***Formal financial resources*** *(Financial navigation) I wish there is someone to be in charge of the whole process. That will be easier. For example, a social worker or someone who is hired to specifically manage the claims. If there is a specific person, they can easily do the job. 51 years old, Malay, high-income, employmentmedical insurance coverage.  A financial advisor. They [hospitals] should have a consultant, who can gather the patients and advise them on how to get money from SOCSO, or the government, or any charity organisation… to be able to advise the newly diagnosed patients on how to seek help financially… maybe include a consultancy to advise you on career issues too. 41 years old, Chinese, high-income, personal and employment medical insurance coverage* | I needed to have a contact person to talk about the financial aspects and to handle the paperwork and reimbursement paperwork | Need to have a case manager |
| **Dean (2019) SCC** | **Theme 1: Economic burden is cumulative and cascades over time; managing an adverse treatment effect presents ongoing challenges.**  The use of savings to cover medical was common in all respondent interviews. For some women, covering medical costs compromised their ability to manage basic needs like utility bills. Women with lymphedema were more likely to relay that the upfront costs associated with cancer set off a cascade of financial challenges that continues to affect their current economic situation.   Participants described current effects such as decreased ability to help family, support their children’s educational endeavors, and retire. Ongoing costs for lymphedema care needs exacerbated economic burden and compromised participants’ ability to obtain care for their current lymphedema needs. | ***Use of assets, loans and lasting impact of cost accrual*** ***Respondents with lymphedema*** *I had to take my 401 K money and like pay bills, buy medicine because I did not have any medical coverage… all the moneys that I had saved up that would have sustained me [as a retiree] was gone… in terms of the money that I would have wanted to contribute [to retirement and children’s college funds], you know, I wasn’t able to and when I was able to-- I’m 61 so I’ll never get caught up with that so, yeah. – age 60; private insurance It still affects our economic situation … we still feel the effects of the economic problems …. We had the co-pays. We had [lymphedema] therapies, different therapies... Massage. And, of course, you know, the sleeves…it seems like we can never, ever catch up to have a little bit extra. – age 56; private insurance* ***Respondents with no lymphedema*** *So I went for a long time just basically on my savings and family helping me. – age 52; private insurance So and I was able to get a small loan and pay off some bills. So, you know, that helped. – age 69; public & private insurance* | I don't have medical coverage, I spent my savings that I would have needed for retirement and my children's education. (I have lymphedema). I have medical coverage and I relied on savings and help from family, today I also made a small financing (I don't have lymphedema) | I don't have medical coverage, I spent my savings that I would have needed for retirement and my children's education. (I have lymphedema). I have medical coverage and I relied on savings and help from family, today I also made a small financing (I don't have lymphedema) |
|  |  |  |  | Inability to cover expenses for basic goods |
|  |  | ***Balancing health costs with utility bill costs*** *Respondents with lymphedema It was just like, just a lot of financial burden so it was stressful where I would have liked to have had the experience while I was convalescing to be like not worried about are my lights gonna get shut off? And sometimes that happened and it was just rough. – age 60; private insurance* ***Respondents with no lymphedema*** *… So I was no longer able to work, ‘cause I had three surgeries … And then I had to do chemo and then I did radiation … I had my lights cut off. I had my water shut off. I had my gas shut off. And I would have to go up to the hospital and get slips to get them cut back on…And they would cut my lights off for, like, maybe $100... – age 69; public & private insurance* | I had problems to pay the electricity bills, I would have liked to have only the disease problem not also the financial problem. (Both with lymphedema and without) | Difficulty paying bills |
|  |  | ***Increased costs due to lymphedema-- specific health needs*** *So having to go to physical therapy, it’s $30 each time I go…So I have had toactually ceased going because I just do not have the money. – age 62; private insurance I just ordered my replacement sleeve on Monday, and I had to give my credit card for $420 before they would put in that order … This was one sleeve and glove – age 66; public insurance* | I had out-of-pocket costs for orthotics and specific treatments for lymphedema (I have lymphedema) | Incur medical expenses not covered by insurance |
| **Dean (2019) SCC** | **Theme 2: Lymphedema care needs are unlikely to be covered by insurance, which contributes to higher longterm costs and compromises a patient’s ability to manage lymphedema symptoms.**  Respondents in both groups reported out-of-pocket health care costs and shifting costs to other parties (including family, employers, social service organizations, and advocacy groups). Participants described the need to use leftovers of patients’ medications to cope with their economic burden. Women who did not have lymphedema were more likely to report out-of-pocket costs accrued closer to the period of their cancer treatment for supplemental insurance, co-pays, and treatment, while women with lymphedema reported additional ongoing long-term out-of-pockets costs for lymphedema care in the form of ongoing physical therapy, lymphedema specialists, sleeves, and garments that were not covered by insurance. Even participants with private insurance did not always receive necessary lymphedema-specific care because of the cost burden. Changes in insurance, especially when changes in status led to less lymphedema coverage, further stymied their ability to manage ongoing lymphedema needs. | ***Insufficiency of Medicaid to cover lymphedema needs Respondents with lymphedema*** *The physical therapy is covered with my Medicare and the secondary insurance, but if I were to get any garments, or new bandages, and everything, I am gonna have to do the out of pocket stuff, and I know that ran into, like, $95 for the bandages, and then the tape that you buy to wrap the bandages, the Ace, that runs to, like, $5 a roll. – age 73; public & private insurance  I cannot basically afford to buy the compression sleeve... And insurance does not cover it… I had [private insurance that] did give me one sleeve. Right after that, they changed my health insurance [to Medicare], so it went from getting the sleeve to not getting the sleeve. – age 68; public insurance* ***Respondents with no lymphedema*** *I pay for supplemental insurance to cover it, and I am dealing with... Medicare telling me what I can and cannot take …My supplemental insurance, to help cover the doctors and stuff, is $227 a month, and then your supplemental to cover your drugs is another $45 a month. And of course, Medicare’s not free. I know everybody acts like it is, but it’s not. Last time I looked, it was $166 bucks every three months. – age 73; public insurance* | I can't cover all the medical aid I would need for lymphedema, my insurance also changed the rules about it excluding expenses (I have lymphedema) | Incur medical expenses not covered by insurance |
|  |  | ***Out-of-pocket costs that are not covered by insurance Respondents with lymphedema*** *Right after my diagnosis and treatment and surgery, I had lymphedema and severe cording and banding... So I went to a [lymphedema] therapist, who at that time [the insurers] were not paying for that, it wasn’t reimbursed, so it was all out of pocket. – age 67; private insurance* ***Respondents with no lymphedema*** *The only thing that wasn’t covered was … a shot that was $100 and for-- I think for someone that’s not employed, that would be a difficult fee for them to have to pay – age 55; private insurance When you’re first diagnosed, you have to go to a bunch of specialists, and the specialists are $25 apiece. When you’re going three times a week … it does add up, even with insurance. – age 56; private insurance* | Even though I have private insurance the lymphedema expenses are out of pocket (I have lymphedema). I have insurance but it did not cover specialist expenses (I do not have lymphedema) | Incur medical expenses not covered by insurance |
| **Dean (2019) SCC** | **Theme 3: Productivity losses have long-term impact: breast cancer diagnosis may have influenced work opportunities and long-term earning potential, and breast cancer-related lymphedema may further decrease productivity losses at work.**  Both sets of participants spoke about long-term productivity losses. In some cases, women missed out on educational opportunities, modified work schedules, experienced job loss, pursued voluntary early retirement, or went back to work sooner than medically recommended. These experiences framed their subsequent health and lifestyles and still affect them currently. Women in both sets recalled needing additional help for duties around the house.Women with lymphedema were less likely to return to employment after cancer because of their additional physical challenges | ***Loss of career opportunities Respondents with lymphedema*** *I actually, I was teaching first grade at the time, which is very physically challenging, and I decided at the end of that school year, in June I retired – age 66; public insurance I lost my job ‘cause I got diagnosed with breast cancer so financially it was very difficult … I was out of work for almost a year … with the chemo… I was really sick and then I went back against the doctor’s orders ‘cause I needed to make money... When I came back to work that’s when they expected me to resume all of the duties... full force and…I got fired... – age 60; private insurance I used to do work with a lady with catering and stuff right and I could not use my arm because it was always in pain with the lymphedema…It was a setback…. I stopped [working]. – age 63; private insurance* ***Respondents with no lymphedema*** *When I went back, [the university] had taken away my financial aid, and consequently I was not able to complete my PhD. That’s an enormous hit. Consequently, although I am teaching at the University level … they will not hire me full time because I do not have the PhD. That would not have happened had I not had cancer... I also had chemo brain at that point... I mean, I still was getting good grades, but it was much harder work, but I also had no money, and we could not afford it, so I quit [the PhD program]. I have regretted that all these years. – age 59; private insurance* | I lost the job I was doing before because I could not sustain it and also the career opportunities (both with lymphedema and not) | Job changes affecting earnings |
|  |  | ***Needing help with daily activities Respondents with lymphedema*** *I just went around my normal household duties, and only thing I didn’t do-- I don’t think I did any ironing. – age 81; public insurance* ***Respondents with no lymphedema*** *During the first year, during treatment and immediately following, one, I was out of work for six months. Two, I needed help with childcare, transportation for children, housekeeping, meal prep. – age 60; private insurance* | I needed them to help me with housework and child management (both lymphedema and non-lymphedema) | Constructs without relevant content to answer research questions |
|  |  | ***Taking time off from work Respondents with lymphedema*** *Well, the surgery, I was-- I think I was out of work for maybe a month. For the lymphedema treatments, I just would go after work. I had to maybe leave early for work and leave early for radiation and that was about six weeks I think – age 63; private insurance I would schedule my chemo on a Friday, so it would give me Saturday and Sunday if I needed it. And, for my radiation, my employer would let me leave like at one o’clock every day... – age 60; public insurance* ***Respondents with no lymphedema*** *After I had my [breast cancer surgery] surgery, I wound up back in the hospital with a severe infection... because I did not get, or I did not understand, or I did not hear the proper way to keep it draining. And it backed up, and I wound up in the hospital for another four days with that. – age 73; I didn’t go back to work until part-time in November. So from June to November. And then, full-time, I guess, December or January … so we had the loss of salary plus additional outlay. – age 60; private insurance public insurance* | I had to be absent from work, my productivity dropped due to treatment and problems related to the disease (both lymphedema and non-lymphedema) | Job changes affecting earnings |
|  |  |  | I lost the job I was doing before because I could not sustain it and also the career opportunities (both with lymphedema and not) | Withdrawal from work |
| **Dean (2019) Cancer** | **Insurance** Women in our study with and without breast cancer-related lymphedema felt that it was challenging to navigate the insurance system and would have benefitted from a navigator to explain the process to them (Table 2). Frances’ (aged 56 years, no lymphedema) quotation illustrates how, although using insurance should be cost saving, it actually can cost the patient time and money and contributes to anxiety out of a sense of not knowing what would happen. The quotation highlights the gap between what patients need and what insurance companies are willing to cover without additional effort by the patient. Elizabeth (aged 55 years, no lymphedema) noted that having stable employment and income was critical to obtaining necessary cancer-related treatments not covered by insurance. Her comment reflects the reality that patients pay out-of-pocket for items that are not covered by insurance and thus need to maintain employment and a source of income to afford those noncovered items. Participants emphasized that having quality insurance that included coverage for cancer care and lymphedema treatment helped to minimize out-of-pocket costs, psychosocial costs (such as stress or anxiety), and time costs. Interviewees also emphasized the importance of affordable insurance coverage. Those with breast cancer-related lymphedema faced additional issues in getting insurance to pay for lymphedema self-management supplies. Garments, tape, and bandages must be replaced several times a year, which poses ongoing, lifetime costs that accumulate. Phyllis (age 74 years, has lymphedema) described forgoing lymphedema management because compression garments and bandaging were not covered by her insurance and cost up to $300 for garments and $100 for bandages and tape. Other patients mentioned that complementary and alternative medicine procedures, like acupuncture to reduce lymphedema-related swelling, generally were not covered by insurance, but should be. | ***Insurance navigation*** *I think insurance is always a challenge, because… I’ll give you one example: I went to my gynecologist, and he ordered for me to get an MRI, because I can’t do a mammogram, having implants. We got the MRI, and the insurance refused to pay for it. I went through all the appeals I could. I even talked to one of our state representatives, because I fought it as rigorously as I could, and I ended up paying over $2000 out of pocket. I sent them pages and pages of information, it was consuming so much of my time, oh, it was endless; it was the emotional feeling that an insurance company could do that to you. (Frances, age 56 y, −BCRL, EBS = 0)* | The paperwork for insurance reimbursement is very complicated, requiring the preparation of a huge amount of paperwork, and it happened to me that it did not go through. | Complexity of paperwork management for reimbursement |
|  |  |  | I am scared of the idea that it is not certain that the insurance company can agree to take over my medical expenses | Insurance: concern that it will not cover medical expenses |
|  |  | *Yeah. I wish there was some place that you could either go, or call, or whatever, that really understood how Medicare worked. (Jill, age 73 y, −BCRL, EBS = 7)* | I would like there to be a service available that I can ask about how to manage insurance-related issues | Information need about the economic implications of the disease and available options |
|  |  | *What could have helped me? Just more financial aid, really, because, like I said, when I first started out, I was with [one insurance], and they were taking care of it. I was getting the bras. I was getting the sleeve. Then, all of a sudden, oops [no more coverage for those items]. (Meredith, age 68 y, +BCRL).* | I would like there to be more financial support for indirect costs related to the disease | Need for more financial support to address indirect costs |
|  |  | *My COBRA had expired… I couldn’t afford to pay $1000 a month for insurance… Then, when I turned 65, I was qualified for Medicare. But Medicare…. But I didn’t have half of the coverage that I had [before]. So, the first thing I would say is to really check out what kind of insurance plans you have. Because I think it really does make a big difference. (Francis, age 66 y, +BCRL, EBS = 2)* | Future planning of one's insurance choices is critical, because choosing insurance with the appropriate coverages makes all the difference | Insurance: planning to have the appropriate coverages |
|  |  | ***Quality of insurance coverage*** *… They wanted me to have shots of Neupogen (filgrastim), and that wasn’t covered, but then apparently it was covered under major medical… But that could’ve been a problem, because I think at the time the shots were, like, $1500 apiece. (Eve, age 59 y, −BCRL, EBS = 0)* | The treatment choices I have made have been dictated by my affordability | Economic availability affects treatment choices |
|  |  | *My copays were not that expensive. Like I said, I had a pretty good insurance, so actually they paid for most everything. I believe at the time my copays were actually like $10. (Rachel, age 49 y, +BCRL, EBS = 0)* | Having good insurance has allowed me not to feel the economic burden of my treatment course | Insurance: having good health coverage affects FT |
|  |  | *…Exercise, that’s really important. I exercised a lot before I was diagnosed, so maybe that kind of sort of helped me and then I continue to—as much as I could, when I felt real good. (Elizabeth, age 55 y, −BCRL, EBS = 0)* | Exercise has helped me feel better | Constructs without relevant content to answer research questions |
|  |  | ***Affordable insurance coverage*** *I really do not think that issues that are direct outcomes of the type of cancer, people should have to have a co-pay for them to be treated, that you should have these exorbitant rates. (Susannah, age 62 y, −BCRL, EBS = 4)* | I don't think it's fair to charge a co-pay for expenses directly related to the effects of cancer | Injustice in the payment of health care co-payments |
|  |  | *This year, for my daughter (age 20 y), and myself, it is $820/mo for insurance with a $6000 deductible. This year, we got kicked out to the marketplace (ACA) and could have gone with a cheaper policy, but I was afraid, if I got sick, then what would happen? (Frances, age 56 y, BCRL, EBS = 0)* | Future planning of one's insurance choices is critical, because choosing insurance with the appropriate coverages makes all the difference | Planning for insurance coverage affects FT |
|  |  | *The only thing that wasn’t covered was… a shot that I had to take the next day [after chemotherapy treatment], and it was called a Neulasta (pegfilgrastim) shot, and that shot was a $100; and, for—I think for someone that’s not employed, that would be a difficult fee for them to have to pay, but since I worked, you know, it really wasn’t, like, a burden or anything. (Elizabeth, age 55 y, −BCRL, EBS = 0)* | Medical expenses not covered by insurance can be a very significant cost for people who do not have a job | Incurring medical expenses in the absence of a job |
|  |  | ***Insurance coverage for and access to lymphedema treatment*** *I can’t buy—can’t afford… I just can get the one. I say every 6 mo, yeah, you should at least be able to purchase another one. (Meredith, age 68 y, +BCRL)* | The treatment choices I have made have been dictated by my affordability | Therapeutic choices dictated by affordability |
|  |  | *My insurance didn’t cover the garment, and, you know, the sleeve, and the wrappings, …and that was, like, $300 and some …And then I had to buy the bandages, the tape to go with the bandages… $95 for the bandages, and then the tape that you buy to wrap the bandages, the Ace, that runs to, like, $5 dollars a roll for the tape, you know… Sad to say, I don’t follow through with it. I haven’t followed through. (Phyllis, age 73 y, +BCRL, EBS = 2)* | The treatment choices I have made have been dictated by my affordability | Therapeutic choices dictated by affordability |
|  |  | *The problem is with the Medicare and the secondary insurance… They only pay for X amount of [physical therapy] visits, so once you use them up, you can’t go back. You have to wait ‘til the next year. (Phyllis, age 73 y, +BCRL, EBS = 2)* | The health care expenses and services covered by the insurance are limited and, finished those, you are uncovered until the following year | Insurance: limited health coverage |
|  |  | *So I went to a [physical] therapist, who at that time …was all out of pocket. And it was significant. I went months and months… I was seeing her 3 times a week initially…during the first, I would say, 5, 6 y after my cancer diagnosis. It was a lot of out-of-pocket expense, which was not covered by insurance… It would have been helpful if insurance had paid some of that. (Rosemary, age 67 y, +BCRL, EBS = 0)* | There were major expenses for my health related to cancer treatments that I had to incur that were not covered by insurance. | Incur medical expenses not covered by insurance |
|  |  | *So I had used acupuncture in the past, so I did go to Doctor ___ for acupuncture…after 3 treatments, the fluid did go down in the hand… I believe in integrative medicine, and complementary medicine… Eastern and Western. (Rosemary, age 67 y, +BCRL, EBS = 0)* | Acupuncture helped me manage lymphedema | Constructs without relevant content to answer research questions |
| **Dean (2019) Cancer** | **Financial Assistance** Women also identified a lack of programs, or knowledge about programs, designed to financially assist women who were above the poverty line (Table 4). Interviewees felt that the process of finding financial support was challenging, because: 1) there were no referral services available, or 2) eligibility was limited, and 3) procuring assistance involved contacting multiple programs and incurring time costs. Several patients mentioned that grant programs for wigs and lymphedema garments available through hospitals or nonprofit organizations were helpful to them; however, others stated that their financial need was not deemed high enough to participate in these programs, yet they still could not afford their health needs. Outside of explicit financial assistance, women with lymphedema felt they would benefit from financial planning services. Oncology centers could create a list of resources to present at diagnosis. Interviewees suggested that screening and referral to financial services could occur during or near oncology visits and could be provided early on in the cancer treatment process at hospitals and insurance companies. Patients want to be made aware of financial services or resource navigators who could assist in explaining insurance coverage. These resources would benefit women so that, if they do find themselves facing a new challenge, then they already would have a point of reference to seek assistance. Specific recommendations related to financial assistance included the following. | ***Financial services for those above the poverty line*** *I went to apply for public assistance, and they said that I had “made too much money that year: come back next year.” And I said, “I might not be here next year.” And they’re like, “Okay, well, you know, you have to come back.” I couldn’t… I said, “I don’t want the money: can I get the medical?” And they were like, “No, come back in a year.” I just wanted medical, because that would help me with the, you know, the chemo drugs and stuff. (Nellie, age 60 y, +BCRL, EBS = 9)* | I was not granted insurance because, before I became ill, I had a well-paying job, and therefore I was not entitled to financial supports, or even coverage of medical expenses | Economic support not granted due to socioeconomic status prior to illness |
|  |  |  | When I asked for help from public assistance, I was asked to come back the following year, even though the following year might have been too late, because I might not have been there anymore | Financial support not timely |
|  |  | *I think there are things in place for people who are very financially needy, but not really for someone who is like… I don’t know what kind of classification I am, but who can still work, who doesn’t want to work, but if I would stop working, would be very financially needy. (Diane, age 70 y, −BCRL)* | Financial supports exist only for those who were in great financial difficulty even before the disease, but not for those who are having to manage work in the context of the disease | Socioeconomic status affects FT |
|  |  | ***Raising awareness of existing services*** *And when I found out what [services] was available, I utilized it. But, why do you have to go through that? Why isn’t there a service at the hospital that they can provide to cancer patients, these are the services that you can pick up the phone and call. (Mary, age 69 y, −BCRL, EBS = 11)* | Lack of a hospital information service that directs cancer patients to the support services available to them | Information need about the economic implications of the disease and available options |
|  |  | ***Financial counseling/planning*** *I would hope that, when you would get referred or get that first diagnosis; so, in the doctor’s offices, it would be great to have someone when you go see the doctor to say, “Listen, we want you to see our resource person to see if we can help you with any type of challenges that you may face. Maybe these aren’t your challenges but, in fact, if you have them, here.” In later years, I found out there were programs that would have helped you pay rent, help you do this, help you do that. (Nellie, age 60 y, +BCRL, EBS = 9)* | It would be nice if there was an informational/financial counselling service that welcomed you from the moment of diagnosis | Information need about the economic implications of the disease and available options |
|  |  | *Maybe trying to counsel women who are in a financial situation that are refusing treatment because they can’t afford it and, so, they just deny that they have anything wrong. (Emma, age 57 y, +BCRL, EBS = 3)* | There are women who cannot financially afford the treatment, so they refuse it and pretend to be well . It would be helpful to be able to offer them financial counseling | Information need about the economic implications of the disease and available options |
|  |  | *guess asking for help, asking for a financial planner or something like that; I guess being more aware, yeah, being more aware of what credit card debt does between the compound interest and things like that. (Ann, age 64 y, +BCRL, EBS = 5)* | It would be helpful to have a financial education service that explains to people what is involved in accumulating debt through credit card payments and the like | Information need about the economic implications of the disease and available options |
| **Japhet (2019)** | **Cost of treatment** This theme highlights the financial burden of breast cancer on the respondents. It is well known that breast cancer has a profound financial implication on its victims. The financial impact of breast cancer comes in two forms which are direct and indirect cost. |  |  |  |
| **Japhet (2019)** | **Direct cost** All the respondents revealed that they don’t know the exact amount of money spent on the treatment of breast cancer. The reasons they gave were: (1) They are not the ones that pay for the full treatment. They get financial support from family members, relatives and religious bodies. So they can’t tell the exact amount spent. | *“I don’t know the amount of money spent on the treatment because my brothers are the ones paying for everything”.* | It is difficult for me to estimate direct costs, I did not pay for it but my brothers | The eastern patient is not in charge of paying for treatment and therefore cannot estimate it because the family takes care of it |
|  |  | *“I don’t know the exact estimate of the financial expenses because everything is done by my relatives”.* | It is difficult for me to estimate the direct costs; I did not pay for it but my family did. | The eastern patient is not in charge of paying for treatment and therefore cannot estimate it because the family takes care of it |
| **Japhet (2019)** | (2) Some attributed their reasons for not knowing the exact financial cost to lack of record of all expenditures. All the respondents did not record financial expenses. | *“I can’t tell how much I spent on this sickness because I don’t record the expenses”* | It is difficult for me to estimate direct costs; I have not recorded expenses | Constructs without relevant content to answer research questions |
| **Japhet (2019)** | (3) Some attributed it to lack of formal education. | *“My lack of western education makes me not able to record expenditure”.* | My non-Western upbringing hinders me from recording the expenses of treatment | The eastern patient is not in charge of paying for treatment and therefore cannot estimate it because the family takes care of it |
|  | Despite the fact that all of them don’t know the exact amount of money spent on the treatment and other logistics but they were able to give a fair estimate when further probed. They emphasized that the values given are not the exact amount but a fair estimate of the direct cost of treatment. All of the respondents voiced out concern that the treatment is very costly. | *“Everything about breast cancer is very expensive. From the investigations to buying of drugs, everything is just expensive”* | Everything is very expensive from examinations to treatment | Incurring medical expenses (examinations and treatment) |
| **Japhet (2019)** | When further probed, respondents took time and estimated various amounts of money spent treatment ranging from 553 USD to over 2,766 USD. Fauziya who was diagnosed of breast cancer a year ago reported the least amount of money spent the treatment. | *“For a rough estimate, I will say that I spent nothing less than 200 thousand naira of which I spent 70 thousand naira on chemotherapy alone”.* | I spent 200,000 naira, 70,000 just for chemo. | Incurring medical expenses (hospitalization, medication) |
| **Japhet (2019)** | Another woman who spent about 6 months on hospitalization revealed that: | *“I spent more than 500 thousand naira in federal teaching hospital within 6 months, apart from what I spent in state specialist hospital Yola”.* | I spent 500 thousand naira in 6 months operating hospitalization | Incurring medical expenses (hospitalization, medication) |
| **Japhet (2019)** | Furthermore, one woman who have been diagnosed two years ago and hospitalized for 7 months revealed huge expenses. | *“I spent more than a million naira on this treatment. I spent about 33 thousand naira in one day just for test (investigation) in federal teaching hospital alone (apart from what is spent at home before hospitalization). I spent, more than a million, (she emphasized) because I have been buying drugs for 18 months now”.* | I spent 1 million narnia | Incurring medical expenses (hospitalization, medication) |
| **Japhet (2019)** | Still on the direct cost, respondents reported selling one or more different types of properties ranging from land, cows and farm produce. One woman sold land to raise money for treatment | *“I sold my land just because of the treatment. I have suffered” (she added).* | I sold my land to support treatment | One sells one's agricultural resources land, animal products (which one would not have wanted to sell) to meet the expenses of treatment) |
| **Japhet (2019)** | Another woman reported selling of farm produce in order to raise fund for breast cancer treatment. | *“I sold farm produce (beans and groundnut) that I intended to store but the financial burden of this diseases make gave me no choice”.* | I sold my agricultural products to support treatment | One sells one's agricultural resources land, animal products (which one would not have wanted to sell) to meet the expenses of treatment) |
| **Japhet (2019)** | Some of the respondents reported selling of animals they rear for profit. It should be noted that these women sold these animals without the intention of selling at such time. | *“I sold my goat in order to raise money for my treatment”* | I had to sell my animals to get money | One sells one's agricultural resources land, animal products (which one would not have wanted to sell) to meet the expenses of treatment |
|  |  | *“My brothers sold 3 cows to raise money for my treatment. We have spent everything we have”.* | I had to sell my animals to get money | One sells one's agricultural resources land, animal products (which one would not have wanted to sell) to meet the expenses of treatment |
| **Japhet (2019)** | Apart from selling properties, the financial burden of breast cancer treatment forced some women to borrow money in other to augment what they have financially. | *“I am a civil servant but there are times that I borrow money because my salary alone cannot sustain this type of treatment”.* | To increase resources, I had to take out a loan |  |
| **Japhet (2019)** | **Indirect cost** The indirect cost is the effect of breast cancer on women’s occupation. Breast cancer affects respondents’ occupation and sources of income. In the sense some cannot continue with their normal business or even to go to work properly. | *“I used to farm and take care of my small garden near my house which usually fetch me money, but since the onset of this sickness, I can’t do that again”.* | My financial distress is indirectly caused by the fact that I can no longer work | Indirect costs are due to the inability to work |
| **Japhet (2019)** |  | *“I use to sell African bean cake but I cannot do that anymore because of my disease condition”.* | I cannot do my work because of illness | Indirect costs are due to the inability to work |
| **Japhet (2019)** | Another woman who is a civil servant reported that breast cancer affects her job. The sickness hinders her from attending some workshops and other functions or special assignments that could have earned her money. Furthermore, lack of punctuality at place of work is causing financial problems to her as her employers are not happy with her. | *“I don’t go to work properly as before especially when am on chemotherapy”.* | I can't work while doing chemo | Indirect costs are due to the inability to work |
| **Nolan (2019)** | **Actively managing spiritual self** Young AA survivors perceived their lived experience to be one that was uniquely continuous and spiritual in nature, requiring active management of spiritual changes to oneself. Participants learned to give greater attention to positive experiences of spiritual changes and used them to assist in pushing through or accepting their new selves. The spiritual self greatly impacted how the young AA survivors experienced life after breast cancer. PT 02 said, ‘You just see God afterwards.’ The following five experiences reflect participants’ management of spiritual self: hopefulness, life purpose, positive/spiritual change, religious/spiritual activity, and uncertainty. |  | I learned to value moments of spiritual growth, using them to accept my new life after cancer. This inner transformation has changed the way I face each day. | Constructs without relevant content to answer research questions |
| **Nolan (2019)** | **Uncertainty** Young AA survivors experienced concerns about their ability to return to some sense of normalcy and uncertainty about the future. PT 01 said, ‘How am I going to…you know, if I ever go back to work, how am I going to successfully work an eight-hour job that pays well enough to, you know, my bills, pay my insurance, things like that?’ At the same time, faith kept survivors positive: ‘On some days I worry…God, what am I supposed to be doing? But at the end of the day I’m optimistic’ (PT 08). Although questioning their ability to regain normalcy, young AA survivors sought to manage residual effects of treatment and actively manage changes to their physical self. |  | I experienced times of uncertainty about the future: getting back to normal, working, handling responsibilities. But faith helped me remain optimistic, even as I faced physical changes and tried to rebuild my life. | Uncertainty about the future |
| **Nolan (2019)** | **Actively managing physical self** Young AA survivors perceived physical changes to their bodies, which required active management on a daily basis. PT 03 observed physical changes were ‘reminder[s] of what you’ve been through.’ Participants developed strategies to manage changes over time, which lessened their focus on physical changes. They decided to push through or accept physical changes as part of a new physical self. This theme included eight physical experiences: nausea, appetite/weight changes, constipation, menstrual change/fertility, sleep, aches/pains, fatigue, and skin/hair changes. |  | My body has changed, and every day I have to manage the effects of treatment: nausea, appetite/weight changes, constipation, menstrual cycle/fertility changes, sleep, pain, fatigue, and skin/hair changes. At first they were a reminder of what I went through, but over time I found strategies to live with them. I learned to accept them as a new part of me. | Constructs without relevant content to answer research questions |
| **Nolan (2019)** | **Menstrual change/fertility** All expressed that their menstrual cycles had changed by way of having amenorrhea, changes in flow, and/or increasing intensity of pain. Women who were taking Tamoxifen to reduce risk of cancer recurrence also experienced menopausal symptoms such as alterations in vaginal sensitivity/secretions, hot flashes, and night sweats. Those experiencing amenorrhea remarked that being in menopause at a young age challenged their perspectives of their age. PT 12 (age 33) said, ‘My best friend is 45 and she’s just now hitting the hot flash stage. So, we laugh and cut up about [the fact that I have them too].’ Also, changes in menstruation led some participants question their ability to become pregnant. Childbearing concerns led to the desire to think about alternative means of childbearing such as fertility assistance, surrogacy, and adoption. Some young AA survivors reported that their healthcare teams were fertility advocates, despite these women saying that they were ‘not entertaining [having children]’ at the time (PT 04). Participants explained that healthcare providers did not acknowledge the challenges of childbearing when one is single (e.g. lack of a partner and financial constraints for fertility treatments). |  | After cancer, my body has changed: irregular cycle, menopausal symptoms and fertility concerns. I consider options such as egg preservation or adoption, but the practical and emotional challenges are greater to face when you are on your own, even from a financial standpoint. | Coping with changes caused by cancer is also a challenge from an economic perspective |
| **Nolan (2019)** | **Aches/pains** Young AA survivors experienced aches, pains, numbness, and muscle tightness frequently. PT 03 said, ‘It’s pain[ful] at times and sometimes numbness,…, and the main part is under my arm where they slit into it.’ The aches and pains at surgery incision sites and the extremities were intermittent and associated with positioning. Those with aches and pains had difficulty with performing daily activities (e.g. sleeping, doing physical activity, and working). Yet, young AA survivors remained active. They learned to be self-aware of limitations and pre-medicate before activity. |  | I live with intermittent pain, tingling, and muscle stiffness, especially in the operated areas. Sometimes they limit my daily activities, such as work, but I've learned to manage them with self-awareness and preparation before physical exertion. | Side effects that impact work activity |
| **Nolan (2019)** | **Actively managing psychological self** Participants perceived that breast cancer survivorship was a continuous state that required one to actively manage psychological changes to oneself. Participants described psychological changes as an ‘emotional roller coaster’ with good days and bad days (PT 02). This theme included five experiences: anxiety/distress and depression, fear, guilt, cognitive changes, and overall perception of QOL and satisfaction. |  | I experience cancer survival as a psychological journey to be managed day by day, with emotional ups and downs. I find myself having to deal with anxiety/discomfort and depression, fear, guilt, cognitive changes, and general perceptions of quality of life and satisfaction. I can't find any direct links to fintox, although they are there in theory. | Constructs without relevant content to answer research questions |
| **Nolan (2019)** | **Anxiety/distress and depression** Young AA survivors reported challenges with managing their emotions. They experienced ups and downs, which they related to living with stressors of survivorship in addition to ‘normal’ circumstances (PT 02). Stressors included ability to meet financial obligations, work, and maintain adequate insurance coverage; physical changes; establishing/maintaining partnerships; uncertainty of fertility and ability to breastfeed; preserving a sense of normalcy; and experiencing fear. However, participants stated that they had to move  past their stressors and should ‘be thankful that [they were] blessed with life’ (PT 04). In addition to feelings of anxiety/distress, they reported feeling down or depressed. At the same time, they acknowledged the importance of refraining from negative thoughts feelings and learning to cope. | *‘The biggest hit that I’m experiencing right now is financial. I have medical bills, that I accumulated during treatment, still today. I did have private insurance, but my insurance did not cover everything and so um, I do still have some medical bills looming. I feel like at 40, I am just trying to secure some stability. I have absolutely had more stability in my life in the past. I feel like I’m like starting all over from scratch.’ Participant 08* | The biggest challenge now is financial: despite insurance, medical debts haunt me and I struggle to keep up with my financial obligations, work and maintaining adequate insurance coverage. At 40, I feel I have to try to regain the stability I used to take for granted. | Health debts that haunt |
|  |  |  |  | Difficulty in maintaining adequate insurance coverage |
|  |  |  |  | Difficulties in managing work |
| **Nolan (2019)** | **Guilt** Young AA survivors also experienced psychological concerns with guilt. A few survivors remarked that they had experienced ‘feeling guilty about surviving’ or living longer than others (PT 08). They questioned why they were spared, while their friends were not. Guilt was also associated with needing assistance from others. PT 01 said, ‘I just don’t want to put [taking care of me] on nobody else if I don’t have to.’ Young AA survivors ultimately did not want to burden other people. | *‘It bothered me a lot because she had to walk away from her job, and I felt guilty because she has always been very independent. It hurt me because I felt like I put her in the situation [losing her job], and she always tell me ‘don’t feel bad’ because I’m her child.’ Participant 04* | I feel guilty for forcing my mother to quit her job to assist me. It is hard to accept that I have limited the independence of my loved ones, even though they tell me not to. | Guilt over family members' lifestyle changes |
|  |  |  |  | Job changes of family members affecting earnings |
| **Nolan (2019)** | **Cognitive changes** Many young AA survivors experienced annoying to ‘frustrating’ and ‘scary’ changes in their mental abilities. PT 01 said, ‘It takes me longer to understand it than it used to.’ Only one survivor denied cognitive changes; of note, her treatment did not include chemotherapy or hormonal therapy. Those affected with cognitive changes frequently described losses in memory and some degree of changes in concentration and multitasking. PT 14 said she frequently needed to double check herself, particularly on tasks related to her children. PT 03 said that her ability to concentrate on her hobbies had decreased. Other difficulties perceived lengthening of information processing and mood changes. Cognitive changes affected their work, home activities, and interactions with others warranting young AA survivors to acknowledge the presence of these changes. |  | I notice frustrating changes in my cognitive abilities that frighten me: I can't handle memory, concentration and multitasking demands like before. Sometimes I have to recheck things several times, especially when it comes to my children. These changes affect my work, daily activities, interactions with others, and even my hobbies. | Side effects that impact work activity |
| **Nolan (2019)** | **Actively managing social self** Young AA survivors perceived that breast cancer survivorship was a continuous state that required one to actively manage social changes to oneself. Participants experienced social changes as ‘an adjustment’ for themselves and those with whom they interacted. However, young AA survivors were sensitive to maintaining strength in an attempt to shield others from undergoing adjustment to their lives. This theme included descriptions of five experiences: employment and financial burden, social support, personal relationship and role changes, sexuality, and isolation. |  | I experience cancer survivorship as a continuous social readjustment. I try to protect my loved ones from hardship, but I have to manage changes in work and finances, social support, personal relationships and roles, sexuality, and sense of isolation. | Managing work and economic aspects to protect loved ones |
| **Nolan (2019)** | **Employment and financial burden** The most commonly reported social concern was financial stability. As previously discussed, employment and financial burden were large psychological stressors. Participants remarked that residual medical bills, time away from work, and insurance coverage affected their ability to maintain their household responsibilities and added to distress. PT 08 said financial burden lowered her credit score and job security: ‘My credit has definitely taken a hit um, because of those medical bills.…My career and job opportunity has taken a hit because of my credit.’ PT 14 was her family’s ‘bread-winner.’ She worried about her household finances if she needed days off. Further, those who were unemployed/disabled had concerns about insurance benefits and finances if they were to re-enter the workforce. Yet, work was a stress reliever. PT 02 said, ‘ … going to work to me was like keeping myself going, keeping my head above water.’ Despite challenges, young AA survivors remained faithful that all would be well with faith and social support. | *‘I’m a single mom, raising two kids…just trying to keep everything afloat and trying to mentally stay, stay in the game, or stay in the race I would say…probably [doing] 65, 70% maintain my workload. We just gonna keep roling.’ Participant 08* | My social and family role requires me not to give up. I have to maintain 65-70% of my workload to stay afloat financially | Keeping the job allows the financial burden to be borne |
| **Nolan (2019)** |  | *‘I’m a single mom raising two kids, just trying to keep everything afloat and trying to mentally stay, stay in the game…It got to a point where I needed some time off because I was exhausted from doing all of that and maintaining my job full time. My employer worked with me, but it was still like, “okay, if you’re actually here at work, we see we see physically that you are enduring and things have shifted and changed, and you are absolutely going through what you’re going through, but you’re here at work, and you are probably 65–70% maintaining your workload.”’* | My reduced work performance (65-70%) threatens my economic stability, while care needs conflict with work demands. My employer's support, although present, does not prevent the risk of financial toxicity. | Job performance threatens economic stability |
| **Nolan (2019)** | **Seeking survivorship knowledge** Young AA survivors perceived that breast cancer survivorship was a continuous state that required one to seek survivorship knowledge about how to manage life after a breast cancer diagnosis. This knowledge answered questions about current survivorship as well as informed young AA survivors of potential residual effects and management strategies. This theme consisted of three experiences: personal experiences, others’ experiences, and healthcare and ancillary professional expertise. |  | Being a young survivor involves actively seeking knowledge about managing life in survivorship, especially about possible late effects and strategies for managing these through three spheres: personal experiences, those of others in the same condition, and those of health care professionals | Constructs without relevant content to answer research questions |
| **Nolan (2019)** | **Personal experiences and others’ experiences** Young AA survivors described the transition from patient to survivor as difficult, leaving them with many questions about how their new lives would unfold. Both passive (learning from experience) and active (asking for recommendations) learning took place. Young AA survivors remarked that experiencing changes caused them to find and utilize coping strategies such as spiritual activities, talking with others about breast cancer and survivorship (though it reminded them of the hardships of their journeys), positive affirmations, laughter, avoiding triggers, taking prescribed medications, seeking financial assistance from family members and organizations, and accepting changes to self. PT 10 regularly attended counseling sessions to ‘make sure [she was] still okay.’ These coping strategies strengthened their resolve to continue to move forward in life and survivorship. |  | The transition between being a patient and becoming a survivor is not easy and it takes time to learn how to manage the ‘new life’. I had to learn how to seek financial assistance from family members and organizations | Learning to ask for support from others |
| **Pisu (2019)** | Regarding the content of the cost of care (CoC) conversation, survivors highlighted 2 main elements: 1) reassurance: survivors expressed the need to be reassured about the ability to receive treatment regardless of cost, and 2) action: survivors expressed the need to include a discussion of payment options or resources available, so that it would be easier to afford what they perceived to be life-saving treatment (Table 2). Conversations should start with acknowledging patients' vulnerability, with such comments as “I know this is a difficult time for you” or “You've been diagnosed, and we know this is a traumatic situation for you; it's going to be difficult.” This introduction would be followed by a statement of reassurance, such as “But here are some resources to help ease your stress level.” The plan of action could include a discussion of payment plans to make it easier to pay for treatment, or an explanation of resources or financial assistance programs to which survivors could apply. Several survivors emphasized they did not want to hear that there was no help or support available or that the purpose of the CoC conversation was to collect money (Table 2). Survivors generally believed that the best timing for the CoC conversation was after the visit in which they are told about the diagnosis but before treatment starts. One reason was because at diagnosis patients feel highly vulnerable and overwhelmed and would not be in a condition to fully appreciate cost information. Moreover, this information was felt to be more relevant once the treatment plan was established. Several participants also thought that the CoC conversation should be done when patients are ready for it or even initiated by patients, but should not be “forced” on them (Table 2). Most survivors agreed that professionals, such as social workers, billing specialists, or financial counsel ors, would be the most acceptable personnel to discuss CoC. Many stated that they would not want physicians to discuss CoC, because these providers should focus on the treatment and they would have neither the information nor the time. Some also thought that patients would be embarrassed to talk about affordability problems with the doctors. The person charged with discussing CoC should be kind, compassionate, honest, up-front, and understanding of the vulnerability of cancer patients soon after diagnosis. He or she should be available to assist patients when and if needed. Moreover, this person should be prepared and competent, be able to combine cost information with the details of the patient's insurance plan and prescribed treatment, and be knowledgeable of resources available. One survivor also suggested that 2 people do the CoC conversation: one with expertise regarding insurance and the other who would facilitate access to available resources. | ***Content Reassurance*** *I would like that person to be reassuring. Because, if there was some reason that I could not cover all the cost and would have problems with that, to let me know that there were options available to it. I would have liked someone to have sat down with me, and said, “You've been diagnosed, we know this is a traumatic situation for you, it's going to be difficult. But here are some resources to help ease your stress level.* ***Action*** *I would hope somebody would be able to sit down with me and say . . . We’re going to find a way to pay for it rather than me feeling like I had to choose between having treatment or not.* ***Do not want to hear*** *I'm sorry. We don’t know how to help you pay for that. We don't know what you're going to do. Then I would go home and I would worry about well, I can't afford this. How am I going to have it done? I'm just going to die from cancer? I wouldn't want her to tell me that.  I wouldn't want them to say, “Will that be MasterCard or a personal check?”* | I would have needed a person to reassure me of the options available to pay the fees | Need to have a case manager of the economic aspects |
|  |  |  | I would have needed reassurance that there was a chance of being cured despite the cost of treatment | Need to have a case manager of the economic aspects |
|  |  |  | I would have liked to talk to a professional dedicated to economic aspects who was not a health professional | Need to have a case manager of the economic aspects |
|  |  |  | I wish I had known what options were available for stress relief | Need for information support to limit stress |
|  |  |  | I would have liked to discuss payment options and financial assistance programs to pay for treatments | Need to have a case manager of the economic aspects |
|  |  | ***Timing After diagnosis*** *Maybe a week after diagnosis or something. Because diagnosis is so overwhelming in itself and I don't think some people can think past the fear of the unknown actually.* ***When patient is ready*** *If the person was to walk in and say, “I know this is a difficult time for you, and I know you might have a lot of questions for your doctor, and the nurses, or the oncologist. But you might have some financial questions that you need to ask, and I'm here to answer your questions and if this is not the right time, here's my number, my name and number, you can call me at any time and we can discuss the cost. But when you're ready to talk to me—I'm not going to be here to force myself on you, it's when you're ready, and hopefully go from there.* | I didn't want to hear that there was no possibility of support | Need for information support to limit stress |
|  |  | ***Person Not the physician*** *What would my reasons be? Because I want the physician to deal with getting that cancer out of me. So, this part is the business part.* | I needed to have a point person to talk about the financial aspects once I was ready to talk about it | Proper stage in which to discuss economic aspects |
|  |  | ***Characteristics Have time*** *I think that they [figures like financial advisors] have the time. That's what their job is, to talk about the finances, and the cost of cancer. The cost of what it's going to cost me and my family. I have the time to really be able to talk to them about it. I do not want to waste the doctor's time about that or the nurse's time.* ***Compassionate*** *I don't know if you know XXX [in the business office], but she's kind. She's compassionate, and she gives you all of the information you need to know in a direct fashion, somebody like her.* ***Good communicator*** *Compassionate. Right. Has training on how to communicate well and understand that they're dealing with somebody who has just been hit with it.* ***Knowledgeable*** *They should hire somebody full time to do nothing but discuss cancer care. They should know what kind of insurance you have, what your insurance is expected to pay, and by the time you get there on that first visit after your surgery, they should be able to give you some type of idea of what you're cost is going to be, or what your cost is going to be . . . they should have discussed and seen your chart to know enough to know what type of treatment you're going to have, how many times you're going to need to be coming back, what your copays are going to be.* | Conversations about treatment costs should take place after diagnosis and before treatment has begun | Proper stage in which to discuss economic aspects |
|  |  |  | Having an empathetic person to inform me about financial aspects | Need to have a case manager of the economic aspects |
|  |  |  | I would have needed a person who was knowledgeable about my clinical pathway and informed about the costs associated with my treatment | Need to have a case manager of the economic aspects |
| **McEwan (2014)** | **Public Policy Factors Low economic capacity, inefficiency of the public sector** The national cancer institue is the only hospital in the country that offers free cancer treatment to adults. Among 10 patients who went to the National Cancer Institute none was referred through an established referral system. Instead they heard about the NCI through interpersonal networks or were advised by private practice doctors when they disclosed that they could not afford the treatment fees. Other patients appllied for subsidized treatment through the ministry of health and population, which does not guarantee prompt or complete funding. Financial issues occasioned delay as with the following woman who thought she had cancer, but waited until she could afford treatment. | *Financially it was not possible. When God made it easy and the money came in, my husband said let's go! It was really a case of when the money came in and that was just in the beginning when we are waiting for that initial surgery* | When the money came in I was able to afford treatment | Possibility of treatment only when you have financial availability |
|  |  |  | I was not aware that there was a center that offered treatments for free | Lack of knowledge of centers offering free treatments |
| **McEwan (2014)** | Navigating the healthcare system was a daunting experience. The complexity and disorganization of the healthcare system, the lack of information about which type of doctor to see, whether the doctor/hospital was aligned to their ability to pay, and women's lack of trust in the medical profession caused significant delays. Patients who were able to find free treatment still faced difficulties paying for transport costs to and from the treatment center. | *Dr. A tells me to have the nodes analyzed. So I go to Dr. B., and she says instead of cutting away at your body, do an MRI… Dr A. tells me cancer; the MRI people tell me it is nothing. So what do I do?* | Constructs without relevant content to answer research questions | Constructs without relevant content to answer research questions |
|  |  | *I went around looking for doctors, as I didn't know anything about this subject. Nor did I know what doctor to go to; should he be a surgeon or a medical doctor? All these things I knew very late.* | Constructs without relevant content to answer research questions | Constructs without relevant content to answer research questions |
|  |  | *I don't know why I didn't get the approval to have all my chemotherapy paid for. The hospital said it will get another approval for two-thirds, and I will pay a third. I told them I don't have that kind of money. Then one of them checked my ID and said, "You live in Old Cairo, next to the NCI. Go there!.* | It was proposed that I change hospitals because I did not have the money to pay for the chemotherapy co-funding | Changing hospital due to inability to support co-payment of treatment |
| **Klimmek (2010)** | **Interacting With Managed Care Organizations** The absence of responsive, human connections at the MCO (Managed care organizations (MCOs) represent the most common form of private health insurance in the United States, with approximately 126.4 million enrollees in 2008 (HealthLeaders- InterStudy, 2008) level also led to perceptions of MCOs as fundamentally uncaring organizations. Many participants brought up areas where they found MCOs lacking in sensitivity, citing a perceived lack of humanity: “For [the MCO] to just look at the bottom line, these aren’t, these are human lives that we’re talking about. These are human—these are, these are people.” Participants were clear in understanding penalties for not completing MCO-required tasks, such as delayed approvals and higher nonreimbursed costs. One woman explained, | *I know when I was going through my biopsies, they’d constantly say, “If you don’t have [the referral] in time, we won’t treat you.” Because I guess that they feel like they won’t be paid. So I was—it made me fanatical about trying to be sure all my ducks were in a row.* | Lack of sensitivity on the part of U.S. private health insurers that they were dealing with people and not human lives. When faced with the tests I had to go through, they would give me bureaucratic fuss instead of getting things done immediately. | Insurance: lack of humanity in paperwork management |
| **Klimmek (2010)** | **Obtaining Authorizations** Many of the interviews focused on participants’ stressful experiences related to obtaining authorizations for medical care from MCOs. The challenges of authorizations included perceptions of lengthy waiting periods and refusals for authorization for certain treatments, specialists, or diagnostics. Many of the participants had difficulty separating an MCO from their cancer care, believing the financing of such expensive treatment regimens to be nearly as important as the cancer itself. A few women reported feeling angry or otherwise disturbed at their perceptions of MCOs influencing care decisions with little or no understanding of the true nature of their care needs. In one woman’s words, “The insurance company put their nose in things where they have no business.” Another stated, | *For the insurance company to second-guess your physicians who know you, who have seen you, is absurd. It, it just really makes me upset and really makes me angry. That someone who doesn’t know your history, who doesn’t know what’s going with you, has not followed you through all of this, can say “yes” or “no.”* | Insurance companies allow themselves to make decisions about my care without knowing my history, overriding doctors and my privacy | Insurance: lack of respect for privacy in file management |
| **Klimmek (2010)** | The bureaucracy and lack of predictability inherent in many MCO processes (e.g., referrals, authorizations, billing issues) led to extended waiting on the part of participants, which, in turn, became a source of frustration. Cancer-related appointments were believed to be dependent on MCO approval, and bills could not be paid until MCO determination of coverage and payment had been made. Both delays added stress and anxiety on top of worries related to the cancer diagnosis itself. MCO limitations often were unrealized until after treatment completion, hampering women’s ability to make educated treatment choices. |  | My ability to make an informed choice of treatment has been affected by not knowing the limitations of insurance | Informed decisions on treatment options |
| **Klimmek (2010)** | **Paying Bills and Planning for the Costs of Care** The challenges of paying bills and planning for future costs while receiving or recovering from cancer treatment were a constant and consistent theme throughout the interviews. Women shared the emotional distress they experienced as a result of the lack of transparency around billing procedures and their inability to predict out-of-pocket expenses. To some, bills represented the antithesis of the support they felt they should be receiving during a time of great vulnerability, as described by two women. | *They were nickel and diming me. . . . I can remember even crying one day when I was writing a check. I was like, I’m just tired of writing these checks, I said, every time I turn around it’s for something. You know, and—just very emotional about it. I can remember vividly.* | The uncertainty and economic vulnerability made me cry every time I wrote a check | Emotional distress related to paying or organizing to reduce costs |
|  |  | *The worst [part of treatment] was, is the bills coming in . . . when they send you these little nasty-grams saying, “Okay, —this hasn’t been paid; we want you to pay it.”* | Worse than the treatments were the bills | Emotional distress related to paying or organizing to reduce costs |
|  | Women spoke about seemingly random changes related to insurance coverage of their treatment plans or prescriptions, which contributed significantly to emotional and financial stress. Some of the stress arose when changes in insurance plans resulted in providers they had been seeing suddenly becoming “specialists” and therefore requiring higher copayments. In the words of one woman, “And now they’re specialists. That means I have to pay a copayment of $30 instead of the $15 I was getting because I was only referred. So all of a sudden they’re now specialists.” Participants also struggled to make sense of rules governing the classifications and costs of their prescriptions. | *I didn’t appreciate the fact that, if I had gone up, if [capecitabine] were a drug that could be delivered IV, they’d be paying for most of it, almost all of it. But because it’s a drug, in fact, somebody told me that—it was delivered IV, they used to use a pump for it when it first came out. So the fact that it’s a pill, I have to pay $600 every six weeks, and that part of it bugs me. It doesn’t make sense to me, because they don’t have to hire any employees to deliver it, I don’t take a room upstairs, but because it’s a pill form, I have to pay through the nose for it. That part bothers me.* | For the receiving refunds I should have done heavier treatment, not the fairer ones, this makes me angry | Anger at insurance companies that do not weigh the good of the person but cover treatments |
| **Klimmek (2010)** | Participants’ perceptions that they received less reimbursement for lower-level procedures, which appeared to them to be more cost-efficient for providers, also resulted in anger and confusion. Two women reflected on how they felt that their choices to undergo lumpectomies, rather than full mastectomies, ended up costing them significantly more money out of pocket than if they had chosen to have the more invasive procedure, which would have required overnight hospitalization but might have been covered in full by their insurance providers. “If I would have had a mastectomy and I would have had to spend a night in the hospital, they would have paid for everything. But since mine was a lumpectomy and it was done as outpatient, they didn’t pay for it.” Another woman, who became aware of the cost differential after she already had had the procedure, concluded that if she had known how much the lumpectomy ended up costing her she probably would have had a full mastectomy. | *Finding out about the high out-of-pocket treatment costs was like somebody deflated my balloon. It was, it was an unpleasant surprise. It was: Should I have just had the mastectomy, and it would have all been paid for because that was an option? Yeah. I still think that . . . but it would have all been paid for. If I would have known, I would have probably had the mastectomy done.* |  |  |
| **Klimmek (2010)** | For some participants, the lack of clear information about costs prior to the initiation of treatment was considered a form of provider irresponsibility and neglect, resulting in a decreased level of trust in both the MCO and the healthcare system. Participants who had identified errors in bills they received from their MCOs described how the experience caused them to adopt a state of constant vigilance over all medical bills, expending considerable time scrutinizing every bill received. Finally, participants described how concerns about the financial welfare of their families added to their sense of burden. Some described their frustration with the inability to plan for their families’ futures, as well as fear of being unable to “leave something behind” in their estates for loved ones. |  | I live with the fear of being screwed over by insurance and not being able to leave anything as a legacy | Emotional distress of being ripped off by insurance companies |
|  |  |  | I live von the fear of being screwed over by insurance and not being able to leave anything as a legacy | Emotional anguish of not being able to leave anything as a legacy |
| **Klimmek (2010)** | **Difficulty Obtaining Assistance With Insurance-Related Tasks** Despite the numerous insurance-related challenges participants confronted during cancer treatment and the early stages of survivorship, most expressed a sense of bearing sole responsibility for managing the issues. Some women saw tasks related to their insurance providers as impossible to delegate to others. Others described their independence as a result of their attempts to negotiate their autonomy against the role of “being sick.” This approach resulted in some participants experiencing burnout as a result of juggling managed care tasks in addition to the work of cancer treatment and survivorship. | *Having cancer is a big job. . . . I’m a pretty strong person, I think. . . . You try not to let it overwhelm your life. It does, no matter how you look at it. And, so, you’re trying to do your best to take care of yourself. To me, there’s been two parts to my cancer: there’s been the physical; there’s been, you know, all the physical issues of it, and the paperwork issues. . . . It shouldn’t be that complicated to get the treatment I need.* | I cannot delegate insurance aspects to anyone and it sends me into burn out. I have to fight to get my care | Emotional distress related to insurance aspects |
| **Klimmek (2010)** | Some participants also shared how they had waited to ask for assistance with MCO-related tasks until they were too exasperated or fatigued. Those who were able to obtain assistance with the tasks expressed the tremendous relief they experienced to have a bit of that burden lifted. | *Yeah, it just wasn’t something I could have done on my own. . . . The office upstairs [in the cancer center], they’re so good, I think, about dealing with insurance. So it’s kind of like it’s taken off my shoulders. I know they’re going to take care of it. . . . If I didn’t have that support, it would be very, very difficult I think.* | Dealing with the insurance aspects exasperated and fatigued me. When I asked for help to follow up on these aspects, I felt relieved | Emotional distress related to insurance aspects |
| **Darby (2009)** | Thematic analysis revealed three salient themes around the financial impact of participants’ cancer experiences. The first theme was the lack of access to adequate care if one is unable to pay or is without adequate health insurance coverage. In the current study, a majority of the women agreed that access to equal care and financial concerns added extra stress throughout their cancer experiences. Women expressed the opinion that their insurance status indicated the level of care they received. Many women perceived that their treatment would have been different and better if they had had adequate insurance or more financial support. | *I had knots on my breasts and under my arms. I knew it wasn’t normal but my doctor wouldn’t give me a mammogram because I didn’t have any nsurance to pay for it.* | My doctor did not prescribe a mammogram because I did not have insurance to cover the expense | Lack of access to adequate care |
|  |  | *I’m on TennCare [Tennessee’s Medicaid] and asked for a second opinion. My doctor asked, “Why are you going after a second opinion? I already told you everything.” . . . the type of insurance you have seems to dictate the type of treatment you get . . . .* | My type of insurance affected the type of treatment I could access | Insurance: treatment choices dictated by insurance type |
|  |  |  | If I had different insurance or more financial support I might have received different medical treatment | Insurance: treatment choices dictated by insurance type |
|  |  | *Society has left us no choice. Either you pay or you die, because without insurance the doctors are not going to see you.* | I felt left behind/abandoned by society because I didn't have insurance and couldn't pay for treatment | Feeling economically abandoned |
| **Darby (2009)** | For the women in our focus groups who had inadequate or no insurance, the out-ofpocket expense for a $15 co-pay often resulted in missed treatments. | *And if you don’t have your co-pay, a lot of times they say, “Well, we can reschedule. When is best for you?” meaning “When you have the money, then you can come back.”* | My co-payment was necessary to start the procedure | Insurance: a co-payment was required |
|  |  | *I was told they needed my co-pay, so I had to explain to them, “I don’t have a co-pay, I don’t have a job.”* | I did not have the ability to co-finance treatment because I did not have a job | Insurance: having a job affects the ability to co-pay |
|  |  | *I owe over $1,000. They call every day and say “You’ve got to pay something, or we won’t treat you anymore.”* | I received pressure from the insurance company for me to pay | Insurance: they put pressure to receive payments |
| **Darby (2009)** | The second theme was the long-term financial burden of this disease from the out-of-pocket expenses incurred by these women and their families during and after their cancer experiences. Women who had inadequate or no income and/or no health insurance struggled with how to pay for needed services, while supporting themselves and their families during treatment and into long-term survival. Several women spoke of being the sole support for herself or her family and her fear of being unable to care for her children, or to keep her job and insurance coverage. The financial impact of the cancer was felt long after the treatment phase was complete. | *I wiped out my savings taking care of me. My son paid when I no longer had a savings. I thought I was saving to retire early, but I didn’t realize that it was going to be paid to save my life. But I was fortunate I had it to pay, before it ran out.* | I have exhausted my savings for treatment | Depletion of savings, assets, pension funds |
|  |  |  | My son supported me financially | Financial support from family members |
|  |  |  | I had planned to use the savings to retire early, but instead I used them to treat myself | Change of future plans |
|  |  |  | I was lucky to have savings to be able to take care of myself. | Savings that enable care |
|  |  | *I was still an employee, and when the doctor turned me loose to go back to work, that’s when they wouldn’t let me come back. I worked for 17 years . . . worked hard; worked smarter . . . they wouldn’t let me come back.* | I lost my job | Losing your job |
|  |  | *I am trying to get disability. I can’t work right now. Doctor’s orders. I want to work. I have worked all my life.* | I tried to access disability because I was unable to work | Attempt to access disability due to inability to work |
|  |  | *My job won’t wait on me to feel better.* | I had the perception that work was not waiting for me to get better to return | Job changes |
| **Darby (2009)** | In several instances women discussed their fears and anxieties of becoming homeless or losing their children because they were struggling financially or physically to provide for their children’s well-being. | *It makes you homeless. You work for years, years, and years and just because I have cancer, I am homeless. That’s not fair. That’s just not right.* | I lost my house to cancer | Difficulty in maintaining possession of real estate |
|  |  |  | Feelings of injustice toward the consequences of cancer in everyday life | Constructs without relevant content to answer research questions |
|  |  | *I’m about to get put out. Probably the end of the month . . . because I had to pay my light bill . . . and you got to feed the kids . . . you have to stay at the shelter before you get Section 8 housing.* | I was on the verge of losing my home and my children | Difficulty in maintaining possession of real estate |
|  |  | *They take away the insurance, unemployment and everything! I have nothing left.* | Everything was taken away from me | Deprivation of personal property |
| **Darby (2009)** | The final theme was the direct, nonmedical expenses that include such items as childcare, housekeeping, home care, wigs, prostheses, over-the-counter medications, and expenses associated with travel. For those individuals who have health insurance, non-medical items are rarely covered. Cancer insurance and long-term insurance policies are available and help reduce the unforeseen out-of-pocket expenses that create financial burdens for many families who have health insurance. Health insurance supplements are only beneficial to the population that can afford to pay the additional premiums. | *When I was taking my treatments, my car broke down and I didn’t have any way to get to the center. I finally got a bus card because I couldn’t afford to fix the car. Other than the bus, I don’t know how I would have got to treatment.* | I couldn't afford to fix the car. | Difficulty in maintaining possession of real estate |
|  |  |  | I was given a voucher to use public transportation so I could go for treatment | Receive vouchers to pay for parking |
|  |  |  | Non-medical expenses are not covered, even though I had insurance | Insurance: limited health coverage |
|  |  |  | Supplements to insurance helped me but came at a cost | Insurance: supplement with paid supplements |
|  |  | *I had insurance, but not enough.* | I had insurance but it was not enough | Insurance: limited health coverage |
| **Darby (2009)** | As these women shared their cancer experiences of the financial, physical and emotional toll it took on them, there was a common underlying theme of feeling “alone and lost.” |  | I felt lonely and abandoned | Being on your own with costs |
| **Lauzier (2005)** | **Patients’ and caregivers’ conceptualization of costs** Extent of Costs.  When asked specifically, most patients reported that breast cancer could be costly or even very costly for the affected person and her family, but a few said this was not their own particular experience. Several caregivers echoed this perception, although some specified that costs for the caregiver considered as an individual were not very high. Several patients and caregivers pointed out that the extent of costs was linked to each person’s context, which can differ considerably from case to case. They identified characteristics likely to affect the nature and extent costs. For instance, some mentioned that breast cancer had been costly because the patient had had several medical complications, because she did not have salary insurance or because she had had to travel extensively to receive treatments and meet specialists. For other participants, breast cancer had not been costly because their out-of-pocket expenses were covered by government programs or because they were helped and supported by their family and friends. Participants sometimes linked impact of costs to a person’s or family’s situation and several participants compared the situation of families who were financially well off with those who were in a more precarious situation (Box 1, quote 1). | *‘. . . I can’t imagine someone who lives alone, who has neither friends or spouse, who lives in the country and who comes here [Montreal] all alone and doesn’t have any insurance. Well, I realized that it must be hell. I don’t know how such a person could cope with things. Quite simply, I think it’s impossible, unthinkable.’ (PM)* | I did not feel the financial burden of the disease because I had the support of government programs to meet expenses not covered by insurance and the support of family members | Economic support of government programs |
|  |  |  | I did not feel the financial burden of the disease because I had the support of government programs to meet expenses not covered by insurance and the support of family members | Financial support from family members |
|  |  |  | The illness was expensive because I had to travel a lot to undergo treatment | Incur non-medical expenses (transportation, gasoline) in order to get treatment |
| **Lauzier (2005)** | **Costs substantial, but not the most stressful aspect of the illness.** Even though most participants agreed that having cancer could be expensive or very expensive for the patient and her family, several mentioned that money was not their greatest worry during the illness. When they were first diagnosed and had to deal with treatments, all the energy and attention of the patients and their caregivers seemed to be devoted to fighting the disease. Several patients and caregivers stated that during this period they hadn’t even thought about the costs resulting from the cancer (Box 1, quote 2). For other patients, however, costs related to breast cancer were a worry and a source of stress in themselves. Subjective evaluation of the costs and ensuing preoccupations seemed to depend not only on expenses and lost wages per se, but also on the financial capacity to cope with them. | *‘I think it’s obvious that when something like this happens, you don’t think about money. You tell yourself that you’ll do what you have to do whatever it takes and you won’t give up. It’s your health that’s on the line.’ (CBC)* | When I got the diagnosis, I didn't immediately think about money | Thinking about the economic aspects was not immediate |
|  |  |  | Positive attitude towards the disease that positively influences the perception of financial toxicity | Positive attitude toward the disease positively affecting perception of financial toxicity |
|  |  |  | I became concerned because of the costs associated with the disease | Concern about not being able to pay the costs of the disease |
|  |  |  | My concerns were also due to the economic ability to be able to cope with the costs associated with the disease | Concern about not being able to pay the costs of the disease |
| **Lauzier (2005)** | **Unavoidable nature of some costs**.  Several patients saw some costs resulting from breast cancer as unavoidable. Examples of such costs are those related to getting to and from treatments, to the treatment and its side effects, and to job changes. Several said that they ‘had no choice’ and did not question certain expenses or the loss of salary. In comparison, other types of expenses were seen as avoidable and depended on ability to pay. Examples of these latter expenses were house cleaning, treats to the caregiver or to the patient, and massage therapy (Box 1, quote 3). | *‘[. . .] maybe we’re lucky or privileged to treat ourselves to trips, to get a nice little dress because well, OK I do pamper myself, but it must not be very easy for some people, well, they don’t have these expenses because they can’t afford them.’ (PQC)* | I feel fortunate to be able to afford expenditures on unnecessary goods | Keep spending on unnecessary goods |
|  |  |  | The costs associated with transportation to do the treatment, the treatment itself, and the side effects and work changes were unavoidable | Incur non-medical expenses (transportation) during treatment |
| **Lauzier (2005)** | **Family, not individual costs.** In most of the cases, cancer-related costs and their consequences seemed to be borne by the whole family, and not only by the patient. One patient stated, ‘I saw all that as a family expense’. The increases in out-ofpocket expenses and the patient’s wage losses could affect the whole family’s financial situation (Box 1, quote 4). These changes in financial situation sometimes resulted in families redefining their priorities for a certain period of time. For example, some families abandoned projects or various activities of family members were changed or cut back. | *‘I also found myself feeling financially insecure when at one point, I realized my husband was working longer hours. I felt insecure financially because I didn’t have any insurance. I found that, well, I was lucky, we’re doing fine. But there was an instability that crept into things in terms of costs and all that.’ (PM)* | I felt insecure financially because I didn't have insurance | Insurance: economic insecurity associated with lack of coverage |
|  |  |  | I found out that my husband had to work longer hours. | Pressure on partner/family due to financial stress |
|  |  |  | My family's plans and activities have changed or reduced due to the costs associated with the disease | Postpone/change future family plans |
| **Lauzier (2005)** | **Accumulation of costs and their effect over a long period.**  The period of time during which costs were incurred by some patients and caregivers was long. For example, treatments and visits to specialists spread out over several months with attendant costs for travel and medication. Lost wages and the use of savings to pay out-of-pocket costs for breast cancer represented losses that were difficult or impossible to recover, even when most treatments were over and the patient was healthy again. Also, some patients said they stopped working permanently or reduced their hours because of breast cancer, thereby reducing longterm revenue. This could weaken the family’s financial situation for a period extending well beyond that of diagnosis and treatments (Box 1, quote 5). | *‘I’m working half time, and that’s gonna be for the rest of my life. I had insurance but it ends this week. I can’t go back to the nursery full time to work with babies. So I took a part time job, so now I’ll only get half of my salary.’ (PQC)* | My insurance has a limited duration | Insurance with limited time validity |
|  |  |  | I had to reduce/half my working hours and now I only receive half my salary | Job changes affecting earnings |
|  |  |  | Savings and salaries have been used to pay for expenses associated with cancer, and it is difficult to recover this money, even after completing most treatments | Depletion of savings, assets, pension funds |
|  |  |  | Savings and salaries have been used to pay for expenses associated with cancer, and it is difficult to recover this money, even after completing most treatments | Recovering lost money |
| **Lauzier (2005)** | **Sources of costs Travel to and accommodation during treatments.** The patients and their caregivers stated that some costs relating to getting treatments could be sizeable and worrisome. Some participants had to shoulder costs for transportation, lodging, meals and parking when going for treatments and consultations with specialists. These costs began as soon as the disease was diagnosed and continued throughout the treatment and follow-up period. Several patients and their caregivers living in Baie-Comeau, an outlying area of the province, reported costs related to getting access to treatments not available in their own town. For some, these costs were the most worrisome costs of breast cancer. One woman who had radiotherapy in the US because of long waiting lists in Quebec received government aid which covered these costs. Some participants travelled by car, ferry and sometimes even by airplane to get to the city where radiotherapy or different specialists were available. Even participants living in or near cities where the full range of adjuvant treatments are available reported costs associated with getting to treatments. Accommodation for radiotherapy sometimes required sizeable expenditures since this treatment is generally administered on a daily basis for several weeks at a time (Box 2, quote 1). These costs also affected the people who accompanied the patients during their treatments. Finally, many people mentioned expenses for meals when they ate at cafeterias or restaurants. | *‘It cost $105 per week for accommodation but you also had to pay for the meals on the weekends. That brought the costs up to almost $200 per week, for 6 weeks. That meant $1,200 gone right there. I left for Rimouski on January 9 and got home on February 18.’ (PBC)* | I had to bear the costs of lodging, transportation, parking, and meals when I went for treatment or visits with specialists | Incur non-medical expenses (transportation, lodging, parking) during treatment |
|  |  |  | Expenses began immediately after diagnosis and continue throughout treatment and follow-up | Ongoing expenses from diagnosis onward |
|  |  |  | I had to incur transportation costs to access treatments that were not available in my city | Incur non-medical expenses (transportation) during treatment |
|  |  |  | Because of the long waiting lists, I had to go to another state to have radiation therapy | Non-medical expenses (travel to another state) to have treatment |
|  |  |  | In order to have radiation therapy or visits, I had to travel by different means of transportation, in addition to the car also the ship or the plane | Incur non-medical expenses (transportation) during treatment |
| **Lauzier (2005)** | **Medical treatments, psychosocial and alternative therapies.** The patients often had to undergo several types of treatments (Table 1). Even though costs of surgery, adjuvant treatments and medication given in hospitals in Quebec are covered by the government, patients mentioned that they had associated costs. For example, some patients had to take several sorts of medication to counteract the side effects of the treatments, had to buy an external breast prosthesis and more comfortable bras after the operation. Several patients who had radiotherapy had to buy creams, cotton bandages, clothes and bras because of the radiotherapy marks. Others who had chemotherapy treatments bought wigs, hats, scarves and makeup due to hair loss, and new clothes due to weight change. Some patients had taken other steps to deal with the disease and turned to psychosocial help and alternative therapies to deal with the effects of the disease and treatments. For instance, they consulted physiotherapists, massage therapists, psychologists and social workers. For some patients, these services were provided free of charge as part of comprehensive cancer care in a hospital or through a study in which the patient was a participant. In other cases, these costs were partially or completely covered by their personal insurance. However, natural products such as herbal remedies were paid for wholly by the patients. The cost of these products was quite considerable in some cases (Box 2, quote 2). | *‘I paid somewhere around $80 a month for all the vitamins and things I was using. That’s about how much I was paying.’ (PM)* | Even though the costs of surgery and adjuvant treatments were covered, I still had associated costs that in some cases were considerable | Insurance: limited health coverage |
| **Lauzier (2005)** | **Lost wages**.  For some patients, the greatest and most worrisome costs of breast cancer were those engendered by the loss of salary due to work absences. Because of breast cancer, all patients stopped work completely once or twice for periods lasting from 2 days to more than one year. Several participants had difficulty imagining how one could avoid being absent from work during and after treatments. Absences from work resulted from treatments, their side effects, and follow-up visits. Finally, some patients decided to permanently reduce the number of hours usually worked because of breast cancer. Length of the absences and, primarily, the individual’s working conditions (for example insurance, sick leave) were seen as the two factors contributing to the extent of wage losses. The absences from work of some patients were completely or partially covered by government benefits, the employers’ income insurance benefits or the income insurance benefits of selfemployed workers. Some patients also used paid sick leave, annual leave, overtime they had accumulated, or promised to pay back the time taken off by later working the necessary hours on an unpaid basis. Due to absences that could last a whole year, they sometimes combined different types of compensation. During certain periods, however, they were without any compensating income and they had to draw on personal savings. This was particularly the case for self-employed workers without income insurance. Not only did working conditions influence the extent of wage losses, they also played a role in the decision to take time off during the illness (Box 2, quote 3). Among employed caregivers, none took an extended period off work. Even though they did occasionally take time off work to accompany the patients, visit them, provide moral support and help them with domestic chores, the caregivers did not seem to have experienced extensive loss of wages. When the caregivers did take short time off work, they reported using paid sick leave, holidays or reorganizing their work schedules. | *‘. . . my daughter is a nurse and is well protected in terms of salary insurance. Even if she is off work for a long period, she is not at work, she doesn’t have to worry at all. That’s important because compared to me I had no insurance at all and I had to go to work during that time. I took some days off, but when I went to work, I felt really, really terrible.’ (PM)* | I had no insurance and had to continue working even though I felt sick | Insurance: continuing to work to maintain it despite health status |
|  |  |  | I lost some income due to days off work | Perceived reduction in earnings due to absence from work |
|  |  |  | I have decided to permanently reduce my working hours. | Job changes affecting earnings |
|  |  |  | The loss of income was due to my working condition and the length of the period of absence | Perceived reduction in earnings due to absence from work and type of work |
|  |  |  | I combined different types of compensation, and at certain stages I received no compensation and had to use my savings | Depletion of savings, assets, pension funds |
|  |  |  | Being a freelancer, I did not have insurance | Insurance: not having coverage because of the type of employment contract |
|  |  |  | My caregiver was not absent from work for long periods and did not perceive a massive loss of earnings | Working condition of caregiver has not changed |
| **Lauzier (2005)** | **Reorganization of daily and home life.**  Patients were helped in numerous spheres of their lives by several people. Help was provided primarily with domestic chores, transportation to treatments and moral support. The type and intensity of this help varied according to the phase of the disease, treatments and type of caregiver. At various points during the treatment period, some patients used, or made more intensive use than previously, of paid home care services because of a lack of time, fatigue and lessened physical capacity. For instance, they hired people to do housework, prepare meals and look after the children. One older patient stayed in a private convalescent home after surgery because she did not want to be home alone. Some patients mentioned that they would have liked to have help in the home but that they were not able to afford it. A woman had to forgo this service during the treatment period because of insufficient income to cover the cost (Box 2, quote 4). | *‘Me, I had a cleaning lady before and now when I would need one, I can’t afford it.’ (PQ* | Previously I had a domestic helper for housekeeping, and when I would (really) need it, I could not afford it financially | Reduction in spending on basic necessities |
|  |  |  | I hired a domestic worker to help me prepare meals and look after the children because I had less time and felt more tired. | Incurring non-medical expenses |
| **Lauzier (2005)** | **Coping with the disease.**  Several patients had costs which they said were the direct result of the psychological impact of the disease. They bought things and participated in activities that they would not have done or would have done at another time had they not had breast cancer. For instance, some patients went on outings or trips to take their mind off things. Some patients also gave gifts to their caregivers and volunteers to show their appreciation for all they had done (Box 2, quote 5). Trying to help reduce the psychological impact of the disease resulted also in this type of costs for the caregivers. For several patients and their caregivers, maintaining ties with family and friends during the illness was important. When the patients were treated at some distance from home or when their family and friends lived in another city, long distance calls and visits by out-of-town relatives resulted in increased costs. | *‘ - Well, I paid for treatments for my caregiver. Massage therapy treatments because I thought she could need them [laughter]. I thought she looked exhausted, pretty tired [laughter].’* | I paid for massages for my caregiver to reciprocate what he had done | Expenses for unnecessary goods |
|  |  |  | Maintaining relationships with family and friends required increased costs when I had treatment in another city | Incur non-medical expenses during treatment |
